# In utero exposure to economic fluctuations and birth outcomes: An analysis of the relevance of the local unemployment rate in Brazilian state capitals

**DOI:** 10.1371/journal.pone.0223673

**Published:** 2019-10-10

**Authors:** Matias Mrejen, Danielle Carusi Machado

**Affiliations:** Department of Economics, Universidade Federal Fluminense, Niterói, Rio de Janeiro, Brazil; University of Oslo, NORWAY

## Abstract

**Objective:**

Analyze if in utero exposure to economic downturns is associated with worsened birth outcomes.

**Methods:**

We used birth records from all live singleton births in the 27 Brazilian state capitals between October 2012 and December 2016 (n = 2,952,430) and linked them to local unemployment rates according to the mother’s residence. We estimated the association between different birth outcomes and the local unemployment rate in the three trimesters before birth. We included maternal characteristics and month, year and municipality fixed effects as covariates. We also estimated the association for different groups of mothers, based on marital status, educational level, age and race.

**Results:**

A 1 p.p. increase in the local unemployment rate in the trimester before birth is associated with 2.68% higher odds of being born with very low birthweight (< 1500 grams) (OR: 1.0268, 95% CI: 1.0006–1.0536). That result is pushed by the effect among newborns from mothers younger than 24 (OR: 1.0684, 95%CI: 1.0353–1.1024), from mothers with 11 years of schooling or less (OR: 1.0477, 95% CI: 1.0245–1.0714), and from brown or black mothers (OR: 1.0387, 95%CI: 1.0156–1.0624). The associations among children born from younger, less educated and black or brown mothers are robust to the application of a procedure to control for multiple testing, albeit the results considering the whole sample are not.

**Conclusions:**

Our study shows that there is an association between in utero exposure to higher unemployment rates during the last gestational trimester and the odds of being born with VLBW among children born from mothers younger than 24 years old, with less of 11 years of education and black or brown. These results suggest that children born from women of low socioeconomic status are more vulnerable to in utero exposure to economic downturns.

## Introduction

Determinants of health at birth have been an important concern in biomedical sciences and epidemiology for a long time [1]. More recently, social scientists, predominantly economists, have also focused on assessing the consequences of prenatal environment [2–3]. It has been shown that not only does prenatal environment affect birth outcomes, but it is also related with health condition and educational achievements later in life [3–4].

Different kinds of socio-economic and environmental aggregate-level shocks during gestation have been proven to have a significant effect on birth outcomes, e.g. armed violence [5], air pollution [6], and natural disasters [7]. Among them, economic downturns have attracted attention from many studies. Most studies have shown a positive relationship between maternal exposure to acute economic downturns [8–10] or regular economic cycle contractions [9–12] during pregnancy and adverse birth outcomes. However, some studies have shown a negative association [13], presumably due to selection effects. Some of those studies have used negative variations in the unemployment rate at subnational levels as an indicator of economic slowdowns [9, 11–13].

Most frequently studied birth outcomes are birthweight, gestational age and intrauterine growth restriction. Low birthweight (LBW, < 2500 grams), preterm birth (PTB, < 37 weeks of gestation), and the newborn being small-for-gestational-age (SGA, birthweight beneath the 10^th^ percentile of the weight for gestational age distribution) are the main causes of neonatal death among children born without congenital anomalies [14] and are associated with adverse health later in life [15–17].

It is possible to think about three different mechanisms through which pre-natal economic environment might affect birth outcomes: selection effects, maternal stress, and maternal nutrition. One kind of selection effect is the selection-into-motherhood caused by the differential impact of economic downturns on fertility decisions among societal groups. Economic downturns might affect fertility decisions of prospective mothers with different observable or unobservable characteristics. If, for example, economic downturns lead to a relative decrease in pregnancy rates among women with higher risk of having early term pregnancies or underweighted babies, we would observe a negative association between economic slowdowns and adverse birth outcomes. A seminal study that analyzed births in the United States between 1975 and 1999 showed that unemployment rates in the year before conception were associated with decreased rates of low birthweight and reduced neonatal mortality and attributed this association to reduced fertility among black women with low education, i.e. the most vulnerable group in that country [13].

An additional kind of selection effect is in utero selection. Some studies have shown that heightened maternal stress caused by exposure to economic downturns is associated with pregnancy loss, mainly among male fetuses exposed during mid-pregnancy [18–19]. Even though the biological reasons are unclear, the empirical evidence of this in utero selection effect is ample and has also been found associated with in utero exposure to stressors other than economic downturns [20].

Exposure to economic downturns can lead to heightened maternal stress levels, caused by personal job loss, job loss by someone closely related or by the fear of job loss in the near future [21]. Prenatal maternal stress is thought to be related with health at birth through the hypothalamic–pituitary–adrenal axis (HPA axis). When a pregnant woman is exposed to stress, the HPA axis is activated and heightens the levels of cortisol, which in turn leads to higher levels of placental corticotrophin-releasing hormone (CRH). Increased levels of CRH are associated with decreased fetal growth and pre-term delivery [22–23]. Even though this mechanism is widely accepted as relevant, it is possible that heightened maternal stress during pregnancy affects birth outcomes also through other mechanisms, like a depressed immune system, which increases the risk of inflammatory reactions that might lead to pre-term birth [23–24]. Also, higher stress levels might lead to prejudicial habits or health behaviors, like smoking or drinking alcohol, which could also be prejudicial for the newborn [23].

Additionally, economic downturns might lead to job losses or income reduction, which can diminish the consumption of health enhancing products, mainly a nutritionally healthy diet [21]. The gestational period has specific requirements in terms of caloric intake, as well as intake of essential nutrients like proteins, fatty acids and folate; and a wide array of studies have shown positive association between appropriate nutrition and a reduction in adverse birth outcomes [25].

In population-based observational studies, it is usually not possible to disentangle the specific mechanism through which in utero exposure to an adverse economic environment affects birth outcomes. Administrative data on birth records usually do not include any direct measure of either maternal stress or nutrition during pregnancy. In face of those data constraints, different studies have used the timing of exposure as an indirect way of identifying the causal mechanism. Adverse birth outcomes linked to maternal exposure to economic downturns at the beginning of the gestation -i.e., the first trimester- are considered an indicator of the stress-induced pathway [8–10]. Adverse birth outcomes linked to exposure during the last trimester of gestation are interpreted as an indicator of nutritional constraints affecting birth outcomes [10, 26]. However, there are contending arguments to the validity of that strategy, as evidence on the specific time window in which stress or nutritional shocks during pregnancy affect birth outcomes is not conclusive [23–25].

Some specific studies on the impact of in utero exposure to economic slowdowns deserve comment. A study on the impact of in utero exposure to the 2008 financial crisis in Iceland [8] found that it led to a reduction in birthweight, an increased probability of LBW and a reduction in the number of boys born -which is consistent with the in-utero selection mechanism- when it happened during the first trimester of gestation. The author suggests the maternal stress mechanism as possible explanation.

A study on economic cyclical fluctuations in Argentina between the years 2000 and 2005 [10] found that average birthweight decreases and the probabilities of being born with LBW increases when the economic activity slows down in the period 9 to 7 months before birth, which the authors judge as consistent with the stress hypothesis. The same study found that exposure of less educated mothers, who are presumably more financially vulnerable, to economic downturns in the three months before birth is also associated with lower birthweights and higher probabilities of being born with LBW. The authors interpret that second finding as an indication of the nutritional mechanism.

An additional study used changes in the state-level unemployment rate in the United States 1990 and 2013 as an indicator of economic slowdowns [9]. The authors found that an increase in the unemployment rate during the first trimester of pregnancy was associated with increased odds of PTB, and that relationship was larger during the 2007–2009 financial crisis. Increases in the unemployment rate during the second trimester of gestation were found to be associated with decreased odds of PTB. According to the authors, unreported results pointed in the same direction considering LBW as the outcome variable.

A similar study analyzed the association between variations in state level unemployment and birth outcomes in the United States between 1976 and 2016 [12]. The authors found that a one percentage point increase in the state unemployment rate during the first gestational trimester increases 0.1 percentage points the probability of preterm birth, but a one percentage point increase in the state unemployment rate during the second and third trimester reduces the probability of prematurity by 0.06 percentage points. They interpret the results as being possibly driven by two contrasting effects of economic downturns on maternal health: while recessions increase maternal exposure to socio-economic stressors like income or job loss, they also diminish mothers’ exposure to work related and environmental stressors. According to them, it is probable that hazardous effects dominate protective effects during the first gestational trimester, but not in later stages of pregnancy. It is also worth noting that the authors found heterogeneity in the results among different groups: among less educated black women the association was around three times larger in magnitude than among more educated white women.

The goal of our study was to analyze the association between economic environment during the pregnancy and birth outcomes in Brazil. We seek to contribute both to the literature on the impact of in utero exposure to economic downturns on birth outcomes [8–11] and to the literature on the effects of environmental factors during pregnancy on health at birth in the Brazilian context [27–30]. We used quarterly data on urban unemployment rates in the 27 Brazilian state capitals and linked it to birth records from the Brazilian vital statistics system, according to the mother’s municipality of residence, to estimate the association between the local unemployment rate during pregnancy and birth outcomes. Additionally, we measured the unemployment rate at different times of the pregnancy to evaluate if the timing of exposure to variations in local unemployment rates mattered and if there were problems of selection into motherhood or in utero selection.

We found that a 1 percentage point increase in the local unemployment rate in the trimester before birth is associated with 2.68% higher odds of being born with very low birthweight (VLBW, < 1500 grams) (OR: 1.0268, 95% CI: 1.0006–1.0536). That result is pushed by the effect among newborns from mothers younger than 24 (6.84% higher odds of being born with VLBW, OR: 1.0684, 95%CI: 1.0353–1.1024), from mothers with 11 years of schooling or less (4.77% higher odds of being born with VLBW, OR: 1.0477, 95% CI: 1.0245–1.0714), and from brown or black mothers (3.87% higher odds of being born with VLBW, OR: 1.0387, 95%CI: 1.0156–1.0624). The association among children born from younger, less educated and black or brown mothers are robust to the application of a procedure controlling for multiple testing, albeit the results considering the whole sample are not. These results suggest that children born from women of low socioeconomic status are more vulnerable to in utero exposure to economic downturns.

## Methods

### Data

In order to assess the birth outcomes, we used microdata from the SINASC-DATASUS, the System of Information on Live Births from the Department of Informatics of the *Sistema Unico de Saúde*, i.e., the Brazilian National Health Service. Data are open access and available in: http://datasus.saude.gov.br/informacoes-de-saude/servicos2/transferencia-de-arquivos (last accessed: 13/08/2018). Data are duly anonymized before being released. Each observation corresponds to one live birth and provides information on the pregnancy, newborn, and maternal characteristics. Registration of birth information on the system is mandatory for all births in the Brazilian territory.

We first selected data from all births between 2012 and 2016 from mothers residing in the 27 state capitals (n = 3,564,044), because those are the years for which both unemployment rates at the subnational level and data from live births are available. We kept data only from singleton births (n = 3,478,142), because birth outcomes in multiple births, specially birthweight, are significantly different. We then merged the data with the estimated unemployment rates in the mother’s city of residence. Information of unemployment at the municipality level is only available with quarterly frequency for the capitals of the 27 states, which are the units of the Federative Republic of Brazil. As data on local levels of unemployment are available from January 2012, we had to drop all births previous to October 2012 to have the local unemployment rate in the 9 months before the month of birth. Our final sample consisted on data of 2,952,430 live singleton births from mothers residing in any of the 27 Brazilian state capitals, born between October 2012 and December 2016.

#### Dependent variable

We used data on the following outcome variables: birthweight, gestational age, and sex. We considered sex as an outcome variable because there is evidence of a link between stress inducing events and a decline in the proportion of male births [18–19]. We kept birthweight (in grams) and gestational age (in weeks) as numerical variables. All cases in which one of these variables was coded as “Ignored” were recoded as a missing value, and therefore dropped from regressions. The “sex” variable was recoded as a binary variable indicating if the newborn was female or not (“female”). We created also binary variables for low birthweight (LBW, <2500 grams), very low birthweight (VLBW, <1500 grams), preterm birth (PTB, <37 weeks of gestation), very preterm birth (VPTB, <32 weeks), and small for gestational age (SGA, birthweight beneath the 10^th^ percentile of weight for gestational age, according to the “International Newborn Size Standards” of the INTERGROWTH-21st Project [31]).

#### Explanatory variable

Our explanatory variable is the mean local unemployment rate in the city where the mother resided during the pregnancy. We constructed that variable as the mean unemployment rate in the 9 months before the month of birth in the city (state capital) where the mother resided. We used the quarterly unemployment rate estimated by the Brazilian Institute of Geography and Statistics (IBGE), using the Continuous National Household Sample Survey (PNAD *Contínua*), for the 27 state capitals. Data are available on the website of the Brazilian Institute for Geography and Statistics (IBGE): https://sidra.ibge.gov.br/tabela/4099#/n6/all/v/4099/p/first%2020/d/v4099%201/l/v,p,t/resultado (last accessed: 10/04/2019).

As the original data are not available with a monthly frequency, we constructed a mean unemployment rate in the 9 months before the month of birth as a weighted mean of quarterly local unemployment rates. For newborns born in January, April, July, and October, the mean unemployment rate coincided with the mean of the previous three quarterly unemployment rates. For newborns conceived in other months, we estimated a weighted mean of the unemployment rate in the quarter of conception and the three previous ones. For example, the mean unemployment rate for a newborn conceived in February 2014 was the weighted mean of the unemployment rate in the first quarter of 2014 (with weight of 2/3, to account for February and March), the second and third quarter of 2014 (both with weight of 1, to account for the months from April to September), and the fourth quarter of 2014 (with weight of 1/3, to account for October). We did the same for the months 1 to 3, 4 to 6, and 7 to 9 before birth to approximate the unemployment rate in the mother’s city of residence during each trimester of gestation.

#### Covariates

We included as covariates several maternal characteristics, namely: race (white, black, Asian, brown, native, ignored), years of education (none, 1 to 3, 4 to 7, 8 to 11, 12 or more, ignored), marital status (single, married, widow, divorced, stable union, ignored). All missing values in these categorical variables were categorized with the pre-existing category “ignored” to avoid losing those observations in our regressions. In two different robustness checks, we tried both recoding all values originally categorized as “ignored” as missing values and keeping data exactly as original.

Some clarification about the race variable in the Brazilian context is needed. “Brown” is the literal translation of the “pardo” category, which is used to refer to individuals of multiracial background. According to official estimations for the last quarter of 2016, 43.8% of Brazilians were white, 47.2% brown, and 8.2 black. White Brazilians tend to be from higher socioeconomic status. This is reflected on inequalities in the labor market. Among working population, in the last quarter of 2016, mean monthly income was approximately $817 American dollars for white individuals, $449 for black, and $455 for brown. Unemployment rates were also divergent: 9.5% among white people, 14.4 among black people, and 14.1 among brown people [32].

We recoded the age of the mother (originally in years) and created four different binary variables: 19 years old or less, 20 to 24 years old, 25 to 34 years old, 35 years old or more. We also recoded the variables indicating the number of children dead or alive into two binary variables indicating if the mother had at least one other child dead or alive. In the Brazilian vital registration system, miscarriages are recorded as dead children.

We included also 12 fixed effects for the month of birth, 5 for the year of birth, and 27 for the mother’s municipality of residence, as well as the full set of interactions between fixed-effects.

#### Empirical strategy

To estimate the association between the unemployment rate in the mother’s city of residence during pregnancy and birth outcomes, we used a fixed-effects model of the following form:

$$y_{icma}=\alpha +\beta U_{cma}+X_{i}\gamma +M_{m}+A_{a}+C_{c}+\theta_{ca}+\nu_{ma}+\eta_{mc}+\varepsilon_{icma}$$ (1)

Where *y*_*icma*_ is one of the dependent variables described above, indicating birth outcomes for newborn *i*, born in the state capital *c*, in month *m*, and year *a*. *U*_*cma*_ is the estimated mean unemployment rate in the city *c* during the 9 months before the month *m* in the year *a*. The coefficient β, which measures the association between birth outcomes and unemployment rate during pregnancy, is the one of interest. *X*_*i*_ is a vector of maternal characteristics (age, race, years of education, marital status, previous children alive or deceased) for newborn *i*. *M*_*m*_ is a month-of-birth fixed effect to account for seasonal patterns in neonatal health. *A*_*a*_ is a year-of-birth fixed effect to account for possible shocks at the aggregate level. *C*_*c*_ is a fixed effect for each state capital city of residence of the mothers to control for underlying time-invariant differences among municipalities. θ_*ca*_ is an interaction term between the city of residence and the year fixed effects to account for time varying factors at the local level. ν_*ma*_ is an interaction term between the month and the year of birth fixed effects to account to time varying factors from month to month at the national level. η_*mc*_ is an interaction term between the month-of-birth and the city of residence fixed effect to account for local seasonality.

To analyze the timing of the association between variations in the unemployment rate during pregnancy and birth outcomes, we estimated also a fixed-effects model of the following form:

$$y_{icma}=\alpha +\sum_{T=1}^{3}\beta_{T}U_{Tcma}+X_{i}\gamma +M_{m}+A_{a}+C_{c}+\theta_{ca}+\nu_{ma}+\eta_{mc}+\varepsilon_{icma}$$ (2)

Where T = 1 indicates the months 7 to 9, T = 2 the months 4 to 6, and T = 3 the months 1 to 3 before the month of birth, and serves as proxy for the first, second and third gestational trimester, respectively. In all cases, we estimated the model by Ordinal Least Squares when the outcome variable was continuous (birthweight, gestational age) and by logistic regression (*logit* model) when the outcome variable was binary (LBW, VLBW, PTB, VPTB, SGA, female). Standards errors were clustered at the municipality level to correct for autocorrelation, as is recommended when working with data from repeated cross-sections [33–34] and was done in many previous studies similar to ours [5,8,10–13,27,30]. All analysis was performed with the software STATA 14/SE.

We estimated all models directly from individual observations, which is equivalent to using grouped data with cells defined by all existing combinations of values of the categorical variables, weighted by the frequency of each combination. For the cases estimated by OLS (birthweight and gestational age), the coefficients can be interpreted as the marginal effect of a 1 percentage point increase in the local unemployment rate. For the cases estimated by *logit*, we report the odds ratio, which show the change in the odds of the different birth outcomes associated with a 1 percentage point increase in the local unemployment rate.

## Results

### Descriptive analysis

Table 1 shows descriptive statistics for all live births from singleton pregnancies from mothers residing in Brazilian state capitals and in the rest of Brazil between October 2012 and December 2016. There are relevant differences both in birth outcomes and in maternal characteristics among the two groups. Our analysis is restricted only to births in state capitals, the only cities with available information on the local unemployment rate.

In state capitals, mean birthweight is smaller, and there is a higher proportion of newborns with LBW and VLBW. Mean gestational age is shorter and there is a higher proportion of VPTB. The proportion of PTB and newborns SGA is smaller.

**Table 1:** Summary statistics, live births in Brazilian state capitals and in Brazil (October 2012 –December 2016).

	State capitals	Rest of Brazil	Difference
**N =**	2,952,430	9,223,182	
**Birth outcomes**			
*Birthweight*			
Mean birthweight in grams (SD)	3191.8 (547.8)	3204.1 (536.4)	-12.3***
LBW (%)	7.83	7.22	0.61***
VLBW (%)	1.32	1.10	0.22***
Missing data (%)	0.00	0.03	-0.03***
*Gestational age*			
Mean gestational age in weeks (SD)	38.5 (2.1)	38.6 (2.2)	-0.1***
PTB (%)	10.15	10.62	-0.47***
VPTB (%)	1.44	1.39	0.05***
Missing data (%)	2.46	3.66	-1.2***
*Other birth outcomes*			
SGA (%)	7.04	7.47	-0.43***
Missing data (%)	2.46	3.70	-1.24***
Female (%)	48.74	48.75	-0.01
Missing data (%)	0.02	0.27	-0.25***
**Maternal characteristics**			
*Age (%)*			
≤ 19	15.49	19.70	-4.21***
20–24	23.02	26.04	-3.02***
25–34	46.13	42.88	3.25***
≥ 35	15.35	11.37	3.98***
Missing data	0.00	0.20	-0.2***
*Education (%)*			
None	0.20	0.75	-0.55***
1 to 3 years	1.58	3.61	-2.03***
4 to 7 years	14.71	21.14	-6.43***
8 to 11 years	56.74	57.91	-1.17***
12 years and more	25.72	14.73	10.99***
Ignored (includes missing data)	1.05	1.86	-0.81***
*Race (%)*			
Brown	54.85	53.09	1.76***
White	32.46	36.37	-3.91***
Black	6.15	4.83	1.32***
Asian	0.60	0.30	0.3***
Native	0.25	0.93	-0.68***
Ignored (includes missing data)	5.69	4.48	1.21***
*Marital Status (%)*			
Married	32.88	32.31	0.57***
Single	46.36	39.39	6.97***
Widow	0.14	0.19	-0.05***
Consensual Union	18.75	25.79	-7.04***
Divorced	1.07	1.05	0.02***
Ignored (includes missing data)	0.79	1.27	-0.48***
*Previous children (%)*			
At least 1 child alive	55.34	57.60	-2.26***
Missing data	7.41	5.54	1.87***
At least 1 child not alive	20.70	17.90	2.80***
Missing data	11.33	8.36	2.97***

*Note*: all data are from SINASC-DATASUS. LBW: low birthweight (< 2500 grams). VLBW: very low birthweight (< 1500 grams). PTB: preterm birth (<37 weeks). VPTB: very preterm birth (<32 weeks). SGA: small for gestational age (birthweight beneath the 10th percentile of weight for gestational age). Significance of the differences was estimated by a two-sample t-test for means (birthweight and gestational age) or a two-sample test of proportions (all other variables).

* p value < 0.1

** p value < 0.05

*** p value < 0.01.

Mothers in state capitals are comparatively older and more educated, and the proportion without a partner (single, widow or divorced) is higher. Among them, the proportion of black and brown mothers is higher than in the rest of the country; and the proportion of white mothers is smaller.

### Regression analysis

Table 2 shows the results of estimating model 1 (panel A) and model 2 (panel B). The association between a one percentage point (p.p.) increase in the mean local unemployment rate during pregnancy in the city (state capital) of residence of the mother and birth outcomes does not reach the 5% threshold of significance for any outcome (Table 2, panel A). Considering the mean unemployment rate in the months 1 to 3, 4 to 6, and 7 to 9 before birth -which serve as a proxy for the unemployment rate at the different gestational trimesters-, a 1 p.p. increase in the local unemployment rate in the trimester before birth is associated with a 1.8 grams lower birthweight (95% Confidence Interval: -3.42 - -0.17) and with 2.68% higher odds of being born with VLBW (OR: 1.0268, 95% CI: 1.0006–1.0536).

**Table 2:** Unemployment rate at the mother city of residence and birth outcomes.

	(1)	(2)	(3)	(4)	(5)	(6)	(7)	(8)
	Birthweight	LBW	VLBW	Gestational Age	PTB	VPTB	SGA	Female
**PANEL A**								
*Unemployment rate months 1 to 9 before birth*	-1.6593	1.0080	1.0100	-0.0120*	0.9973	1.0263	0.9914	0.9961
95% Confidence Interval	(-4.3402–1.0216)	(0.9937–1.0225)	(0.9770–1.0440)	(-0.0250–0.0011)	(0.9856–1.0091)	(0.9931–1.0606)	(0.9769–1.0061)	(0.9889–1.0033)
P value	0.2146	0.2749	0.5588	0.0712	0.6490	0.1223	0.2482	0.2874
Observations	2,594,223	2,594,223	2,594,223	2,552,977	2,555,192	2,555,192	2,579,539	2,593,733
**PANEL B**								
*Unemployment rate months 7 to 9 before birth*	-0.6581	1.0035	1.0099	-0.0064	0.9943	1.0100	0.9992	0.9995
95% Confidence Interval	(-2.4943–1.1781)	(0.9960–1.0110)	(0.9846–1.0358)	(-0.0148–0.0020)	(0.9831–1.0056)	(0.9940–1.0261)	(0.9885–1.0101)	(0.9949–1.0040)
P value	0.4679	0.3597	0.4468	0.1302	0.3215	0.2220	0.8915	0.8196
*Unemployment rate months 4 to 6 before birth*	0.3802	1.0010	0.9817	-0.0024	1.0057	0.9962	0.9900	0.9987
95% Confidence Interval	(-1.3624–2.1228)	(0.9910–1.0111)	(0.9577–1.0064)	(-0.0103–0.0055)	(0.9963–1.0151)	(0.9736–1.0192)	(0.9736–1.0067)	(0.9920–1.0054)
P value	0.6575	0.8489	0.1445	0.5424	0.2337	0.7416	0.2393	0.7059
*Unemployment rate months 1 to 3 before birth*	-1.7953**	1.0040	1.0268**	-0.0029	0.9962	1.0265*	1.0045	0.9975
95% Confidence Interval	(-3.4172 - -0.1735)	(0.9905–1.0177)	(1.0006–1.0536)	(-0.0128–0.0070)	(0.9851–1.0075)	(0.9988–1.0549)	(0.9922–1.0171)	(0.9916–1.0035)
P value	0.0314	0.5650	0.0447	0.5553	0.5126	0.0613	0.4725	0.4169
Observations	2,594,223	2,594,223	2,594,223	2,552,977	2,555,192	2,555,192	2,579,539	2,593,733

*Note*: The table shows the association between the mean local unemployment rate in the 9 months (Panel A) and in the months 1 to 3, 4 to 6 and 7 to 9 (Panel B) before birth in the city where the mother resided and different birth outcomes. Standard errors are clustered at the city level. LBW: low birthweight (< 2500 grams). VLBW: very low birthweight (< 1500 grams). PTB: preterm birth (<37 weeks). VPTB: very preterm birth (<32 weeks). SGA: small for gestational age (birthweight beneath the 10th percentile of weight for gestational age). All regressions included as covariates binary variables indicating: age of the mother (younger than 19 years old, between 20 and 24, between 25 and 34, older than 35), marital status of the mother (single, married, widow, divorced, consensual union, ignored), race/ethnicity of the mother (Asian, white, native, brown, black), years of education of the mother (None, 1 to 3, 4 to 7, 8 to 11, 12 or more), the mother had a previous child alive, the mother had a previous child dead. All regressions included fixed effects for: mother city of residence, month of birth, year of birth. All regressions included two-way interactions between the fixed effects. S1 Table presents an extended version of Table 2, including the coefficients estimated for all covariates, except interaction terms.

* p value < 0.1

** p value < 0.05

*** p value < 0.01.

Table 3 shows the results of some robustness exercises for the results found in the estimation of model 2. We only used birthweight related outcome variables, as they are the only ones for which we found associations with the local unemployment rate during pregnancy. We ran the same regression of columns 1, 2 and 3 of panel B of Table 2, but with alternative ways of managing the missing values in three maternal characteristics: level of education, race, and marital status. Either recategorizing values “ignored” as missing values or leaving “ignored” values and missing values as in the original data did not substantially alter our original results. We also ran the regressions without considering the race of the mother, because it is the covariate with the largest proportion of missing data or classified as ignored, and it did not alter our results either. The association between the mean unemployment rate in the mother city of residence during the three months before birth and the odds of being born with VLBW remained robust and, therefore, we further focused exclusively on this outcome.

**Table 3:** Unemployment rate at the mother city of residence and birth outcomes, robustness checks.

	Ignored as missing			Same as original			Without controlling for race		
	(1)	(2)	(3)	(4)	(5)	(6)	(7)	(8)	(9)
	Birthweight	LBW	VLBW	Birthweight	LBW	VLBW	Birthweight	LBW	VLBW
*Unemployment rate months 7 to 9 before birth*	-0.5242	1.0015	1.0108	-0.5014	1.0019	1.0083	-0.6140	1.0034	1.0100
95% Confidence Interval	(-2.4335–1.3851)	(0.9943–1.0088)	(0.9858–1.0365)	(-2.4040–1.4012)	(0.9948–1.0091)	(0.9835–1.0338)	(-2.4610–1.2331)	(0.9959–1.0110)	(0.9846–1.0360)
P value	0.5774	0.6827	0.4004	0.5927	0.5987	0.5140	0.5005	0.3714	0.4437
*Unemployment rate months 4 to 6 before birth*	0.5697	0.9992	0.9822	0.4524	0.9994	0.9840	0.3750	1.0010	0.9818
95% Confidence Interval	(-1.0923–2.2317)	(0.9895–1.0090)	(0.9566–1.0085)	(-1.2511–2.1559)	(0.9897–1.0092)	(0.9586–1.0100)	(-1.3577–2.1077)	(0.9911–1.0109)	(0.9578–1.0064)
P value	0.4873	0.8746	0.1821	0.5898	0.9084	0.2258	0.6601	0.8443	0.1451
*Unemployment rate months 1 to 3 before birth*	-1.6919*	1.0055	1.0268**	-1.7921**	1.0061	1.0278**	-1.7360**	1.0038	1.0263**
95% Confidence Interval	(-3.4614–0.0776)	(0.9930–1.0182)	(1.0016–1.0528)	(-3.5706 - -0.0137)	(0.9936–1.0188)	(1.0030–1.0532)	(-3.3702 - -0.1018)	(0.9903–1.0175)	(1.0001–1.0532)
P value	0.0601	0.3909	0.0372	0.0484	0.3420	0.0278	0.0382	0.5806	0.0493
Observations	2,457,972	2,457,972	2,457,972	2,474,646	2,474,646	2,474,646	2,594,223	2,594,223	2,594,223

*Note*: The table shows the association between the mean local unemployment rate in the months 1 to 3, 4 to 6 and 7 to 9 before birth in the city where the mother resided and birth outcomes. Standard errors are clustered at the city level. LBW: low birthweight (< 2500 grams). VLBW: very low birthweight (< 1500 grams). All regressions included as covariates binary variables indicating: the age, marital status, education, and previous children alive or not of the mother; as well as fixed effects for mother city of residence, month of birth and year of birth, and two-way interactions between the fixed effects. Columns 1 to 6 also included the race of the mother as a covariate. In columns 1 to 3, all observations with race, marital status or education classified as ignored were recoded as missing values. In columns 4 to 6, all “ignored” and missing values in those three variables were kept as in the original data. S2 Table presents an extended version of Table 3, including the coefficients estimated for all covariates, except interaction terms.

* p value < 0.1

** p value < 0.05

*** p value < 0.01.

Table 4 shows the results of estimating model 2 for different groups of mothers, to see if there are heterogeneities in the association between in utero exposure to variations in the local unemployment rate and VLBW, i.e. the birth outcome for which we found an association with the local unemployment rate during pregnancy. No relevant differences were found when we segmented the sample between children from mothers with a partner (married or in a consensual union) and from mothers without a partner (single, divorced or widow).

**Table 4:** Unemployment rate at the mother city of residence and VLBW.

	Age		Marital status		Race/ethnicity		Years of education	
	≤ 24 years	≥ 25 years	Partner	No Partner	Black or Brown	White	11 or less	12 or more
	(1)	(2)	(3)	(4)	(5)	(6)	(7)	(8)
	VLBW	VLBW	VLBW	VLBW	VLBW	VLBW	VLBW	VLBW
*Unemployment rate months 7 to 9 before birth*	1.0523**	0.9815	1.0195	1.0019	1.0111	0.9858	1.0224	0.9788
95% Confidence Interval	(1.0115–1.0948)	(0.9526–1.0114)	(0.9941–1.0456)	(0.9676–1.0373)	(0.9858–1.0371)	(0.9326–1.0421)	(0.9918–1.0539)	(0.9389–1.0203)
P value	0.0116	0.2228	0.1338	0.9169	0.3938	0.6145	0.1528	0.3120
*Unemployment rate months 4 to 6 before birth*	0.9636	0.9929	0.9843	0.9789	0.9880	0.9754	0.9740*	1.0104
95% Confidence Interval	(0.9136–1.0164)	(0.9617–1.0252)	(0.9436–1.0268)	(0.9451–1.0139)	(0.9604–1.0164)	(0.9153–1.0395)	(0.9440–1.0049)	(0.9360–1.0907)
P value	0.1732	0.6635	0.4626	0.2348	0.4036	0.4430	0.0986	0.7912
*Unemployment rate months 1 to 3 before birth*	1.0684***	0.9992	1.0354*	1.0080	1.0387***	0.9946	1.0477***	0.9561
95% Confidence Interval	(1.0353–1.1024)	(0.9633–1.0364)	(0.9938–1.0787)	(0.9781–1.0388)	(1.0156–1.0624)	(0.9247–1.0697)	(1.0245–1.0714)	(0.8934–1.0233)
P value	0.0000	0.9650	0.0965	0.6049	0.0009	0.8834	0.0000	0.1950
Observations	975,113	1,619,110	1,339,859	1,236,633	1,550,041	910,334	1,903,439	667,077

*Note*: The table shows the association between the mean local unemployment rate in the months 1 to 3, 4 to 6 and 7 to 9 before birth in the city where the mother resided for different groups of mothers. Standard errors are clustered at the city level. VLBW: very low birthweight (< 1500 grams). All regressions included covariates for previous children alive or not, fixed effects for mother city of residence, month of birth and year of birth, and two-way interactions between the fixed effects. Columns 1 and 2 included also marital status, race and education. Columns 3 and 4 included also age, race and education. Columns 5 and 6 included also age, marital status and education. Columns 7 and 8 included age, marital status, and race. S3 Table presents an extended version of Table 4, including the coefficients estimated for all covariates, except interaction terms.

* p value < 0.1

** p value < 0.05

*** p value < 0.01.

Among newborns from mothers younger than 24, a 1 p.p. increase in the mean unemployment rate in the mother’s city of residence during the months 9 to 7 before birth is associated with 5.23% higher odds of being born with VLBW (OR: 1.0523, 95%CI: 1.0115–1.0948). The mean unemployment rate in the last trimester before birth is associated with 6.84% higher odds of being born very underweighted (OR: 1.0684, 95%CI: 1.0353–1.1024). No significant association was found among newborns from mothers 25 or older.

Considering level of education, among newborns from mothers with 11 years of schooling or less a 1 p.p. increase in the local unemployment rate during the months 1 to 3 before the month of birth was associated with 4.77% higher odds of being born with VLBW (OR: 1.0477, 95% CI: 1.0245–1.0714). No significant association was found among newborns from mothers with 12 or more years of education.

We also analyzed the association between in utero exposure to fluctuations in the local unemployment rate and the odds of being born with VLBW separately for children born from white and for black or brown mothers. The rationale for that separation is that, during our period of analysis, mean income from work and unemployment rates among the black and brown population were similar and notably worse than among the white population [32]. Children from Asian and native mothers were not considered because they were a very small proportion of our data. Among children from brown of black mothers, a 1 p.p. increase in the unemployment rate during the last trimester before birth was associated with 3.87% higher odds of being born with VLBW (OR: 1.0387, 95%CI: 1.0156–1.0624). No significant association was found among newborns from white mothers, although it is worth noting that the sign of the association is the opposite.

Unemployment rates could differentially affect the fertility decisions made by women and, therefore, introduce a selection bias. If that were the case, the odds of the mother having a certain characteristic would change in response to changes in the local unemployment rate. We regressed different maternal characteristics on the local unemployment rate in the months 10 to 18 (Table 5 - Panel A) and in the months 10 to 12 (Table 5 - Panel B) before birth to check if that was the case in our data. As quarterly unemployment rates at the local level were available from beginning 2012, we had to drop all births before July 2013 to have data from unemployment rates at the mother city of residence in months 10 to 18 before birth. The results, depicted in Table 5, show no significative association between the local unemployment rate dynamics before conception and the odds of the mother: being younger or older than 24 years old, having a stable partner or not, being black or brown or being white, having up to 11 years of education or 12 or more. Granted, we cannot discard the existence of a selection into motherhood phenomenon according to non-observable characteristics.

**Table 5:** Unemployment rate before conception and selection into motherhood.

	Age		Marital status		Race/ethnicity		Education	
	(1)	(2)	(3)	(4)	(5)	(6)	(7)	(8)
	< = 24 years	> = 25 years	Partner	No Partner	Black or Brown	White	11 years or less	12 years or more
**PANEL A**								
*Unemployment rate months 10 to 18 before birth*	0.9974	1.0026	0.9923	1.0078	1.0171	0.9869	0.9994	1.0006
95% Confidence Interval	(0.9873–1.0077)	(0.9924–1.0129)	(0.9676–1.0175)	(0.9828–1.0334)	(0.9931–1.0418)	(0.9644–1.0099)	(0.9849–1.0141)	(0.9861–1.0154)
P value	0.6203	0.6203	0.5449	0.5449	0.1646	0.2627	0.9314	0.9314
Observations	2,427,273	2,427,273	2,408,605	2,408,605	2,289,931	2,289,931	2,404,774	2,404,774
**PANEL B**								
*Unemployment rate months 10 to 12 before birth*	0.9963	1.0037	1.0029	0.9971	0.9975	1.0032	0.9954	1.0047
95% Confidence Interval	(0.9915–1.0010)	(0.9990–1.0085)	(0.9879–1.0181)	(0.9822–1.0122)	(0.9834–1.0119)	(0.9899–1.0166)	(0.9882–1.0026)	(0.9974–1.0119)
P value	0.1260	0.1260	0.7052	0.7052	0.7340	0.6410	0.2064	0.2064
Observations	2,427,273	2,427,273	2,408,605	2,408,605	2,289,931	2,289,931	2,404,774	2,404,774

*Note*: The table shows the association between the mean local unemployment rate in the months 10 to 18 (Panel A) and 10 to 12 (Panel B) before birth in the city where the mother resided and different maternal characteristics. Standard errors are clustered at the city level. All regressions included fixed effects for mother city of residence, month of birth and year of birth, and two-way interactions between the fixed effects. S4 Table presents an extended version of Table 5, including the coefficients estimated for all covariates, except interaction terms.

* p value < 0.1

** p value < 0.05

*** p value < 0.01.

A frequently cited problem with studies that report many tests, such as ours, is that the probabilities of rejecting the null hypothesis increase with the number of regressions. To assess to which extent our results were affected by multiple testing, we applied the false discovery rate controlling procedure suggested by Benjamini and Hochberg [35]. The results can be found in S5 Table. Adopting a false discovery rate (i.e., the expected proportion of errors among the rejected null hypotheses) of 0.05, we found that our main results from Table 4 stay significant. That gives us confidence that the association found between economic downturns during the last trimester of pregnancy and higher odds of being born with VLBW among children born from less educated, black or brown and younger women is not a result of multiple testing. The association found between exposure during the last trimester and higher odds of being born with VLBW for the whole sample did not stand the false discovery rate controlling procedure.

## Discussion

Our results show that increases in local unemployment rates during pregnancy are associated with lower birthweight and higher odds of being born with VLBW among newborns from mothers who resided in the 27 Brazilian state capitals. The association is detectable in the last trimester before birth among newborns from younger, less educated and black or brown mothers. Our results also show that local unemployment rates before conception are not correlated with maternal characteristics, which suggests that our results are not biased by a selection-into-motherhood effect.

Our results are in line with previous results from economic downturns in a developing country context that suggested a positive effect of economic downturns during the third gestational trimester on the prevalence of LBW among children born from less educated mothers [10]. In the Brazilian context, younger, less educated and brown or black mothers are more likely to be from low socioeconomic status and, therefore, their children are more likely to have worse birth outcomes [29]. Our results suggest that their children are also more vulnerable to in utero exposure to economic downturns.

There are some limitations of our study that should be mentioned. First, the way we constructed the local unemployment rate in the months 1 to 9 before the month of birth introduced some measurement error because we lacked monthly data. Second, as gestational lengths differ from pregnancy to pregnancy, the unemployment rate in the months 1 to 9 before birth might capture periods before conception. This is the case of shorter pregnancies and the local unemployment rate in the months 7 to 9 before birth. For example, for a pregnancy that lasts 7 months, the unemployment rate in the months 7 to 9 is capturing labor market dynamics in moments before conception. This could introduce a downward bias in our estimates for the association between variations in the local unemployment rate during the months 7 to 9 before birth and birth outcomes. If the bias was large enough, we could be wrongly failing to reject the null hypothesis of no association.

An additional limitation is that we cannot conclusively identify a causal path through which in utero exposure to higher unemployment rates results in higher odds of being born with VLBW. The timing of exposure has been used as a proxy to identify the causal path [8–10], but there are some concerns about the conclusiveness of that strategy [21, 25]. Therefore, the association found deserves further studies to identify the ways through which in utero exposure to economic downturns affects health at birth. Also, the fact that we did not find any association between local unemployment rates at the mother city of residence before conception and maternal characteristics suggests that there is not a selection-into-motherhood effect, but we cannot discard that there is a selection according to unobservable characteristics.

Finally, another limitation is that local unemployment rate dynamics might be capturing changes in another highly correlated variable which could be more relevant. We controlled for month and year fixed effects specific to each municipality of residence of the mother, as well as for an interaction between month and year of birth fixed effects. This allowed us to control for seasonality, yearly changes in other factors, and monthly changes at the national level. However, there might be monthly changes at the local level of variables other than the unemployment rate that could be relevant, e.g. the mean work income or the local level of economic activity.

Despite those limitations, our study shows that there is an association between in utero exposure to higher unemployment rates and worsened birth outcomes, specifically higher odds of being born with very low birthweight when exposure happens during the last gestational trimester. Exposure affects children born from mothers younger than 24 years old, with less of 11 years of education and black or brown. That means that newborns from women of low socioeconomic status have higher odds of being born very underweighted when exposed to economic downturns in the last months of the gestational period. This result calls for further studies on the impact of economic conditions on birth outcomes and on possible buffering mechanisms.

## Supporting information

S1 Table Extended version of Table 2. (PDF)

S2 Table Extended version of Table 3. (PDF)

S3 Table Extended version of Table 4. (PDF)

S4 Table Extended version of Table 5. (PDF)

S5 Table False discovery rate controlling procedure. (PDF)
