# Supplementary material for: In utero exposure to economic fluctuations and birth outcomes: An analysis of the relevance of the local unemployment rate in Brazilian state capitals
Source: PLoS One. 2019 Oct 10;14(10):e0223673. doi: 10.1371/journal.pone.0223673 (PMC6786569; doi:10.1371/journal.pone.0223673)
Supplement: S1 Table — (PDF) [file pone.0223673.s001.pdf]

## S1 Table. Extended version of table 2.

| PANEL A | (1) | (2) | (3) | (4) | (5) | (6) | (7) | (8) |
|---|---|---|---|---|---|---|---|---|
| | Birthweight | LBW | VLBW | Gestational Age | PTB | VPTB | SGA | Female |
| Unemployment rate months 1 to 9 before birth | -1.6593 | 1.0080 | 1.0100 | -0.0120* | 0.9973 | 1.0263 | 0.9914 | 0.9961 |
| 95% CI | (-4.3402 - 1.0216) | (0.9937 - 1.0225) | (0.9770 - 1.0440) | (-0.0250 - 0.0011) | (0.9856 - 1.0091) | (0.9931 - 1.0606) | (0.9769 - 1.0061) | (0.9889 - 1.0033) |
| p-val | 0.2146 | 0.2749 | 0.5588 | 0.0712 | 0.6490 | 0.1223 | 0.2482 | 0.2874 |
| Maternal characteristics | | | | | | | | |
| Age | | | | | | | | |
| <= 19 | -65.9703*** | 1.0687*** | 0.9850 | -0.0865*** | 1.2025*** | 1.2256*** | 1.0104 | 0.9955 |
| | (-75.4581 - -56.4826) | (1.0354 - 1.1030) | (0.9463 - 1.0253) | (-0.1168 - -0.0562) | (1.1652 - 1.2410) | (1.1846 - 1.2681) | (0.9785 - 1.0433) | (0.9826 - 1.0086) |
| | 0.0000 | 0.0000 | 0.4607 | 0.0000 | 0.0000 | 0.0000 | 0.5266 | 0.4974 |
| 20-24 | -12.6392*** | 0.9242*** | 0.8745*** | 0.0943*** | 0.9664*** | 0.9477** | 0.9985 | 0.9992 |
| | (-18.4527 - -6.8258) | (0.9041 - 0.9447) | (0.8458 - 0.9041) | (0.0739 - 0.1146) | (0.9462 - 0.9870) | (0.9075 - 0.9897) | (0.9855 - 1.0117) | (0.9937 - 1.0047) |
| | 0.0001 | 0.0000 | 0.0000 | 0.0000 | 0.0015 | 0.0151 | 0.8245 | 0.7652 |
| 25-34 | | | | omitted | | | | |
| >= 35 | -35.9747*** | 1.2862*** | 1.3508*** | -0.2325*** | 1.2537*** | 1.2991*** | 1.1502*** | 1.0043 |
| | (-40.1425 - -31.8069) | (1.2614 - 1.3116) | (1.2780 - 1.4279) | (-0.2500 - -0.2149) | (1.2379 - 1.2696) | (1.2432 - 1.3574) | (1.1293 - 1.1714) | (0.9982 - 1.0104) |
| | 0.0000 | 0.0000 | 0.0000 | 0.0000 | 0.0000 | 0.0000 | 0.0000 | 0.1667 |
| Previous children | | | | | | | | |
| At least 1 alive | 101.4938*** | 0.6775*** | 0.6213*** | 0.0476*** | 0.8898*** | 0.7239*** | 0.6551*** | 1.0035* |
| | (93.2707 - 109.7169) | (0.6578 - 0.6977) | (0.5951 - 0.6486) | (0.0269 - 0.0683) | (0.8720 - 0.9080) | (0.6750 - 0.7764) | (0.6364 - 0.6744) | (0.9999 - 1.0071) |
| | 0.0000 | 0.0000 | 0.0000 | 0.0001 | 0.0000 | 0.0000 | 0.0000 | 0.0557 |
| At least 1 dead | -23.7267*** | 1.2466*** | 1.4793*** | -0.1339*** | 1.2006*** | 1.4262*** | 1.0597*** | 1.0009 |
| | (-29.3886 - -18.0648) | (1.2056 - 1.2890) | (1.4027 - 1.5602) | (-0.1574 - -0.1103) | (1.1656 - 1.2367) | (1.3380 - 1.5202) | (1.0377 - 1.0821) | (0.9946 - 1.0072) |
| | 0.0000 | 0.0000 | 0.0000 | 0.0000 | 0.0000 | 0.0000 | 0.0000 | 0.7794 |
| Education | | | | | | | | |
| None | -112.4286*** | 1.8363*** | 1.7275*** | 0.1204** | 1.3600*** | 1.6355*** | 2.3149*** | 1.0812*** |
| | (-140.6187 - -84.2386) | (1.6745 - 2.0139) | (1.3887 - 2.1490) | (0.0256 - 0.2152) | (1.2355 - 1.4971) | (1.3352 - 2.0033) | (2.1180 - 2.5301) | (1.0439 - 1.1199) |
| | 0.0000 | 0.0000 | 0.0000 | 0.0148 | 0.0000 | 0.0000 | 0.0000 | 0.0000 |
| 1 - 3 years | -51.6450*** | 1.5538*** | 1.3974*** | 0.2035*** | 1.2246*** | 1.3829*** | 2.0081*** | 1.0140 |
| | (-70.4655 - -32.8244) | (1.4686 - 1.6439) | (1.2823 - 1.5229) | (0.1581 - 0.2490) | (1.1671 - 1.2850) | (1.2574 - 1.5209) | (1.8915 - 2.1318) | (0.9935 - 1.0349) |
| | 0.0000 | 0.0000 | 0.0000 | 0.0000 | 0.0000 | 0.0000 | 0.0000 | 0.1821 |
| 4 - 7 years | -27.3847*** | 1.3771*** | 1.2654*** | 0.2057*** | 1.1736*** | 1.2730*** | 1.7320*** | 1.0116** |

| | | | | | | | | |
|---|---|---|---|---|---|---|---|---|
| | (-38.6839 - -16.0855) | (1.3177 - 1.4393) | (1.1865 - 1.3497) | (0.1777 - 0.2336) | (1.1429 - 1.2051) | (1.1923 - 1.3592) | (1.6439 - 1.8248) | (1.0013 - 1.0219) |
| | 0.0000 | 0.0000 | 0.0000 | 0.0000 | 0.0000 | 0.0000 | 0.0000 | 0.0269 |
| 8 - 11 years | 9.4745** | 1.1308*** | 1.2048*** | 0.2043*** | 1.0387*** | 1.1658*** | 1.3542*** | 1.0048** |
| | (2.2456 - 16.7034) | (1.1019 - 1.1604) | (1.1466 - 1.2660) | (0.1843 - 0.2243) | (1.0191 - 1.0587) | (1.1132 - 1.2209) | (1.3172 - 1.3923) | (1.0001 - 1.0096) |
| | 0.0122 | 0.0000 | 0.0000 | 0.0000 | 0.0001 | 0.0000 | 0.0000 | 0.0473 |
| 12 years or more | | | | omitted | | | | |
| Ignored | -15.3285 | 1.3564*** | 1.5846*** | 0.1133* | 1.2239*** | 1.5709*** | 1.4360*** | 1.0068 |
| | (-43.2460 - 12.5890) | (1.1752 - 1.5656) | (1.2627 - 1.9885) | (-0.0005 - 0.2271) | (1.0690 - 1.4012) | (1.2958 - 1.9043) | (1.2764 - 1.6155) | (0.9781 - 1.0364) |
| | 0.2694 | 0.0000 | 0.0001 | 0.0509 | 0.0034 | 0.0000 | 0.0000 | 0.6445 |
| Marital status | | | | | | | | |
| Single | | | | omitted | | | | |
| Married | 20.2979*** | 0.8517*** | 0.8178*** | -0.0673*** | 0.9222*** | 0.7681*** | 0.8146*** | 0.9983 |
| | (15.6787 - 24.9170) | (0.8400 - 0.8636) | (0.7874 - 0.8493) | (-0.0883 - -0.0463) | (0.9093 - 0.9354) | (0.7328 - 0.8051) | (0.8032 - 0.8261) | (0.9900 - 1.0067) |
| | 0.0000 | 0.0000 | 0.0000 | 0.0000 | 0.0000 | 0.0000 | 0.0000 | 0.6934 |
| Widow | 0.9983 | 1.0365 | 0.9810 | -0.0758* | 1.0606 | 0.9783 | 0.9415 | 1.0100 |
| | (-12.0880 - 14.0846) | (0.9400 - 1.1430) | (0.8059 - 1.1943) | (-0.1589 - 0.0074) | (0.9648 - 1.1658) | (0.7733 - 1.2375) | (0.8331 - 1.0639) | (0.9642 - 1.0579) |
| | 0.8766 | 0.4718 | 0.8487 | 0.0723 | 0.2233 | 0.8547 | 0.3336 | 0.6748 |
| Divorced | -1.6079 | 0.9767 | 0.9978 | -0.0907*** | 1.0130 | 0.9656 | 0.8843*** | 0.9828** |
| | (-6.5138 - 3.2980) | (0.9360 - 1.0191) | (0.9165 - 1.0864) | (-0.1191 - -0.0623) | (0.9773 - 1.0499) | (0.8879 - 1.0500) | (0.8555 - 0.9140) | (0.9662 - 0.9998) |
| | 0.5064 | 0.2766 | 0.9599 | 0.0000 | 0.4815 | 0.4128 | 0.0000 | 0.0471 |
| Consensual union | 15.1168*** | 0.9737 | 0.9656 | 0.0424** | 0.9849 | 0.9340 | 0.9672** | 1.0041 |
| | (8.0918 - 22.1418) | (0.9113 - 1.0404) | (0.8663 - 1.0763) | (0.0043 - 0.0805) | (0.9408 - 1.0311) | (0.8585 - 1.0162) | (0.9404 - 0.9948) | (0.9974 - 1.0109) |
| | 0.0002 | 0.4305 | 0.5275 | 0.0306 | 0.5150 | 0.1126 | 0.0202 | 0.2315 |
| Ignored | -6.5321 | 1.0357 | 1.1791** | -0.1024*** | 1.0631* | 1.1730** | 0.9524* | 0.9975 |
| | (-19.4759 - 6.4117) | (0.9626 - 1.1145) | (1.0336 - 1.3450) | (-0.1549 - -0.0499) | (0.9905 - 1.1411) | (1.0122 - 1.3594) | (0.9043 - 1.0030) | (0.9654 - 1.0307) |
| | 0.3091 | 0.3473 | 0.0142 | 0.0005 | 0.0901 | 0.0340 | 0.0649 | 0.8820 |
| Race | | | | | | | | |
| Asian | | | | omitted | | | | |
| White | 20.1652*** | 1.0267 | 1.2125** | -0.0761*** | 1.0819** | 1.2404* | 0.9968 | 1.0061 |
| | (9.3521 - 30.9783) | (0.9539 - 1.1050) | (1.0372 - 1.4173) | (-0.1079 - -0.0444) | (1.0027 - 1.1673) | (0.9953 - 1.5458) | (0.9430 - 1.0536) | (0.9702 - 1.0433) |
| | 0.0007 | 0.4830 | 0.0156 | 0.0000 | 0.0424 | 0.0551 | 0.9092 | 0.7438 |
| Native | 161.4648** | 0.7025** | 0.6886** | 0.1401 | 1.0417 | 0.9983 | 0.7425 | 0.9794 |
| | (12.2044 - 310.7253) | (0.5016 - 0.9838) | (0.5086 - 0.9324) | (-0.0478 - 0.3279) | (0.8484 - 1.2790) | (0.6894 - 1.4457) | (0.4677 - 1.1787) | (0.9291 - 1.0324) |

| | | | | | | | | |
|---|---|---|---|---|---|---|---|---|
| | 0.0351 | 0.0399 | 0.0158 | 0.1374 | 0.6964 | 0.9929 | 0.2067 | 0.4390 |
| Brown | 25.2757*** | 1.0721* | 1.2801*** | 0.0262 | 1.0754** | 1.3096** | 1.1533*** | 0.9944 |
| | (14.4817 - 36.0697) | (0.9917 - 1.1590) | (1.1272 - 1.4538) | (-0.0130 - 0.0654) | (1.0083 - 1.1469) | (1.0655 - 1.6097) | (1.0943 - 1.2156) | (0.9565 - 1.0337) |
| | 0.0001 | 0.0803 | 0.0001 | 0.1810 | 0.0269 | 0.0104 | 0.0000 | 0.7760 |
| Black | -2.3177 | 1.2085*** | 1.4672*** | -0.0036 | 1.1479*** | 1.5294*** | 1.3399*** | 0.9829 |
| | (-9.5820 - 4.9467) | (1.1017 - 1.3257) | (1.2243 - 1.7583) | (-0.0488 - 0.0416) | (1.0668 - 1.2353) | (1.2195 - 1.9182) | (1.2679 - 1.4161) | (0.9501 - 1.0169) |
| | 0.5177 | 0.0001 | 0.0000 | 0.8724 | 0.0002 | 0.0002 | 0.0000 | 0.3203 |
| Ignored | 17.7596 | 1.0958 | 1.3845*** | -0.0182 | 1.1036 | 1.4435*** | 1.1215*** | 0.9888 |
| | (-9.1665 - 44.6858) | (0.9303 - 1.2907) | (1.1506 - 1.6659) | (-0.1261 - 0.0897) | (0.9368 - 1.3002) | (1.1234 - 1.8549) | (1.0281 - 1.2235) | (0.9523 - 1.0266) |
| | 0.1868 | 0.2736 | 0.0006 | 0.7316 | 0.2385 | 0.0041 | 0.0098 | 0.5548 |
| City of residence (code) | | | | | | | | |
| Porto Velho (1) | | | | omitted | | | | |
| Rio Branco (2) | -88.8506*** | 1.0871*** | 1.0390 | -0.3806*** | 1.6913*** | 0.6063*** | 0.8006*** | 0.8685*** |
| | (-97.2325 - -80.4687) | (1.0431 - 1.1329) | (0.9626 - 1.1215) | (-0.4130 - -0.3481) | (1.6414 - 1.7427) | (0.5585 - 0.6583) | (0.7669 - 0.8358) | (0.8546 - 0.8826) |
| | 0.0000 | 0.0001 | 0.3260 | 0.0000 | 0.0000 | 0.0000 | 0.0000 | 0.0000 |
| Manaus (3) | -41.1188*** | 1.0145 | 0.5607*** | -0.0444* | 0.9457** | 0.7946*** | 0.9403** | 0.9299*** |
| | (-51.1934 - -31.0443) | (0.9550 - 1.0777) | (0.4871 - 0.6453) | (-0.0961 - 0.0073) | (0.9029 - 0.9905) | (0.6926 - 0.9116) | (0.8865 - 0.9975) | (0.9044 - 0.9561) |
| | 0.0000 | 0.6403 | 0.0000 | 0.0890 | 0.0181 | 0.0010 | 0.0410 | 0.0000 |
| Boa Vista (4) | -41.9559*** | 1.1793*** | 0.5605*** | -0.3627*** | 1.4639*** | 1.1680*** | 1.1467*** | 0.8242*** |
| | (-46.8766 - -37.0352) | (1.1533 - 1.2060) | (0.5396 - 0.5822) | (-0.3787 - -0.3467) | (1.4336 - 1.4949) | (1.1123 - 1.2264) | (1.1293 - 1.1644) | (0.8190 - 0.8295) |
| | 0.0000 | 0.0000 | 0.0000 | 0.0000 | 0.0000 | 0.0000 | 0.0000 | 0.0000 |
| Belem (5) | -131.2887*** | 1.1464*** | 0.8240** | -0.4822*** | 1.2611*** | 0.7014*** | 1.4433*** | 0.9703* |
| | (-142.7246 - -119.8527) | (1.0716 - 1.2264) | (0.7054 - 0.9627) | (-0.5383 - -0.4262) | (1.1969 - 1.3287) | (0.6047 - 0.8135) | (1.3496 - 1.5435) | (0.9409 - 1.0006) |
| | 0.0000 | 0.0001 | 0.0147 | 0.0000 | 0.0000 | 0.0000 | 0.0000 | 0.0549 |
| Macapa (6) | -111.0199*** | 1.6001*** | 1.0277 | -0.0475 | 1.1345*** | 1.2884*** | 1.3012*** | 0.8771*** |
| | (-125.4282 - -96.6115) | (1.4681 - 1.7440) | (0.8581 - 1.2308) | (-0.1119 - 0.0169) | (1.0627 - 1.2112) | (1.0841 - 1.5311) | (1.1972 - 1.4143) | (0.8459 - 0.9094) |
| | 0.0000 | 0.0000 | 0.7662 | 0.1418 | 0.0002 | 0.0040 | 0.0000 | 0.0000 |
| Palmas (7) | -69.0186*** | 0.8638*** | 0.4138*** | 0.0665*** | 0.9078*** | 0.4992*** | 1.5445*** | 0.9407*** |
| | (-72.1883 - -65.8489) | (0.8525 - 0.8753) | (0.4042 - 0.4237) | (0.0546 - 0.0785) | (0.9002 - 0.9155) | (0.4886 - 0.5101) | (1.5329 - 1.5563) | (0.9373 - 0.9441) |
| | 0.0000 | 0.0000 | 0.0000 | 0.0000 | 0.0000 | 0.0000 | 0.0000 | 0.0000 |
| São Luis (8) | -92.8441*** | 1.7465*** | 1.1503 | -0.2999*** | 1.4042*** | 0.8487 | 1.2964*** | 0.9498** |
| | (-110.9532 - -74.7350) | (1.5905 - 1.9178) | (0.9211 - 1.4366) | (-0.3968 - -0.2030) | (1.2830 - 1.5368) | (0.6680 - 1.0782) | (1.1802 - 1.4240) | (0.9052 - 0.9966) |
| | 0.0000 | 0.0000 | 0.2168 | 0.0000 | 0.0000 | 0.1792 | 0.0000 | 0.0359 |

| | | | | | | | | |
|---|---|---|---|---|---|---|---|---|
| Teresina (9) | -80.0210*** | 1.3353*** | 0.7376*** | -0.1906*** | 1.0264** | 0.8860*** | 1.1241*** | 0.8786*** |
| | (-83.9628 - -76.0792) | (1.2961 - 1.3756) | (0.6854 - 0.7939) | (-0.2159 - -0.1652) | (1.0034 - 1.0499) | (0.8242 - 0.9524) | (1.0932 - 1.1559) | (0.8671 - 0.8904) |
| | 0.0000 | 0.0000 | 0.0000 | 0.0000 | 0.0240 | 0.0010 | 0.0000 | 0.0000 |
| Fortaleza (10) | -82.2433*** | 1.2881*** | 0.8801*** | -0.3427*** | 1.0746*** | 0.9883 | 1.0253*** | 0.9175*** |
| | (-86.0238 - -78.4627) | (1.2567 - 1.3203) | (0.8410 - 0.9210) | (-0.3626 - -0.3228) | (1.0481 - 1.1018) | (0.9474 - 1.0310) | (1.0088 - 1.0421) | (0.9100 - 0.9251) |
| | 0.0000 | 0.0000 | 0.0000 | 0.0000 | 0.0000 | 0.5857 | 0.0026 | 0.0000 |
| Natal (11) | -94.5448*** | 1.7077*** | 1.0234 | -0.2013*** | 1.2795*** | 1.4548*** | 1.5814*** | 0.8983*** |
| | (-103.7827 - -85.3069) | (1.6131 - 1.8079) | (0.9064 - 1.1556) | (-0.2467 - -0.1560) | (1.2275 - 1.3337) | (1.3016 - 1.6260) | (1.4934 - 1.6746) | (0.8761 - 0.9211) |
| | 0.0000 | 0.0000 | 0.7083 | 0.0000 | 0.0000 | 0.0000 | 0.0000 | 0.0000 |
| Joao Pessoa (12) | -31.7463*** | 1.1439*** | 0.6701*** | -0.2873*** | 1.2747*** | 1.0236 | 1.0215 | 1.0141 |
| | (-39.1084 - -24.3843) | (1.0895 - 1.2011) | (0.6051 - 0.7421) | (-0.3248 - -0.2499) | (1.2295 - 1.3215) | (0.9336 - 1.1222) | (0.9743 - 1.0710) | (0.9932 - 1.0355) |
| | 0.0000 | 0.0000 | 0.0000 | 0.0000 | 0.0000 | 0.6193 | 0.3776 | 0.1885 |
| Recife (13) | -93.1001*** | 1.3240*** | 1.0764 | -0.3087*** | 1.1291*** | 0.6891*** | 1.0975*** | 0.9627*** |
| | (-99.8688 - -86.3315) | (1.2657 - 1.3850) | (0.9826 - 1.1791) | (-0.3439 - -0.2734) | (1.0948 - 1.1645) | (0.6335 - 0.7496) | (1.0548 - 1.1419) | (0.9461 - 0.9796) |
| | 0.0000 | 0.0000 | 0.1133 | 0.0000 | 0.0000 | 0.0000 | 0.0000 | 0.0000 |
| Maceio (14) | -128.7719*** | 1.3885*** | 0.7786*** | -0.4294*** | 1.3436*** | 0.8278** | 1.2953*** | 0.9807 |
| | (-141.4794 - -116.0644) | (1.2863 - 1.4989) | (0.6539 - 0.9272) | (-0.4929 - -0.3659) | (1.2684 - 1.4232) | (0.7010 - 0.9776) | (1.2033 - 1.3944) | (0.9472 - 1.0153) |
| | 0.0000 | 0.0000 | 0.0050 | 0.0000 | 0.0000 | 0.0260 | 0.0000 | 0.2705 |
| Aracaju (15) | -55.2105*** | 1.3677*** | 1.2169*** | -0.0827*** | 0.7081*** | 1.0586 | 1.1022*** | 0.9098*** |
| | (-65.7889 - -44.6321) | (1.2779 - 1.4638) | (1.0515 - 1.4083) | (-0.1385 - -0.0270) | (0.6713 - 0.7469) | (0.9245 - 1.2122) | (1.0336 - 1.1754) | (0.8827 - 0.9379) |
| | 0.0000 | 0.0000 | 0.0084 | 0.0052 | 0.0000 | 0.4100 | 0.0030 | 0.0000 |
| Salvador (16) | -110.2587*** | 1.7468*** | 1.3150*** | -0.0552*** | 1.0099 | 1.3763*** | 1.3710*** | 0.8929*** |
| | (-114.0133 - -106.5041) | (1.7066 - 1.7879) | (1.2529 - 1.3802) | (-0.0731 - -0.0372) | (0.9934 - 1.0267) | (1.3227 - 1.4321) | (1.3398 - 1.4029) | (0.8844 - 0.9015) |
| | 0.0000 | 0.0000 | 0.0000 | 0.0000 | 0.2390 | 0.0000 | 0.0000 | 0.0000 |
| Belo Horizonte (17) | -169.8290*** | 1.5980*** | 1.0261 | -0.1800*** | 0.9918 | 1.1038*** | 1.5447*** | 0.9155*** |
| | (-176.4361 - -163.2220) | (1.5419 - 1.6561) | (0.9736 - 1.0815) | (-0.2073 - -0.1528) | (0.9642 - 1.0201) | (1.0532 - 1.1570) | (1.5087 - 1.5817) | (0.9051 - 0.9260) |
| | 0.0000 | 0.0000 | 0.3358 | 0.0000 | 0.5660 | 0.0000 | 0.0000 | 0.0000 |
| Vitoria (18) | -83.3557*** | 1.2514*** | 0.7311*** | 0.2001*** | 0.7483*** | 0.7974*** | 1.3501*** | 0.9244*** |
| | (-87.4153 - -79.2961) | (1.2087 - 1.2956) | (0.6877 - 0.7772) | (0.1761 - 0.2242) | (0.7318 - 0.7653) | (0.7576 - 0.8393) | (1.3212 - 1.3795) | (0.9161 - 0.9327) |
| | 0.0000 | 0.0000 | 0.0000 | 0.0000 | 0.0000 | 0.0000 | 0.0000 | 0.0000 |
| Rio de Janeiro (19) | -96.0622*** | 1.3947*** | 0.9093*** | -0.2468*** | 0.9931 | 1.0320* | 1.1883*** | 0.9400*** |
| | (-99.9918 - -92.1327) | (1.3558 - 1.4347) | (0.8715 - 0.9487) | (-0.2661 - -0.2274) | (0.9712 - 1.0155) | (0.9995 - 1.0655) | (1.1722 - 1.2047) | (0.9356 - 0.9443) |
| | 0.0000 | 0.0000 | 0.0000 | 0.0000 | 0.5407 | 0.0535 | 0.0000 | 0.0000 |

| | | | | | | | | |
|---|---|---|---|---|---|---|---|---|
| São Paulo (20) | -133.8293*** | 1.4861*** | 0.9532** | -0.1965*** | 0.9719** | 0.9288*** | 1.3323*** | 0.9071*** |
| | (-139.6387 - -128.0199) | (1.4463 - 1.5271) | (0.9100 - 0.9985) | (-0.2175 - -0.1754) | (0.9489 - 0.9954) | (0.8878 - 0.9717) | (1.3053 - 1.3598) | (0.8983 - 0.9160) |
| | 0.0000 | 0.0000 | 0.0429 | 0.0000 | 0.0195 | 0.0013 | 0.0000 | 0.0000 |
| Curitiba (21) | -96.9031*** | 1.1395*** | 0.8856** | -0.0237 | 0.4916*** | 0.6554*** | 1.0126 | 0.8974*** |
| | (-107.8081 - -85.9980) | (1.0838 - 1.1981) | (0.8016 - 0.9785) | (-0.0654 - 0.0180) | (0.4685 - 0.5159) | (0.5875 - 0.7312) | (0.9655 - 1.0621) | (0.8768 - 0.9184) |
| | 0.0000 | 0.0000 | 0.0170 | 0.2540 | 0.0000 | 0.0000 | 0.6057 | 0.0000 |
| Florianopolis (22) | -27.5872*** | 1.4620*** | 0.8938* | 0.0703*** | 0.9815 | 1.4075*** | 1.1515*** | 0.9001*** |
| | (-38.8118 - -16.3626) | (1.3918 - 1.5358) | (0.7982 - 1.0009) | (0.0266 - 0.1141) | (0.9340 - 1.0314) | (1.2549 - 1.5787) | (1.0951 - 1.2107) | (0.8777 - 0.9230) |
| | 0.0000 | 0.0000 | 0.0518 | 0.0028 | 0.4606 | 0.0000 | 0.0000 | 0.0000 |
| Porto Alegre (23) | -68.4652*** | 1.3445*** | 0.7521*** | -0.0144 | 0.7737*** | 0.7044*** | 1.2917*** | 0.8569*** |
| | (-77.8416 - -59.0888) | (1.2868 - 1.4047) | (0.6815 - 0.8300) | (-0.0523 - 0.0236) | (0.7400 - 0.8088) | (0.6371 - 0.7788) | (1.2381 - 1.3476) | (0.8389 - 0.8754) |
| | 0.0000 | 0.0000 | 0.0000 | 0.4433 | 0.0000 | 0.0000 | 0.0000 | 0.0000 |
| Campo Grande (24) | -99.6117*** | 1.2579*** | 0.9033*** | -0.1393*** | 0.7994*** | 1.0376** | 1.1530*** | 0.9738*** |
| | (-103.8914 - -95.3321) | (1.2218 - 1.2952) | (0.8652 - 0.9431) | (-0.1574 - -0.1211) | (0.7842 - 0.8149) | (1.0064 - 1.0698) | (1.1354 - 1.1709) | (0.9687 - 0.9788) |
| | 0.0000 | 0.0000 | 0.0000 | 0.0000 | 0.0000 | 0.0177 | 0.0000 | 0.0000 |
| Cuiaba (25) | -64.0167*** | 1.2038*** | 0.9428 | -0.2110*** | 0.6046*** | 1.2739*** | 0.8029*** | 0.9486*** |
| | (-72.0186 - -56.0149) | (1.1633 - 1.2456) | (0.8782 - 1.0122) | (-0.2456 - -0.1763) | (0.5857 - 0.6242) | (1.1817 - 1.3733) | (0.7785 - 0.8280) | (0.9302 - 0.9673) |
| | 0.0000 | 0.0000 | 0.1040 | 0.0000 | 0.0000 | 0.0000 | 0.0000 | 0.0000 |
| Goiania (26) | -116.9332*** | 1.2605*** | 0.5413*** | -0.1964*** | 0.9860 | 0.6140*** | 1.1770*** | 0.9796* |
| | (-127.0651 - -106.8012) | (1.2031 - 1.3207) | (0.4946 - 0.5924) | (-0.2390 - -0.1539) | (0.9433 - 1.0306) | (0.5590 - 0.6743) | (1.1251 - 1.2313) | (0.9579 - 1.0017) |
| | 0.0000 | 0.0000 | 0.0000 | 0.0000 | 0.5331 | 0.0000 | 0.0000 | 0.0705 |
| Brasilia (27) | -133.3426*** | 1.5478*** | 0.8031*** | -0.2355*** | 1.0280*** | 0.7640*** | 1.2875*** | 0.9126*** |
| | (-135.8498 - -130.8353) | (1.5171 - 1.5791) | (0.7640 - 0.8443) | (-0.2574 - -0.2137) | (1.0077 - 1.0487) | (0.7267 - 0.8031) | (1.2619 - 1.3136) | (0.9037 - 0.9215) |
| | 0.0000 | 0.0000 | 0.0000 | 0.0000 | 0.0067 | 0.0000 | 0.0000 | 0.0000 |
| Month | | | | | | | | |
| 1 | | | | omitted | | | | |
| 2 | 19.8461*** | 0.8628*** | 0.5206*** | 0.0248** | 1.0159 | 0.6515*** | 0.7146*** | 0.9754 |
| | (12.5218 - 27.1704) | (0.8292 - 0.8978) | (0.4849 - 0.5588) | (0.0016 - 0.0481) | (0.9847 - 1.0482) | (0.6138 - 0.6915) | (0.6809 - 0.7500) | (0.9442 - 1.0076) |
| | 0.0000 | 0.0000 | 0.0000 | 0.0375 | 0.3213 | 0.0000 | 0.0000 | 0.1330 |
| 3 | 4.9881* | 0.8595*** | 0.5877*** | 0.2455*** | 0.9001*** | 0.6390*** | 0.8088*** | 0.9570*** |
| | (-0.2943 - 10.2705) | (0.8316 - 0.8884) | (0.5223 - 0.6613) | (0.2119 - 0.2791) | (0.8695 - 0.9318) | (0.5909 - 0.6910) | (0.7757 - 0.8433) | (0.9406 - 0.9737) |
| | 0.0632 | 0.0000 | 0.0000 | 0.0000 | 0.0000 | 0.0000 | 0.0000 | 0.0000 |
| 4 | -16.9483*** | 1.0908*** | 0.7307*** | 0.0319*** | 1.0399** | 0.6214*** | 1.0088 | 0.9571*** |

| | | | | | | | | |
|---|---|---|---|---|---|---|---|---|
| | (-24.6441 - -9.2526) | (1.0587 - 1.1238) | (0.6605 - 0.8083) | (0.0105 - 0.0533) | (1.0083 - 1.0725) | (0.5741 - 0.6725) | (0.9676 - 1.0516) | (0.9384 - 0.9761) |
| | 0.0001 | 0.0000 | 0.0000 | 0.0050 | 0.0130 | 0.0000 | 0.6811 | 0.0000 |
| 5 | 1.0817 | 0.9196*** | 0.5755*** | 0.1749*** | 0.8826*** | 0.3842*** | 1.0578** | 0.9493*** |
| | (-5.3810 - 7.5444) | (0.8827 - 0.9581) | (0.5282 - 0.6271) | (0.1391 - 0.2107) | (0.8492 - 0.9174) | (0.3470 - 0.4253) | (1.0077 - 1.1104) | (0.9228 - 0.9765) |
| | 0.7336 | 0.0001 | 0.0000 | 0.0000 | 0.0000 | 0.0000 | 0.0231 | 0.0003 |
| 6 | 9.8102** | 0.9928 | 0.6573*** | 0.1704*** | 0.9127*** | 0.6406*** | 0.9489** | 0.9615*** |
| | (1.8188 - 17.8016) | (0.9559 - 1.0311) | (0.5983 - 0.7221) | (0.1267 - 0.2141) | (0.8764 - 0.9504) | (0.5921 - 0.6930) | (0.9059 - 0.9939) | (0.9406 - 0.9828) |
| | 0.0181 | 0.7088 | 0.0000 | 0.0000 | 0.0000 | 0.0000 | 0.0266 | 0.0004 |
| 7 | -3.6094 | 0.9576** | 0.8495*** | 0.1578*** | 0.9830 | 0.6986*** | 0.8631*** | 0.9780* |
| | (-8.5164 - 1.2976) | (0.9182 - 0.9986) | (0.7706 - 0.9365) | (0.1177 - 0.1979) | (0.9454 - 1.0221) | (0.6543 - 0.7459) | (0.8205 - 0.9080) | (0.9554 - 1.0012) |
| | 0.1426 | 0.0430 | 0.0010 | 0.0000 | 0.3881 | 0.0000 | 0.0000 | 0.0626 |
| 8 | 9.1233*** | 0.9618** | 0.6113*** | 0.2168*** | 0.8919*** | 0.5886*** | 1.1229*** | 0.9774** |
| | (4.5849 - 13.6618) | (0.9280 - 0.9968) | (0.5504 - 0.6789) | (0.1857 - 0.2478) | (0.8621 - 0.9228) | (0.5413 - 0.6401) | (1.0605 - 1.1890) | (0.9579 - 0.9974) |
| | 0.0003 | 0.0325 | 0.0000 | 0.0000 | 0.0000 | 0.0000 | 0.0001 | 0.0268 |
| 9 | 10.9309*** | 0.9404** | 0.6560*** | 0.2853*** | 0.7683*** | 0.3676*** | 0.9706 | 0.9725* |
| | (4.9693 - 16.8926) | (0.8907 - 0.9930) | (0.5715 - 0.7531) | (0.2519 - 0.3188) | (0.7357 - 0.8025) | (0.3283 - 0.4116) | (0.9161 - 1.0282) | (0.9430 - 1.0030) |
| | 0.0009 | 0.0268 | 0.0000 | 0.0000 | 0.0000 | 0.0000 | 0.3106 | 0.0771 |
| 10 | -7.0741** | 0.9564 | 0.7446*** | 0.3049*** | 0.8664*** | 0.6609*** | 0.9224** | 0.9673 |
| | (-13.3666 - -0.7815) | (0.8882 - 1.0298) | (0.6358 - 0.8719) | (0.2839 - 0.3259) | (0.8074 - 0.9298) | (0.5581 - 0.7827) | (0.8572 - 0.9927) | (0.9296 - 1.0065) |
| | 0.0290 | 0.2372 | 0.0003 | 0.0000 | 0.0001 | 0.0000 | 0.0310 | 0.1014 |
| 11 | -11.8376* | 1.0828*** | 0.7284*** | 0.2999*** | 1.0189 | 0.6284*** | 1.0099 | 0.9936 |
| | (-23.6853 - 0.0101) | (1.0210 - 1.1484) | (0.6283 - 0.8446) | (0.2704 - 0.3294) | (0.9580 - 1.0837) | (0.5367 - 0.7357) | (0.9396 - 1.0855) | (0.9575 - 1.0310) |
| | 0.0502 | 0.0079 | 0.0000 | 0.0000 | 0.5512 | 0.0000 | 0.7887 | 0.7336 |
| 12 | 6.9636 | 1.0604** | 0.5849*** | 0.4276*** | 0.8904*** | 0.5503*** | 1.0606* | 1.0001 |
| | (-6.9950 - 20.9222) | (1.0113 - 1.1119) | (0.5187 - 0.6596) | (0.3960 - 0.4593) | (0.8404 - 0.9433) | (0.4896 - 0.6185) | (0.9935 - 1.1322) | (0.9689 - 1.0323) |
| | 0.3146 | 0.0153 | 0.0000 | 0.0000 | 0.0001 | 0.0000 | 0.0779 | 0.9941 |
| Year | | | | | | | | |
| 2012 | | | | omitted | | | | |
| 2013 | -18.9071** | 0.9912 | 1.0585 | -0.1984*** | 1.1508*** | 1.6194*** | 0.7706*** | 0.9854 |
| | (-34.0349 - -3.7792) | (0.9389 - 1.0463) | (0.9278 - 1.2077) | (-0.2493 - -0.1475) | (1.0595 - 1.2500) | (1.4411 - 1.8198) | (0.7199 - 0.8248) | (0.9509 - 1.0212) |
| | 0.0163 | 0.7481 | 0.3977 | 0.0000 | 0.0009 | 0.0000 | 0.0000 | 0.4205 |
| 2014 | -40.2014*** | 1.2691*** | 1.3225*** | -0.3042*** | 1.2438*** | 1.3922*** | 0.8004*** | 0.9051*** |
| | (-51.8335 - -28.5692) | (1.2029 - 1.3389) | (1.1890 - 1.4710) | (-0.3459 - -0.2625) | (1.1662 - 1.3265) | (1.2631 - 1.5346) | (0.7552 - 0.8484) | (0.8724 - 0.9389) |

| | | | | | | | | |
|---|---|---|---|---|---|---|---|---|
| | 0.0000 | 0.0000 | 0.0000 | 0.0000 | 0.0000 | 0.0000 | 0.0000 | 0.0000 |
| 2015 | -66.2370*** | 1.5702*** | 1.5891*** | -0.3548*** | 1.3982*** | 1.7274*** | 0.9670 | 0.9101*** |
| | (-83.5910 - -48.8829) | (1.4787 - 1.6673) | (1.4407 - 1.7528) | (-0.4020 - -0.3076) | (1.3199 - 1.4811) | (1.5491 - 1.9263) | (0.9007 - 1.0383) | (0.8775 - 0.9438) |
| | 0.0000 | 0.0000 | 0.0000 | 0.0000 | 0.0000 | 0.0000 | 0.3552 | 0.0000 |
| 2016 | -65.6816*** | 1.5938*** | 1.6750*** | -0.2860*** | 1.2795*** | 1.5347*** | 1.0866*** | 0.9503*** |
| | (-76.9619 - -54.4014) | (1.5144 - 1.6774) | (1.4457 - 1.9407) | (-0.3193 - -0.2527) | (1.2200 - 1.3419) | (1.3362 - 1.7627) | (1.0330 - 1.1429) | (0.9243 - 0.9770) |
| | 0.0000 | 0.0000 | 0.0000 | 0.0000 | 0.0000 | 0.0000 | 0.0013 | 0.0003 |
| Constant | 3,250.8958*** | 0.0574*** | 0.0103*** | 38.4611*** | 0.1118*** | 0.0101*** | 0.0531*** | 1.0429 |
| | (3,222.9482 - 3,278.8433) | (0.0495 - 0.0664) | (0.0086 - 0.0125) | (38.3697 - 38.5525) | (0.0990 - 0.1262) | (0.0082 - 0.0125) | (0.0474 - 0.0596) | (0.9918 - 1.0966) |
| | 0.0000 | 0.0000 | 0.0000 | 0.0000 | 0.0000 | 0.0000 | 0.0000 | 0.1015 |
| Observations | 2,594,223 | 2,594,223 | 2,594,223 | 2,552,977 | 2,555,192 | 2,555,192 | 2,579,539 | 2,593,733 |
| R-squared | 0.0165 | | | 0.0130 | | | | |

| **PANEL B** | (1) | (2) | (3) | (4) | (5) | (6) | (7) | (8) |
|---|---|---|---|---|---|---|---|---|
| | Birthweight | LBW | VLBW | Gestational Age | PTB | VPTB | SGA | Female |
| Unemployment rate months 7 to 9 before birth | -0.6581 | 1.0035 | 1.0099 | -0.0064 | 0.9943 | 1.0100 | 0.9992 | 0.9995 |
| 95% CI | (-2.4943 - 1.1781) | (0.9960 - 1.0110) | (0.9846 - 1.0358) | (-0.0148 - 0.0020) | (0.9831 - 1.0056) | (0.9940 - 1.0261) | (0.9885 - 1.0101) | (0.9949 - 1.0040) |
| p-val | 0.4679 | 0.3597 | 0.4468 | 0.1302 | 0.3215 | 0.2220 | 0.8915 | 0.8196 |
| Unemployment rate months 4 to 6 before birth | 0.3802 | 1.0010 | 0.9817 | -0.0024 | 1.0057 | 0.9962 | 0.9900 | 0.9987 |
| 95% CI | (-1.3624 - 2.1228) | (0.9910 - 1.0111) | (0.9577 - 1.0064) | (-0.0103 - 0.0055) | (0.9963 - 1.0151) | (0.9736 - 1.0192) | (0.9736 - 1.0067) | (0.9920 - 1.0054) |
| p-val | 0.6575 | 0.8489 | 0.1445 | 0.5424 | 0.2337 | 0.7416 | 0.2393 | 0.7059 |
| Unemployment rate months 1 to 3 before birth | -1.7953** | 1.0040 | 1.0268** | -0.0029 | 0.9962 | 1.0265* | 1.0045 | 0.9975 |
| 95% CI | (-3.4172 - -0.1735) | (0.9905 - 1.0177) | (1.0006 - 1.0536) | (-0.0128 - 0.0070) | (0.9851 - 1.0075) | (0.9988 - 1.0549) | (0.9922 - 1.0171) | (0.9916 - 1.0035) |
| p-val | 0.0314 | 0.5650 | 0.0447 | 0.5553 | 0.5126 | 0.0613 | 0.4725 | 0.4169 |
| Maternal characteristics | | | | | | | | |
| Age | | | | | | | | |
| <= 19 | -65.9698*** | 1.0687*** | 0.9850 | -0.0865*** | 1.2025*** | 1.2256*** | 1.0104 | 0.9955 |
| | (-75.4585 - -56.4811) | (1.0354 - 1.1030) | (0.9463 - 1.0253) | (-0.1168 - -0.0562) | (1.1652 - 1.2410) | (1.1845 - 1.2681) | (0.9785 - 1.0433) | (0.9826 - 1.0086) |
| | 0.0000 | 0.0000 | 0.4602 | 0.0000 | 0.0000 | 0.0000 | 0.5268 | 0.4975 |
| 20-24 | -12.6391*** | 0.9242*** | 0.8745*** | 0.0943*** | 0.9664*** | 0.9477** | 0.9985 | 0.9992 |
| | (-18.4537 - -6.8244) | (0.9041 - 0.9447) | (0.8458 - 0.9042) | (0.0739 - 0.1146) | (0.9462 - 0.9870) | (0.9075 - 0.9896) | (0.9855 - 1.0117) | (0.9937 - 1.0047) |
| | 0.0001 | 0.0000 | 0.0000 | 0.0000 | 0.0015 | 0.0151 | 0.8246 | 0.7657 |

| | | | | | | | | |
|---|---|---|---|---|---|---|---|---|
| 25-34 | | | | omitted | | | | |
| >= 35 | -35.9756*** | 1.2862*** | 1.3508*** | -0.2325*** | 1.2537*** | 1.2991*** | 1.1502*** | 1.0043 |
| | (-40.1430 - -31.8082) | (1.2614 - 1.3115) | (1.2780 - 1.4279) | (-0.2500 - -0.2149) | (1.2379 - 1.2697) | (1.2432 - 1.3574) | (1.1294 - 1.1714) | (0.9982 - 1.0104) |
| | 0.0000 | 0.0000 | 0.0000 | 0.0000 | 0.0000 | 0.0000 | 0.0000 | 0.1670 |
| Previous children | | | | | | | | |
| At least 1 alive | 101.4969*** | 0.6775*** | 0.6212*** | 0.0476*** | 0.8898*** | 0.7239*** | 0.6551*** | 1.0035* |
| | (93.2735 - 109.7204) | (0.6578 - 0.6977) | (0.5950 - 0.6486) | (0.0269 - 0.0683) | (0.8720 - 0.9080) | (0.6750 - 0.7763) | (0.6363 - 0.6744) | (0.9999 - 1.0071) |
| | 0.0000 | 0.0000 | 0.0000 | 0.0001 | 0.0000 | 0.0000 | 0.0000 | 0.0553 |
| At least 1 dead | -23.7235*** | 1.2466*** | 1.4792*** | -0.1339*** | 1.2006*** | 1.4261*** | 1.0597*** | 1.0009 |
| | (-29.3855 - -18.0615) | (1.2056 - 1.2889) | (1.4026 - 1.5601) | (-0.1574 - -0.1103) | (1.1656 - 1.2367) | (1.3379 - 1.5202) | (1.0377 - 1.0821) | (0.9946 - 1.0072) |
| | 0.0000 | 0.0000 | 0.0000 | 0.0000 | 0.0000 | 0.0000 | 0.0000 | 0.7788 |
| Education | | | | | | | | |
| None | -112.4277*** | 1.8364*** | 1.7273*** | 0.1203** | 1.3599*** | 1.6353*** | 2.3148*** | 1.0813*** |
| | (-140.6138 - -84.2416) | (1.6745 - 2.0139) | (1.3884 - 2.1490) | (0.0255 - 0.2152) | (1.2353 - 1.4970) | (1.3351 - 2.0030) | (2.1180 - 2.5299) | (1.0439 - 1.1199) |
| | 0.0000 | 0.0000 | 0.0000 | 0.0149 | 0.0000 | 0.0000 | 0.0000 | 0.0000 |
| 1 - 3 years | -51.6449*** | 1.5538*** | 1.3974*** | 0.2035*** | 1.2246*** | 1.3829*** | 2.0081*** | 1.0140 |
| | (-70.4662 - -32.8236) | (1.4686 - 1.6439) | (1.2823 - 1.5229) | (0.1581 - 0.2490) | (1.1671 - 1.2851) | (1.2574 - 1.5210) | (1.8915 - 2.1318) | (0.9935 - 1.0349) |
| | 0.0000 | 0.0000 | 0.0000 | 0.0000 | 0.0000 | 0.0000 | 0.0000 | 0.1821 |
| 4 - 7 years | -27.3873*** | 1.3771*** | 1.2655*** | 0.2057*** | 1.1736*** | 1.2731*** | 1.7320*** | 1.0116** |
| | (-38.6866 - -16.0880) | (1.3176 - 1.4393) | (1.1865 - 1.3498) | (0.1777 - 0.2336) | (1.1429 - 1.2051) | (1.1923 - 1.3593) | (1.6439 - 1.8248) | (1.0013 - 1.0219) |
| | 0.0000 | 0.0000 | 0.0000 | 0.0000 | 0.0000 | 0.0000 | 0.0000 | 0.0269 |
| 8 - 11 years | 9.4751** | 1.1308*** | 1.2048*** | 0.2043*** | 1.0387*** | 1.1658*** | 1.3542*** | 1.0048** |
| | (2.2466 - 16.7036) | (1.1019 - 1.1604) | (1.1466 - 1.2660) | (0.1843 - 0.2243) | (1.0191 - 1.0587) | (1.1132 - 1.2209) | (1.3172 - 1.3923) | (1.0001 - 1.0096) |
| | 0.0122 | 0.0000 | 0.0000 | 0.0000 | 0.0001 | 0.0000 | 0.0000 | 0.0473 |
| 12 years or more | | | | omitted | | | | |
| Ignored | -15.3346 | 1.3564*** | 1.5849*** | 0.1133* | 1.2238*** | 1.5711*** | 1.4361*** | 1.0068 |
| | (-43.2665 - 12.5973) | (1.1752 - 1.5657) | (1.2626 - 1.9894) | (-0.0005 - 0.2271) | (1.0690 - 1.4011) | (1.2959 - 1.9048) | (1.2765 - 1.6156) | (0.9781 - 1.0365) |
| | 0.2694 | 0.0000 | 0.0001 | 0.0509 | 0.0034 | 0.0000 | 0.0000 | 0.6448 |
| Marital status | | | | | | | | |
| Single | | | | omitted | | | | |
| Married | 20.2992*** | 0.8517*** | 0.8178*** | -0.0673*** | 0.9222*** | 0.7681*** | 0.8146*** | 0.9983 |
| | (15.6798 - 24.9186) | (0.8400 - 0.8636) | (0.7875 - 0.8492) | (-0.0883 - -0.0463) | (0.9093 - 0.9354) | (0.7328 - 0.8051) | (0.8032 - 0.8261) | (0.9900 - 1.0067) |
| | 0.0000 | 0.0000 | 0.0000 | 0.0000 | 0.0000 | 0.0000 | 0.0000 | 0.6939 |

| | | | | | | | | |
|---|---|---|---|---|---|---|---|---|
| Widow | 0.9963 | 1.0366 | 0.9811 | -0.0758* | 1.0605 | 0.9782 | 0.9415 | 1.0100 |
| | (-12.0754 - 14.0680) | (0.9400 - 1.1430) | (0.8059 - 1.1943) | (-0.1590 - 0.0073) | (0.9647 - 1.1658) | (0.7733 - 1.2375) | (0.8331 - 1.0640) | (0.9642 - 1.0579) |
| | 0.8767 | 0.4715 | 0.8489 | 0.0722 | 0.2239 | 0.8543 | 0.3338 | 0.6746 |
| Divorced | -1.6090 | 0.9767 | 0.9979 | -0.0907*** | 1.0130 | 0.9656 | 0.8843*** | 0.9828** |
| | (-6.5190 - 3.3010) | (0.9360 - 1.0191) | (0.9165 - 1.0865) | (-0.1191 - -0.0623) | (0.9773 - 1.0499) | (0.8879 - 1.0501) | (0.8555 - 0.9140) | (0.9662 - 0.9998) |
| | 0.5065 | 0.2768 | 0.9606 | 0.0000 | 0.4808 | 0.4133 | 0.0000 | 0.0470 |
| Consensual union | 15.1204*** | 0.9737 | 0.9656 | 0.0424** | 0.9849 | 0.9340 | 0.9672** | 1.0041 |
| | (8.0944 - 22.1465) | (0.9113 - 1.0404) | (0.8663 - 1.0763) | (0.0043 - 0.0805) | (0.9408 - 1.0311) | (0.8584 - 1.0162) | (0.9403 - 0.9948) | (0.9974 - 1.0109) |
| | 0.0002 | 0.4305 | 0.5269 | 0.0306 | 0.5152 | 0.1124 | 0.0202 | 0.2306 |
| Ignored | -6.5246 | 1.0357 | 1.1789** | -0.1024*** | 1.0631* | 1.1729** | 0.9523* | 0.9975 |
| | (-19.4719 - 6.4227) | (0.9626 - 1.1145) | (1.0335 - 1.3447) | (-0.1549 - -0.0500) | (0.9904 - 1.1412) | (1.0120 - 1.3593) | (0.9042 - 1.0030) | (0.9654 - 1.0307) |
| | 0.3098 | 0.3475 | 0.0143 | 0.0005 | 0.0904 | 0.0341 | 0.0646 | 0.8824 |
| Race | | | | | | | | |
| Asian | | | | omitted | | | | |
| White | 20.1599*** | 1.0267 | 1.2125** | -0.0761*** | 1.0819** | 1.2405* | 0.9968 | 1.0061 |
| | (9.3376 - 30.9821) | (0.9539 - 1.1051) | (1.0373 - 1.4173) | (-0.1079 - -0.0443) | (1.0027 - 1.1673) | (0.9954 - 1.5458) | (0.9430 - 1.0537) | (0.9702 - 1.0433) |
| | 0.0007 | 0.4831 | 0.0155 | 0.0000 | 0.0423 | 0.0550 | 0.9101 | 0.7443 |
| Native | 161.4641** | 0.7025** | 0.6886** | 0.1401 | 1.0418 | 0.9983 | 0.7425 | 0.9794 |
| | (12.2003 - 310.7278) | (0.5016 - 0.9838) | (0.5086 - 0.9323) | (-0.0477 - 0.3279) | (0.8484 - 1.2792) | (0.6895 - 1.4456) | (0.4677 - 1.1787) | (0.9291 - 1.0324) |
| | 0.0351 | 0.0399 | 0.0158 | 0.1371 | 0.6959 | 0.9930 | 0.2067 | 0.4390 |
| Brown | 25.2730*** | 1.0720* | 1.2802*** | 0.0262 | 1.0754** | 1.3096** | 1.1533*** | 0.9944 |
| | (14.4726 - 36.0733) | (0.9916 - 1.1590) | (1.1272 - 1.4539) | (-0.0130 - 0.0655) | (1.0084 - 1.1469) | (1.0655 - 1.6096) | (1.0942 - 1.2157) | (0.9565 - 1.0337) |
| | 0.0001 | 0.0803 | 0.0001 | 0.1807 | 0.0268 | 0.0104 | 0.0000 | 0.7757 |
| Black | -2.3230 | 1.2085*** | 1.4673*** | -0.0035 | 1.1480*** | 1.5295*** | 1.3400*** | 0.9829 |
| | (-9.5882 - 4.9423) | (1.1017 - 1.3257) | (1.2244 - 1.7585) | (-0.0488 - 0.0417) | (1.0668 - 1.2353) | (1.2196 - 1.9183) | (1.2679 - 1.4162) | (0.9501 - 1.0169) |
| | 0.5168 | 0.0001 | 0.0000 | 0.8731 | 0.0002 | 0.0002 | 0.0000 | 0.3200 |
| Ignored | 17.7258 | 1.0958 | 1.3853*** | -0.0181 | 1.1036 | 1.4443*** | 1.1217*** | 0.9887 |
| | (-9.2021 - 44.6536) | (0.9302 - 1.2909) | (1.1510 - 1.6674) | (-0.1261 - 0.0898) | (0.9368 - 1.3001) | (1.1241 - 1.8557) | (1.0283 - 1.2237) | (0.9523 - 1.0265) |
| | 0.1877 | 0.2737 | 0.0006 | 0.7327 | 0.2385 | 0.0040 | 0.0097 | 0.5530 |
| City of residence (code) | | | | | | | | |
| Porto Velho (1) | | | | omitted | | | | |
| Rio Branco (2) | -85.8155*** | 1.0838*** | 0.9821 | -0.3841*** | 1.7004*** | 0.5797*** | 0.7867*** | 0.8712*** |
| | (-94.9370 - -76.6941) | (1.0235 - 1.1476) | (0.8946 - 1.0782) | (-0.4242 - -0.3440) | (1.6337 - 1.7698) | (0.5213 - 0.6445) | (0.7497 - 0.8256) | (0.8557 - 0.8870) |

| | | | | | | | | |
|---|---|---|---|---|---|---|---|---|
| | 0.0000 | 0.0058 | 0.7045 | 0.0000 | 0.0000 | 0.0000 | 0.0000 | 0.0000 |
| Manaus (3) | -37.7140*** | 1.0115 | 0.5278*** | -0.0496* | 0.9494* | 0.7540*** | 0.9233** | 0.9336*** |
| | (-48.5033 - -26.9247) | (0.9391 - 1.0893) | (0.4565 - 0.6103) | (-0.1076 - 0.0084) | (0.8951 - 1.0070) | (0.6438 - 0.8830) | (0.8681 - 0.9820) | (0.9091 - 0.9587) |
| | 0.0000 | 0.7634 | 0.0000 | 0.0906 | 0.0838 | 0.0005 | 0.0112 | 0.0000 |
| Boa Vista (4) | -38.9380*** | 1.1768*** | 0.5330*** | -0.3689*** | 1.4646*** | 1.1177*** | 1.1288*** | 0.8275*** |
| | (-46.1165 - -31.7595) | (1.1287 - 1.2269) | (0.4899 - 0.5798) | (-0.3986 - -0.3392) | (1.4031 - 1.5287) | (1.0270 - 1.2163) | (1.0930 - 1.1658) | (0.8139 - 0.8414) |
| | 0.0000 | 0.0000 | 0.0000 | 0.0000 | 0.0000 | 0.0100 | 0.0000 | 0.0000 |
| Belem (5) | -129.8171*** | 1.1471*** | 0.8115** | -0.4918*** | 1.2465*** | 0.6833*** | 1.4381*** | 0.9745* |
| | (-142.6011 - -117.0331) | (1.0579 - 1.2439) | (0.6868 - 0.9590) | (-0.5528 - -0.4309) | (1.1682 - 1.3300) | (0.5842 - 0.7992) | (1.3318 - 1.5530) | (0.9450 - 1.0048) |
| | 0.0000 | 0.0009 | 0.0142 | 0.0000 | 0.0000 | 0.0000 | 0.0000 | 0.0982 |
| Macapa (6) | -109.4265*** | 1.5996*** | 1.0072 | -0.0539 | 1.1278*** | 1.2552** | 1.2939*** | 0.8799*** |
| | (-124.1471 - -94.7059) | (1.4559 - 1.7575) | (0.8444 - 1.2014) | (-0.1206 - 0.0127) | (1.0487 - 1.2128) | (1.0535 - 1.4956) | (1.1873 - 1.4102) | (0.8498 - 0.9111) |
| | 0.0000 | 0.0000 | 0.9362 | 0.1084 | 0.0012 | 0.0110 | 0.0000 | 0.0000 |
| Palmas (7) | -64.8279*** | 0.8615*** | 0.3883*** | 0.0566*** | 0.9061*** | 0.4680*** | 1.5133*** | 0.9464*** |
| | (-73.3639 - -56.2920) | (0.8158 - 0.9097) | (0.3437 - 0.4387) | (0.0184 - 0.0949) | (0.8566 - 0.9584) | (0.4212 - 0.5200) | (1.4429 - 1.5871) | (0.9239 - 0.9694) |
| | 0.0000 | 0.0000 | 0.0000 | 0.0053 | 0.0006 | 0.0000 | 0.0000 | 0.0000 |
| São Luis (8) | -89.8193*** | 1.7429*** | 1.0987 | -0.3071*** | 1.4018*** | 0.8116 | 1.2777*** | 0.9540** |
| | (-107.6851 - -71.9535) | (1.5599 - 1.9474) | (0.8778 - 1.3752) | (-0.4097 - -0.2045) | (1.2767 - 1.5392) | (0.6319 - 1.0425) | (1.1620 - 1.4049) | (0.9114 - 0.9986) |
| | 0.0000 | 0.0000 | 0.4111 | 0.0000 | 0.0000 | 0.1022 | 0.0000 | 0.0433 |
| Teresina (9) | -76.0544*** | 1.3305*** | 0.6885*** | -0.1962*** | 1.0319 | 0.8367*** | 1.0993*** | 0.8825*** |
| | (-82.1546 - -69.9542) | (1.2579 - 1.4072) | (0.6158 - 0.7697) | (-0.2339 - -0.1585) | (0.9873 - 1.0784) | (0.7492 - 0.9343) | (1.0532 - 1.1475) | (0.8652 - 0.9001) |
| | 0.0000 | 0.0000 | 0.0000 | 0.0000 | 0.1635 | 0.0015 | 0.0000 | 0.0000 |
| Fortaleza (10) | -80.3573*** | 1.2878*** | 0.8581*** | -0.3507*** | 1.0667*** | 0.9596 | 1.0179 | 0.9212*** |
| | (-86.4334 - -74.2811) | (1.2328 - 1.3454) | (0.7752 - 0.9497) | (-0.3844 - -0.3170) | (1.0228 - 1.1125) | (0.8902 - 1.0345) | (0.9819 - 1.0552) | (0.9080 - 0.9346) |
| | 0.0000 | 0.0000 | 0.0031 | 0.0000 | 0.0026 | 0.2823 | 0.3344 | 0.0000 |
| Natal (11) | -91.0894*** | 1.7034*** | 0.9671 | -0.2085*** | 1.2800*** | 1.3828*** | 1.5528*** | 0.9025*** |
| | (-101.6254 - -80.5534) | (1.5806 - 1.8358) | (0.8423 - 1.1104) | (-0.2623 - -0.1547) | (1.2059 - 1.3587) | (1.2100 - 1.5804) | (1.4566 - 1.6554) | (0.8799 - 0.9256) |
| | 0.0000 | 0.0000 | 0.6354 | 0.0000 | 0.0000 | 0.0000 | 0.0000 | 0.0000 |
| Joao Pessoa (12) | -29.5242*** | 1.1424*** | 0.6453*** | -0.2933*** | 1.2719*** | 0.9867 | 1.0106 | 1.0176* |
| | (-37.9256 - -21.1229) | (1.0741 - 1.2150) | (0.5754 - 0.7236) | (-0.3356 - -0.2511) | (1.2118 - 1.3350) | (0.8846 - 1.1006) | (0.9597 - 1.0641) | (0.9971 - 1.0386) |
| | 0.0000 | 0.0000 | 0.0000 | 0.0000 | 0.0000 | 0.8106 | 0.6896 | 0.0932 |
| Recife (13) | -89.2320*** | 1.3200*** | 1.0071 | -0.3161*** | 1.1308*** | 0.6511*** | 1.0752*** | 0.9675*** |
| | (-98.0993 - -80.3648) | (1.2343 - 1.4117) | (0.8890 - 1.1409) | (-0.3636 - -0.2686) | (1.0701 - 1.1949) | (0.5780 - 0.7335) | (1.0209 - 1.1323) | (0.9475 - 0.9880) |

| | | | | | | | | |
|---|---|---|---|---|---|---|---|---|
| | 0.0000 | 0.0000 | 0.9114 | 0.0000 | 0.0000 | 0.0000 | 0.0061 | 0.0020 |
| Maceio (14) | -123.6689*** | 1.3826*** | 0.7139*** | -0.4379*** | 1.3492*** | 0.7684*** | 1.2601*** | 0.9867 |
| | (-137.6409 - -109.6968) | (1.2499 - 1.5294) | (0.5881 - 0.8665) | (-0.5139 - -0.3619) | (1.2449 - 1.4621) | (0.6296 - 0.9378) | (1.1623 - 1.3661) | (0.9528 - 1.0218) |
| | 0.0000 | 0.0000 | 0.0006 | 0.0000 | 0.0000 | 0.0096 | 0.0000 | 0.4524 |
| Aracaju (15) | -52.4394*** | 1.3652*** | 1.1626* | -0.0894*** | 0.7071*** | 1.0122 | 1.0872** | 0.9135*** |
| | (-63.7928 - -41.0861) | (1.2584 - 1.4811) | (0.9959 - 1.3572) | (-0.1502 - -0.0285) | (0.6631 - 0.7541) | (0.8696 - 1.1781) | (1.0167 - 1.1626) | (0.8872 - 0.9405) |
| | 0.0000 | 0.0000 | 0.0564 | 0.0056 | 0.0000 | 0.8759 | 0.0146 | 0.0000 |
| Salvador (16) | -108.9157*** | 1.7464*** | 1.2891*** | -0.0609*** | 1.0049 | 1.3442*** | 1.3639*** | 0.8954*** |
| | (-114.5995 - -103.2320) | (1.6844 - 1.8108) | (1.1940 - 1.3918) | (-0.0859 - -0.0359) | (0.9737 - 1.0371) | (1.2699 - 1.4228) | (1.3183 - 1.4110) | (0.8858 - 0.9052) |
| | 0.0000 | 0.0000 | 0.0000 | 0.0000 | 0.7602 | 0.0000 | 0.0000 | 0.0000 |
| Belo Horizonte (17) | -168.7740*** | 1.5990*** | 1.0157 | -0.1869*** | 0.9833 | 1.0851*** | 1.5404*** | 0.9183*** |
| | (-177.3531 - -160.1949) | (1.5369 - 1.6636) | (0.9268 - 1.1132) | (-0.2211 - -0.1527) | (0.9407 - 1.0279) | (1.0212 - 1.1529) | (1.4906 - 1.5920) | (0.9028 - 0.9340) |
| | 0.0000 | 0.0000 | 0.7384 | 0.0000 | 0.4577 | 0.0084 | 0.0000 | 0.0000 |
| Vitoria (18) | -80.9149*** | 1.2492*** | 0.7019*** | 0.1950*** | 0.7481*** | 0.7683*** | 1.3340*** | 0.9274*** |
| | (-86.6593 - -75.1706) | (1.1927 - 1.3085) | (0.6468 - 0.7618) | (0.1640 - 0.2260) | (0.7216 - 0.7756) | (0.7119 - 0.8293) | (1.2925 - 1.3767) | (0.9158 - 0.9392) |
| | 0.0000 | 0.0000 | 0.0000 | 0.0000 | 0.0000 | 0.0000 | 0.0000 | 0.0000 |
| Rio de Janeiro (19) | -94.5331*** | 1.3935*** | 0.8873*** | -0.2507*** | 0.9918 | 1.0084 | 1.1794*** | 0.9421*** |
| | (-99.6388 - -89.4274) | (1.3464 - 1.4423) | (0.8339 - 0.9441) | (-0.2744 - -0.2270) | (0.9619 - 1.0226) | (0.9640 - 1.0548) | (1.1518 - 1.2076) | (0.9340 - 0.9503) |
| | 0.0000 | 0.0000 | 0.0002 | 0.0000 | 0.5977 | 0.7161 | 0.0000 | 0.0000 |
| São Paulo (20) | -133.0406*** | 1.4862*** | 0.9436* | -0.2001*** | 0.9684** | 0.9171*** | 1.3282*** | 0.9087*** |
| | (-139.7099 - -126.3713) | (1.4436 - 1.5300) | (0.8852 - 1.0058) | (-0.2241 - -0.1761) | (0.9385 - 0.9992) | (0.8734 - 0.9630) | (1.2958 - 1.3613) | (0.8972 - 0.9204) |
| | 0.0000 | 0.0000 | 0.0747 | 0.0000 | 0.0447 | 0.0005 | 0.0000 | 0.0000 |
| Curitiba (21) | -96.9286*** | 1.1414*** | 0.8943* | -0.0292 | 0.4866*** | 0.6547*** | 1.0159 | 0.8991*** |
| | (-108.6107 - -85.2465) | (1.0877 - 1.1979) | (0.7970 - 1.0035) | (-0.0753 - 0.0170) | (0.4595 - 0.5153) | (0.5865 - 0.7308) | (0.9680 - 1.0663) | (0.8760 - 0.9228) |
| | 0.0000 | 0.0000 | 0.0574 | 0.2056 | 0.0000 | 0.0000 | 0.5216 | 0.0000 |
| Florianopolis (22) | -27.4455*** | 1.4638*** | 0.8971* | 0.0658*** | 0.9740 | 1.4030*** | 1.1536*** | 0.9016*** |
| | (-39.0367 - -15.8543) | (1.3957 - 1.5353) | (0.7936 - 1.0140) | (0.0190 - 0.1126) | (0.9211 - 1.0300) | (1.2520 - 1.5723) | (1.0958 - 1.2144) | (0.8772 - 0.9266) |
| | 0.0000 | 0.0000 | 0.0823 | 0.0077 | 0.3551 | 0.0000 | 0.0000 | 0.0000 |
| Porto Alegre (23) | -68.5508*** | 1.3471*** | 0.7620*** | -0.0207 | 0.7645*** | 0.7050*** | 1.2975*** | 0.8588*** |
| | (-79.0111 - -58.0905) | (1.2901 - 1.4067) | (0.6767 - 0.8580) | (-0.0645 - 0.0232) | (0.7229 - 0.8085) | (0.6360 - 0.7815) | (1.2385 - 1.3593) | (0.8378 - 0.8803) |
| | 0.0000 | 0.0000 | 0.0000 | 0.3414 | 0.0000 | 0.0000 | 0.0000 | 0.0000 |
| Campo Grande (24) | -99.5663*** | 1.2573*** | 0.8971*** | -0.1378*** | 0.8015*** | 1.0344** | 1.1516*** | 0.9733*** |
| | (-103.7838 - -95.3488) | (1.2204 - 1.2954) | (0.8560 - 0.9402) | (-0.1561 - -0.1194) | (0.7862 - 0.8171) | (1.0010 - 1.0688) | (1.1316 - 1.1719) | (0.9681 - 0.9785) |

| | | | | | | | | |
|---|---|---|---|---|---|---|---|---|
| | 0.0000 | 0.0000 | 0.0000 | 0.0000 | 0.0000 | 0.0431 | 0.0000 | 0.0000 |
| Cuiaba (25) | -62.6706*** | 1.2049*** | 0.9357 | -0.2206*** | 0.5973*** | 1.2496*** | 0.8007*** | 0.9526*** |
| | (-73.2917 - -52.0495) | (1.1539 - 1.2581) | (0.8288 - 1.0564) | (-0.2653 - -0.1759) | (0.5644 - 0.6321) | (1.1450 - 1.3637) | (0.7624 - 0.8410) | (0.9269 - 0.9789) |
| | 0.0000 | 0.0000 | 0.2832 | 0.0000 | 0.0000 | 0.0000 | 0.0000 | 0.0005 |
| Goiania (26) | -116.9060*** | 1.2624*** | 0.5454*** | -0.2015*** | 0.9771 | 0.6126*** | 1.1801*** | 0.9813 |
| | (-127.5393 - -106.2728) | (1.2071 - 1.3202) | (0.4908 - 0.6061) | (-0.2481 - -0.1550) | (0.9278 - 1.0289) | (0.5567 - 0.6740) | (1.1267 - 1.2361) | (0.9573 - 1.0059) |
| | 0.0000 | 0.0000 | 0.0000 | 0.0000 | 0.3790 | 0.0000 | 0.0000 | 0.1357 |
| Brasilia (27) | -130.8097*** | 1.5458*** | 0.7728*** | -0.2428*** | 1.0244 | 0.7356*** | 1.2722*** | 0.9163*** |
| | (-136.2784 - -125.3410) | (1.4796 - 1.6150) | (0.7022 - 0.8504) | (-0.2767 - -0.2089) | (0.9851 - 1.0652) | (0.6792 - 0.7967) | (1.2265 - 1.3195) | (0.9023 - 0.9306) |
| | 0.0000 | 0.0000 | 0.0000 | 0.0000 | 0.2267 | 0.0000 | 0.0000 | 0.0000 |
| | 25.1866*** | 0.8623*** | 0.5165*** | 0.0290* | 1.0177 | 0.6477*** | 0.7127*** | 0.9756 |
| Month | | | | | | | | |
| 1 | | | | omitted | | | | |
| 2 | 19.7141*** | 0.8623*** | 0.5165*** | 0.0248** | 1.0177 | 0.6477*** | 0.7127*** | 0.9756 |
| | (12.4056 - 27.0225) | (0.8289 - 0.8971) | (0.4814 - 0.5541) | (0.0012 - 0.0483) | (0.9871 - 1.0492) | (0.6093 - 0.6886) | (0.6792 - 0.7479) | (0.9445 - 1.0079) |
| | 0.0000 | 0.0000 | 0.0000 | 0.0398 | 0.2601 | 0.0000 | 0.0000 | 0.1369 |
| 3 | 4.8260* | 0.8585*** | 0.5771*** | 0.2452*** | 0.9034*** | 0.6304*** | 0.8040*** | 0.9575*** |
| | (-0.4380 - 10.0899) | (0.8307 - 0.8872) | (0.5154 - 0.6462) | (0.2125 - 0.2780) | (0.8742 - 0.9335) | (0.5848 - 0.6795) | (0.7740 - 0.8352) | (0.9403 - 0.9751) |
| | 0.0707 | 0.0000 | 0.0000 | 0.0000 | 0.0000 | 0.0000 | 0.0000 | 0.0000 |
| 4 | -7.0336*** | 1.0887*** | 0.7098*** | 0.0314*** | 1.0459*** | 0.6085*** | 0.9996 | 0.9579*** |
| | (-11.9054 - -2.1618) | (1.0549 - 1.1236) | (0.6424 - 0.7844) | (0.0098 - 0.0530) | (1.0151 - 1.0776) | (0.5593 - 0.6620) | (0.9591 - 1.0418) | (0.9392 - 0.9770) |
| | 0.0064 | 0.0000 | 0.0000 | 0.0060 | 0.0033 | 0.0000 | 0.9842 | 0.0000 |
| 5 | 2.3445 | 0.9181*** | 0.5600*** | 0.1743*** | 0.8863*** | 0.3766*** | 1.0492* | 0.9502*** |
| | (-4.6872 - 9.3762) | (0.8788 - 0.9592) | (0.5159 - 0.6080) | (0.1391 - 0.2095) | (0.8537 - 0.9201) | (0.3372 - 0.4206) | (0.9992 - 1.1017) | (0.9237 - 0.9775) |
| | 0.4992 | 0.0001 | 0.0000 | 0.0000 | 0.0000 | 0.0000 | 0.0539 | 0.0004 |
| 6 | 10.0363** | 0.9916 | 0.6418*** | 0.1437*** | 0.9144*** | 0.6294*** | 0.9426*** | 0.9627*** |
| | (2.4349 - 17.6377) | (0.9555 - 1.0290) | (0.5853 - 0.7038) | (0.1138 - 0.1737) | (0.8756 - 0.9549) | (0.5823 - 0.6804) | (0.9019 - 0.9851) | (0.9424 - 0.9833) |
| | 0.0116 | 0.6538 | 0.0000 | 0.0000 | 0.0001 | 0.0000 | 0.0086 | 0.0004 |
| 7 | 0.1321 | 0.9568* | 0.8321*** | 0.1572*** | 0.9827 | 0.6881*** | 0.8587*** | 0.9794* |
| | (-5.9595 - 6.2238) | (0.9130 - 1.0027) | (0.7560 - 0.9157) | (0.1179 - 0.1965) | (0.9422 - 1.0249) | (0.6467 - 0.7321) | (0.8158 - 0.9038) | (0.9571 - 1.0022) |
| | 0.9648 | 0.0648 | 0.0002 | 0.0000 | 0.4164 | 0.0000 | 0.0000 | 0.0762 |
| 8 | -9.1424** | 0.9604** | 0.5939*** | 0.2078*** | 0.8935*** | 0.5754*** | 1.1137*** | 0.9790** |
| | (-16.9163 - -1.3684) | (0.9231 - 0.9992) | (0.5341 - 0.6604) | (0.1782 - 0.2375) | (0.8621 - 0.9260) | (0.5274 - 0.6277) | (1.0541 - 1.1766) | (0.9589 - 0.9996) |

| | | | | | | | | |
|---|---|---|---|---|---|---|---|---|
| | 0.0229 | 0.0456 | 0.0000 | 0.0000 | 0.0000 | 0.0000 | 0.0001 | 0.0457 |
| 9 | 13.2971*** | 0.9386** | 0.6327*** | 0.2843*** | 0.7711*** | 0.3571*** | 0.9600 | 0.9743* |
| | (6.4917 - 20.1026) | (0.8868 - 0.9934) | (0.5531 - 0.7239) | (0.2506 - 0.3180) | (0.7339 - 0.8101) | (0.3204 - 0.3980) | (0.9079 - 1.0152) | (0.9453 - 1.0042) |
| | 0.0004 | 0.0285 | 0.0000 | 0.0000 | 0.0000 | 0.0000 | 0.1526 | 0.0919 |
| 10 | 6.0083 | 0.9543 | 0.7156*** | 0.2587*** | 0.8699*** | 0.6390*** | 0.9110*** | 0.9694 |
| | (-8.5062 - 20.5227) | (0.8912 - 1.0219) | (0.6194 - 0.8268) | (0.1944 - 0.3231) | (0.8067 - 0.9380) | (0.5347 - 0.7637) | (0.8488 - 0.9779) | (0.9328 - 1.0074) |
| | 0.4026 | 0.1801 | 0.0000 | 0.0000 | 0.0003 | 0.0000 | 0.0099 | 0.1133 |
| 11 | 1.2740 | 1.0805*** | 0.6999*** | 0.2535*** | 1.0228 | 0.6070*** | 0.9972 | 0.9958 |
| | (-11.2021 - 13.7501) | (1.0208 - 1.1437) | (0.6070 - 0.8070) | (0.1797 - 0.3273) | (0.9580 - 1.0920) | (0.5152 - 0.7151) | (0.9286 - 1.0709) | (0.9630 - 1.0297) |
| | 0.8354 | 0.0076 | 0.0000 | 0.0000 | 0.4999 | 0.0000 | 0.9386 | 0.8066 |
| 12 | 20.0984*** | 1.0580** | 0.5617*** | 0.3812*** | 0.8938*** | 0.5314*** | 1.0469 | 1.0024 |
| | (11.9669 - 28.2299) | (1.0111 - 1.1072) | (0.5041 - 0.6258) | (0.3195 - 0.4429) | (0.8405 - 0.9505) | (0.4667 - 0.6052) | (0.9823 - 1.1157) | (0.9734 - 1.0323) |
| | 0.0000 | 0.0149 | 0.0000 | 0.0000 | 0.0003 | 0.0000 | 0.1582 | 0.8720 |
| Year | | | | | | | | |
| 2012 | | | | omitted | | | | |
| 2013 | -5.1088 | 0.9886 | 1.0070 | -0.2461*** | 1.1551*** | 1.5521*** | 0.7581*** | 0.9885 |
| | (-13.7436 - 3.5259) | (0.9423 - 1.0371) | (0.9024 - 1.1237) | (-0.2975 - -0.1948) | (1.0601 - 1.2588) | (1.3688 - 1.7599) | (0.7071 - 0.8129) | (0.9528 - 1.0256) |
| | 0.2348 | 0.6392 | 0.9010 | 0.0000 | 0.0010 | 0.0000 | 0.0000 | 0.5386 |
| 2014 | -27.1678*** | 1.2677*** | 1.2792*** | -0.3537*** | 1.2407*** | 1.3458*** | 0.7925*** | 0.9080*** |
| | (-43.8199 - -10.5157) | (1.1997 - 1.3395) | (1.1554 - 1.4163) | (-0.4474 - -0.2600) | (1.1519 - 1.3364) | (1.2066 - 1.5009) | (0.7434 - 0.8448) | (0.8767 - 0.9404) |
| | 0.0025 | 0.0000 | 0.0000 | 0.0000 | 0.0000 | 0.0000 | 0.0000 | 0.0000 |
| 2015 | -52.3934*** | 1.5672*** | 1.5173*** | -0.4052*** | 1.3972*** | 1.6567*** | 0.9526 | 0.9138*** |
| | (-67.8548 - -36.9319) | (1.4915 - 1.6468) | (1.3616 - 1.6908) | (-0.4998 - -0.3106) | (1.2998 - 1.5018) | (1.4688 - 1.8686) | (0.8888 - 1.0209) | (0.8818 - 0.9470) |
| | 0.0000 | 0.0000 | 0.0000 | 0.0000 | 0.0000 | 0.0000 | 0.1693 | 0.0000 |
| 2016 | -50.3103*** | 1.5895*** | 1.5619*** | -0.3404*** | 1.2769*** | 1.4376*** | 1.0627* | 0.9564*** |
| | (-66.8694 - -33.7511) | (1.4848 - 1.7015) | (1.3145 - 1.8557) | (-0.4303 - -0.2504) | (1.1846 - 1.3764) | (1.2051 - 1.7149) | (0.9934 - 1.1369) | (0.9324 - 0.9811) |
| | 0.0000 | 0.0000 | 0.0000 | 0.0000 | 0.0000 | 0.0001 | 0.0772 | 0.0006 |
| Constant | 3,239.1865*** | 0.0573*** | 0.0104*** | 38.5099*** | 0.1125*** | 0.0102*** | 0.0533*** | 1.0405 |
| | (3,213.8189 - 3,264.5542) | (0.0498 - 0.0660) | (0.0086 - 0.0126) | (38.3988 - 38.6210) | (0.0993 - 0.1273) | (0.0082 - 0.0127) | (0.0477 - 0.0596) | (0.9887 - 1.0951) |
| | 0.0000 | 0.0000 | 0.0000 | 0.0000 | 0.0000 | 0.0000 | 0.0000 | 0.1274 |
| Observations | 2,594,223 | 2,594,223 | 2,594,223 | 2,552,977 | 2,555,192 | 2,555,192 | 2,579,539 | 2,593,733 |

| | | |
|---|---|---|
| R-squared | 0.0165 | 0.0130 |

Note: coefficients of interaction terms are not reported. *** p<0.01, ** p<0.05, * p<0.1
