# Supplementary material for: In utero exposure to economic fluctuations and birth outcomes: An analysis of the relevance of the local unemployment rate in Brazilian state capitals
Source: PLoS One. 2019 Oct 10;14(10):e0223673. doi: 10.1371/journal.pone.0223673 (PMC6786569; doi:10.1371/journal.pone.0223673)
Supplement: S2 Table — (PDF) [file pone.0223673.s002.pdf]

S2 Table. Extended version of table 3.

	Ignored as missing			Same as original			Without controlling for race		
	(1)	(2)	(3)	(4)	(5)	(6)	(7)	(8)	(9)
	Birthweight	LBW	VLBW	Birthweight	LBW	VLBW	Birthweight	LBW	VLBW
Unemployment rate months 7 to 9 before birth	-0.5242	1.0015	1.0108	-0.5014	1.0019	1.0083	-0.6140	1.0034	1.0100
95% CI	(-2.4335 - 1.3851)	(0.9943 - 1.0088)	(0.9858 - 1.0365)	(-2.4040 - 1.4012)	(0.9948 - 1.0091)	(0.9835 - 1.0338)	(-2.4610 - 1.2331)	(0.9959 - 1.0110)	(0.9846 - 1.0360)
p-val	0.5774	0.6827	0.4004	0.5927	0.5987	0.5140	0.5005	0.3714	0.4437
Unemployment rate months 4 to 6 before birth	0.5697	0.9992	0.9822	0.4524	0.9994	0.9840	0.3750	1.0010	0.9818
95% CI	(-1.0923 - 2.2317)	(0.9895 - 1.0090)	(0.9566 - 1.0085)	(-1.2511 - 2.1559)	(0.9897 - 1.0092)	(0.9586 - 1.0100)	(-1.3577 - 2.1077)	(0.9911 - 1.0109)	(0.9578 - 1.0064)
p-val	0.4873	0.8746	0.1821	0.5898	0.9084	0.2258	0.6601	0.8443	0.1451
Unemployment rate months 1 to 3 before birth	-1.6919*	1.0055	1.0268**	-1.7921**	1.0061	1.0278**	-1.7360**	1.0038	1.0263**
95% CI	(-3.4614 - 0.0776)	(0.9930 - 1.0182)	(1.0016 - 1.0528)	(-3.5706 - -0.0137)	(0.9936 - 1.0188)	(1.0030 - 1.0532)	(-3.3702 - -0.1018)	(0.9903 - 1.0175)	(1.0001 - 1.0532)
p-val	0.0601	0.3909	0.0372	0.0484	0.3420	0.0278	0.0382	0.5806	0.0493
Maternal characteristics									
Age									
<= 19	-65.4387***	1.0666***	0.9765	-65.5472***	1.0666***	0.9757	-65.5310***	1.0685***	0.9850
	(-75.2999 - -55.5775)	(1.0313 - 1.1031)	(0.9319 - 1.0232)	(-75.3397 - -55.7548)	(1.0315 - 1.1029)	(0.9316 - 1.0220)	(-74.9681 - -56.0939)	(1.0355 - 1.1025)	(0.9469 - 1.0245)
	0.0000	0.0002	0.3190	0.0000	0.0002	0.2989	0.0000	0.0000	0.4509
20-24	-12.5250***	0.9233***	0.8678***	-12.5072***	0.9236***	0.8665***	-12.2997***	0.9245***	0.8748***
	(-18.4263 - -6.6237)	(0.9019 - 0.9452)	(0.8393 - 0.8974)	(-18.4548 - -6.5596)	(0.9023 - 0.9454)	(0.8381 - 0.8958)	(-17.9510 - -6.6484)	(0.9053 - 0.9440)	(0.8458 - 0.9048)
	0.0002	0.0000	0.0000	0.0002	0.0000	0.0000	0.0001	0.0000	0.0000
25-34					omitted				
>= 35	-35.8294***	1.2852***	1.3510***	-35.9986***	1.2859***	1.3513***	-36.1468***	1.2840***	1.3478***
	(-39.9348 - -31.7240)	(1.2603 - 1.3107)	(1.2760 - 1.4305)	(-40.1180 - -31.8792)	(1.2608 - 1.3116)	(1.2793 - 1.4273)	(-40.0811 - -32.2124)	(1.2601 - 1.3084)	(1.2762 - 1.4235)
	0.0000	0.0000	0.0000	0.0000	0.0000	0.0000	0.0000	0.0000	0.0000
Previous children									
At least 1 alive	101.5209***	0.6767***	0.6189***	101.5066***	0.6774***	0.6198***	101.6956***	0.6786***	0.6225***
	(92.9438 - 110.0980)	(0.6562 - 0.6978)	(0.5940 - 0.6449)	(92.9793 - 110.0339)	(0.6572 - 0.6982)	(0.5946 - 0.6461)	(93.6087 - 109.7824)	(0.6589 - 0.6990)	(0.5954 - 0.6508)
	0.0000	0.0000	0.0000	0.0000	0.0000	0.0000	0.0000	0.0000	0.0000
At least 1 dead	-23.4993***	1.2460***	1.4815***	-23.4976***	1.2457***	1.4809***	-23.8175***	1.2482***	1.4817***
	(-29.3082 - -17.6904)	(1.2044 - 1.2889)	(1.4047 - 1.5625)	(-29.3459 - -17.6493)	(1.2039 - 1.2891)	(1.4035 - 1.5625)	(-29.5071 - -18.1280)	(1.2076 - 1.2901)	(1.4059 - 1.5616)
	0.0000	0.0000	0.0000	0.0000	0.0000	0.0000	0.0000	0.0000	0.0000
Education									
None	-112.3452***	1.8389***	1.7014***	-34.0333	1.5859***	1.9083***	-14.7532	1.3783***	1.6172***
	(-140.2776 - -84.4127)	(1.6863 - 2.0053)	(1.3597 - 2.1290)	(-77.5545 - 9.4879)	(1.2824 - 1.9612)	(1.3615 - 2.6748)	(-42.7287 - 13.2223)	(1.1944 - 1.5906)	(1.2901 - 2.0273)
	0.0000	0.0000	0.0000	0.1200	0.0000	0.0002	0.2883	0.0000	0.0000

1 - 3 years	-50.8781***	1.5444***	1.4060***	-50.6273***	1.5424***	1.3975***	-50.4321***	1.5834***	1.4318***
	(-69.7827 - -31.9735)	(1.4633 - 1.6301)	(1.2934 - 1.5283	(-69.4322 - -31.8224)	(1.4617 - 1.6276)	(1.2807 - 1.5249)	(-69.9715 - -30.8927)	(1.4910 - 1.6816)	(1.3124 - 1.5622)
	0.0000	0.0000	0.0000	0.0000	0.0000	0.0000	0.0000	0.0000	0.0000
4 - 7 years	-27.4722***	1.3787***	1.2643***	-27.1976***	1.3760***	1.2593***	-26.3599***	1.4020***	1.2948***
	(-38.6659 - -16.2784)	(1.3202 - 1.4399)	(1.1782 - 1.3567	(-38.4715 - -15.9238)	(1.3166 - 1.4380)	(1.1725 - 1.3527)	(-37.7498 - -14.9700)	(1.3414 - 1.4653)	(1.2164 - 1.3783)
	0.0000	0.0000	0.0000	0.0000	0.0000	0.0000	0.0001	0.0000	0.0000
8 - 11 years	9.3537**	1.1316***	1.2157***	9.5522**	1.1302***	1.2110***	10.2917***	1.1479***	1.2282***
	(2.0059 - 16.7015)	(1.1012 - 1.1627)	(1.1571 - 1.2772	(2.1327 - 16.9716)	(1.0995 - 1.1618)	(1.1515 - 1.2737)	(3.0929 - 17.4905)	(1.1164 - 1.1802)	(1.1705 - 1.2887)
	0.0146	0.0000	0.0000	0.0136	0.0000	0.0000	0.0068	0.0000	0.0000
12 years or more					omitted				
Ignored				-113.3077***	1.8504***	1.7138***	-109.4409***	1.8653***	1.7621***
				(-141.6878 - -84.9276)	(1.6921 - 2.0235)	(1.3653 - 2.1511)	(-137.7367 - -81.1452)	(1.6998 - 2.0470)	(1.4116 - 2.1996)
				0.0000	0.0000	0.0000	0.0000	0.0000	0.0000
Marital status									
Single					omitted				
Married	20.3900***	0.8521***	0.8189***	20.6428***	0.8511***	0.8169***	20.1844***	0.8468***	0.8119***
	(15.5645 - 25.2156)	(0.8400 - 0.8645)	(0.7878 - 0.8513	(15.9187 - 25.3668)	(0.8387 - 0.8636)	(0.7860 - 0.8491)	(15.8263 - 24.5425)	(0.8350 - 0.8588)	(0.7807 - 0.8444)
	0.0000	0.0000	0.0000	0.0000	0.0000	0.0000	0.0000	0.0000	0.0000
Widow	0.9037	1.0154	0.9694	0.2937	1.0190	0.9604	0.5448	1.0365	0.9791
	(-13.5662 - 15.3736)	(0.9084 - 1.1350)	(0.7997 - 1.1750	(-13.8883 - 14.4757)	(0.9138 - 1.1363)	(0.7914 - 1.1654)	(-12.5195 - 13.6091)	(0.9404 - 1.1425)	(0.8037 - 1.1927)
	0.8988	0.7878	0.7513	0.9664	0.7348	0.6821	0.9323	0.4703	0.8337
Divorced	-1.8403	0.9804	1.0055	-1.4840	0.9794	1.0016	-1.4880	0.9708	0.9910
	(-7.1965 - 3.5159)	(0.9377 - 1.0251)	(0.9255 - 1.0923	(-6.5601 - 3.5921)	(0.9372 - 1.0234)	(0.9207 - 1.0897)	(-6.2060 - 3.2299)	(0.9304 - 1.0131)	(0.9088 - 1.0807)
	0.4863	0.3849	0.8976	0.5531	0.3530	0.9697	0.5225	0.1733	0.8383
Consensual union	15.9632***	0.9682	0.9664	16.0475***	0.9679	0.9647	15.4986***	0.9720	0.9634
	(8.8312 - 23.0951)	(0.9017 - 1.0397)	(0.8661 - 1.0784	(8.9380 - 23.1571)	(0.9015 - 1.0392)	(0.8646 - 1.0763)	(8.6407 - 22.3565)	(0.9100 - 1.0383)	(0.8638 - 1.0744)
	0.0001	0.3743	0.5416	0.0001	0.3686	0.5197	0.0001	0.3993	0.5024
Ignored				-32.2956**	1.1984**	1.5933***	-8.1456	1.0428	1.2126***
				(-57.8893 - -6.7019)	(1.0429 - 1.3770)	(1.1752 - 2.1601)	(-21.1258 - 4.8346)	(0.9602 - 1.1325)	(1.0599 - 1.3872)
				0.0154	0.0107	0.0027	0.2084	0.3197	0.0050
Race									
Asian				omitted					
White	20.9659***	1.0211	1.2093**	19.6750***	1.0292	1.2187**			
	(8.3545 - 33.5773)	(0.9536 - 1.0934)	(1.0280 - 1.4225	(8.3008 - 31.0493)	(0.9569 - 1.1069)	(1.0321 - 1.4390)			
	0.0021	0.5496	0.0218	0.0015	0.4386	0.0197			
Native	165.1503**	0.6906**	0.6427***	2.8751	1.1751**	1.7946***			
	(16.0097 - 314.2909)	(0.4984 - 0.9569)	(0.4895 - 0.8437	(-16.5938 - 22.3439)	(1.0095 - 1.3679)	(1.4028 - 2.2958)			

	0.0313	0.0261	0.0015	0.7639	0.0374	0.0000			
Brown	25.9438***	1.0670*	1.2735***	164.0805**	0.6948**	0.6570***			
	(13.5570 - 38.3306)	(0.9944 - 1.1448)	(1.1180 - 1.4506	(15.5985 - 312.5624)	(0.5013 - 0.9631)	(0.5006 - 0.8623)			
	0.0002	0.0714	0.0003	0.0316	0.0288	0.0025			
Black	-1.6682	1.2028***	1.4664***	24.5328***	1.0763*	1.2867***			
	(-9.4954 - 6.1590)	(1.1046 - 1.3097)	(1.2170 - 1.7669	(13.1808 - 35.8848)	(0.9985 - 1.1601)	(1.1242 - 1.4727)			
	0.6649	0.0000	0.0001	0.0001	0.0547	0.0003			
Ignored				-2.9212	1.2119***	1.4730***			
				(-10.3394 - 4.4969)	(1.1067 - 1.3270)	(1.2177 - 1.7817)			
				0.4256	0.0000	0.0001			
City of residence (code)									
Porto Velho (1)					omitted				
Rio Branco (2)	-91.6626***	1.2059***	0.8941**	-95.8308***	1.2412***	0.8880**	-86.7563***	1.0866***	0.9968
	(-100.2316 - -83.0935)	(1.1442 - 1.2709)	(0.8171 - 0.9783	(-104.3681 - -87.2935)	(1.1780 - 1.3079)	(0.8091 - 0.9746)	(-94.6521 - -78.8606)	(1.0299 - 1.1464)	(0.9105 - 1.0914)
	0.0000	0.0000	0.0148	0.0000	0.0000	0.0124	0.0000	0.0024	0.9454
Manaus (3)	-37.7619***	1.0445	0.5238***	-37.7976***	1.0472	0.5221***	-36.7420***	1.0108	0.5261***
	(-48.3360 - -27.1879)	(0.9735 - 1.1206)	(0.4517 - 0.6074	(-48.2905 - -27.3046)	(0.9765 - 1.1231)	(0.4495 - 0.6064)	(-46.9709 - -26.5130)	(0.9408 - 1.0860)	(0.4561 - 0.6069)
	0.0000	0.2254	0.0000	0.0000	0.1960	0.0000	0.0000	0.7696	0.0000
Boa Vista (4)	-34.8745***	1.1799***	0.5214***	-34.8910***	1.1777***	0.5136***	-31.9560***	1.1622***	0.5230***
	(-41.1983 - -28.5508)	(1.1388 - 1.2225)	(0.4825 - 0.5635	(-41.2502 - -28.5318)	(1.1360 - 1.2209)	(0.4756 - 0.5547)	(-37.6460 - -26.2661)	(1.1160 - 1.2103)	(0.4809 - 0.5689)
	0.0000	0.0000	0.0000	0.0000	0.0000	0.0000	0.0000	0.0000	0.0000
Belem (5)	-129.7426***	1.1588***	0.7853***	-127.4094***	1.1519***	0.7653***	-128.7547***	1.1487***	0.8102**
	(-142.2240 - -117.2612)	(1.0716 - 1.2531)	(0.6612 - 0.9327	(-140.0187 - -114.8001)	(1.0648 - 1.2462)	(0.6422 - 0.9121)	(-140.6931 - -116.8163)	(1.0633 - 1.2411)	(0.6872 - 0.9552)
	0.0000	0.0002	0.0059	0.0000	0.0004	0.0028	0.0000	0.0004	0.0122
Macapa (6)	-103.9206***	1.6371***	0.8549	-104.3813***	1.6487***	0.8744	-109.0674***	1.5997***	1.0035
	(-119.4754 - -88.3658)	(1.4867 - 1.8027)	(0.7088 - 1.0311	(-119.9838 - -88.7788)	(1.4977 - 1.8150)	(0.7216 - 1.0596)	(-123.0947 - -95.0402)	(1.4612 - 1.7513)	(0.8435 - 1.1938)
	0.0000	0.0000	0.1012	0.0000	0.0000	0.1710	0.0000	0.0000	0.9686
Palmas (7)	-62.6570***	0.8979***	0.4125***	-63.9244***	0.9131***	0.4066***	-65.3664***	0.8629***	0.3879***
	(-70.8850 - -54.4291)	(0.8573 - 0.9405)	(0.3682 - 0.4620	(-72.0985 - -55.7503)	(0.8712 - 0.9570)	(0.3639 - 0.4544)	(-73.8453 - -56.8874)	(0.8166 - 0.9118)	(0.3426 - 0.4392)
	0.0000	0.0000	0.0000	0.0000	0.0002	0.0000	0.0000	0.0000	0.0000
São Luis (8)	-79.2924***	1.8550***	1.1205	-76.3684***	1.8444***	1.0104	-93.0294***	1.7704***	1.1486
	(-97.8011 - -60.7838)	(1.6585 - 2.0748)	(0.8898 - 1.4110	(-95.5459 - -57.1908)	(1.6440 - 2.0692)	(0.8000 - 1.2762)	(-110.4405 - -75.6184)	(1.5835 - 1.9794)	(0.9151 - 1.4417)
	0.0000	0.0000	0.3334	0.0000	0.0000	0.9309	0.0000	0.0000	0.2322
Teresina (9)	-79.4016***	1.3755***	0.7015***	-82.4522***	1.3963***	0.7096***	-76.5971***	1.3374***	0.6932***
	(-85.7505 - -73.0527)	(1.3044 - 1.4505)	(0.6293 - 0.7821	(-88.7953 - -76.1091)	(1.3238 - 1.4728)	(0.6358 - 0.7920)	(-82.7552 - -70.4391)	(1.2636 - 1.4155)	(0.6192 - 0.7760)
	0.0000	0.0000	0.0000	0.0000	0.0000	0.0000	0.0000	0.0000	0.0000
Fortaleza (10)	-87.2175***	1.3535***	0.9648	-86.3371***	1.3635***	0.9351	-80.6152***	1.2947***	0.8727***

	(-93.4529 - -80.9820)	(1.3036 - 1.4053)	(0.8800 - 1.0577	(-92.5660 - -80.1083)	(1.3124 - 1.4165)	(0.8516 - 1.0268)	(-86.9084 - -74.3220)	(1.2426 - 1.3490)	(0.7917 - 0.9619)
	0.0000	0.0000	0.4446	0.0000	0.0000	0.1597	0.0000	0.0000	0.0061
Natal (11)	-89.4971***	1.6933***	0.9199	-90.7660***	1.7276***	0.9474	-92.2665***	1.6970***	0.9594
	(-100.1335 - -78.8606)	(1.5745 - 1.8211)	(0.7990 - 1.0592	(-101.4845 - -80.0475)	(1.6061 - 1.8582)	(0.8212 - 1.0929)	(-101.9126 - -82.6204)	(1.5794 - 1.8234)	(0.8353 - 1.1020)
	0.0000	0.0000	0.2461	0.0000	0.0000	0.4583	0.0000	0.0000	0.5580
Joao Pessoa (12)	-26.8586***	1.1418***	0.6046***	-26.3192***	1.1491***	0.6080***	-29.3182***	1.1411***	0.6423***
	(-35.1483 - -18.5689)	(1.0761 - 1.2116)	(0.5390 - 0.6783	(-34.6429 - -17.9955)	(1.0825 - 1.2199)	(0.5399 - 0.6847)	(-37.0031 - -21.6332)	(1.0753 - 1.2110)	(0.5731 - 0.7199)
	0.0000	0.0000	0.0000	0.0000	0.0000	0.0000	0.0000	0.0000	0.0000
Recife (13)	-85.3372***	1.3173***	0.9698	-85.5184***	1.3182***	0.9722	-90.4494***	1.3200***	1.0032
	(-93.5998 - -77.0746)	(1.2381 - 1.4015)	(0.8578 - 1.0964	(-93.8239 - -77.2129)	(1.2388 - 1.4026)	(0.8593 - 1.1000)	(-98.3838 - -82.5151)	(1.2363 - 1.4093)	(0.8837 - 1.1389)
	0.0000	0.0000	0.6240	0.0000	0.0000	0.6547	0.0000	0.0000	0.9604
Maceio (14)	-123.2551***	1.3952***	0.6772***	-123.1163***	1.4062***	0.6729***	-123.4979***	1.3825***	0.7125***
	(-137.5355 - -108.9748)	(1.2643 - 1.5397)	(0.5558 - 0.8251	(-137.4874 - -108.7452)	(1.2742 - 1.5518)	(0.5507 - 0.8224)	(-136.8755 - -110.1202)	(1.2519 - 1.5267)	(0.5872 - 0.8646)
	0.0000	0.0000	0.0001	0.0000	0.0000	0.0001	0.0000	0.0000	0.0006
Aracaju (15)	-52.3085***	1.3915***	1.1274	-52.1662***	1.3955***	1.1174	-54.6358***	1.3740***	1.1629*
	(-63.8947 - -40.7223)	(1.2838 - 1.5082)	(0.9604 - 1.3234	(-63.8541 - -40.4782)	(1.2878 - 1.5122)	(0.9490 - 1.3156)	(-65.1604 - -44.1111)	(1.2684 - 1.4882)	(0.9950 - 1.3591)
	0.0000	0.0000	0.1427	0.0000	0.0000	0.1829	0.0000	0.0000	0.0579
Salvador (16)	-106.5716***	1.7355***	1.2609***	-107.2501***	1.7533***	1.2531***	-112.0422***	1.7822***	1.3185***
	(-111.9003 - -101.2428)	(1.6814 - 1.7913)	(1.1739 - 1.3544	(-112.5081 - -101.9921)	(1.6986 - 1.8097)	(1.1659 - 1.3469)	(-117.1369 - -106.9475)	(1.7198 - 1.8469)	(1.2194 - 1.4257)
	0.0000	0.0000	0.0000	0.0000	0.0000	0.0000	0.0000	0.0000	0.0000
Belo Horizonte (17)	-162.9273***	1.5633***	0.9612	-162.9490***	1.5700***	0.9451	-169.9438***	1.5995***	1.0119
	(-171.2376 - -154.6170)	(1.5007 - 1.6285)	(0.8787 - 1.0515	(-171.1515 - -154.7466)	(1.5072 - 1.6353)	(0.8644 - 1.0332)	(-178.1729 - -161.7147)	(1.5366 - 1.6651)	(0.9218 - 1.1109)
	0.0000	0.0000	0.3880	0.0000	0.0000	0.2144	0.0000	0.0000	0.8037
Vitoria (18)	-86.2602***	1.3083***	0.6807***	-82.3464***	1.2746***	0.7082***	-81.2752***	1.2486***	0.7003***
	(-91.1012 - -81.4192)	(1.2512 - 1.3679)	(0.6282 - 0.7376	(-86.9709 - -77.7219)	(1.2176 - 1.3343)	(0.6506 - 0.7709)	(-86.4065 - -76.1438)	(1.1928 - 1.3071)	(0.6441 - 0.7615)
	0.0000	0.0000	0.0000	0.0000	0.0000	0.0000	0.0000	0.0000	0.0000
Rio de Janeiro (19)	-90.0692***	1.3781***	0.8518***	-90.9821***	1.3943***	0.8507***	-97.9109***	1.3985***	0.8869***
	(-94.1711 - -85.9673)	(1.3340 - 1.4237)	(0.8019 - 0.9048	(-95.0264 - -86.9378)	(1.3486 - 1.4415)	(0.7968 - 0.9082)	(-101.9945 - -93.8273)	(1.3517 - 1.4468)	(0.8314 - 0.9462)
	0.0000	0.0000	0.0000	0.0000	0.0000	0.0000	0.0000	0.0000	0.0003
São Paulo (20)	-128.6981***	1.4802***	0.9191***	-129.4227***	1.4948***	0.9118***	-134.7024***	1.4688***	0.9249**
	(-134.8563 - -122.5399)	(1.4387 - 1.5230)	(0.8675 - 0.9737	(-135.5136 - -123.3319)	(1.4531 - 1.5377)	(0.8609 - 0.9656)	(-140.7551 - -128.6498)	(1.4281 - 1.5107)	(0.8688 - 0.9846)
	0.0000	0.0000	0.0042	0.0000	0.0000	0.0016	0.0000	0.0000	0.0144
Curitiba (21)	-92.1471***	1.1302***	0.8616**	-93.2222***	1.1428***	0.8513***	-99.3297***	1.1066***	0.8568***
	(-103.8571 - -80.4370)	(1.0733 - 1.1901)	(0.7692 - 0.9652	(-104.8241 - -81.6202)	(1.0857 - 1.2028)	(0.7596 - 0.9540)	(-110.4881 - -88.1714)	(1.0534 - 1.1625)	(0.7668 - 0.9574)
	0.0000	0.0000	0.0101	0.0000	0.0000	0.0056	0.0000	0.0001	0.0064
Florianopolis (22)	-34.6113***	1.5187***	0.9452	-34.1668***	1.5114***	0.9219	-30.9468***	1.4333***	0.8744**
	(-46.1755 - -23.0470)	(1.4508 - 1.5897)	(0.8362 - 1.0685	(-45.5286 - -22.8049)	(1.4455 - 1.5802)	(0.8156 - 1.0420)	(-42.4679 - -19.4257)	(1.3696 - 1.5000)	(0.7820 - 0.9778)

	0.0000	0.0000	0.3678	0.0000	0.0000	0.1930	0.0000	0.0000	0.0185
Porto Alegre (23)	-62.5265***	1.3443***	0.7643***	-64.3875***	1.3535***	0.7525***	-73.8088***	1.3353***	0.7500***
	(-72.9713 - -52.0816)	(1.2857 - 1.4057)	(0.6809 - 0.8579	(-74.6749 - -54.1002)	(1.2951 - 1.4145)	(0.6702 - 0.8450)	(-84.0606 - -63.5570)	(1.2797 - 1.3932)	(0.6738 - 0.8349)
	0.0000	0.0000	0.0000	0.0000	0.0000	0.0000	0.0000	0.0000	0.0000
Campo Grande (24)	-94.8739***	1.2592***	0.8736***	-96.1485***	1.2754***	0.8738***	-99.3466***	1.2461***	0.8829***
	(-98.4557 - -91.2921)	(1.2227 - 1.2968)	(0.8367 - 0.9121	(-99.7913 - -92.5057)	(1.2388 - 1.3131)	(0.8369 - 0.9123)	(-102.6115 - -96.0817)	(1.2130 - 1.2801)	(0.8408 - 0.9270)
	0.0000	0.0000	0.0000	0.0000	0.0000	0.0000	0.0000	0.0000	0.0000
Cuiaba (25)	-56.3609***	1.1724***	0.9300	-56.7393***	1.1829***	0.9188	-63.4112***	1.2095***	0.9370
	(-66.9193 - -45.8025)	(1.1283 - 1.2183)	(0.8268 - 1.0460	(-67.1364 - -46.3421)	(1.1390 - 1.2285)	(0.8186 - 1.0311)	(-74.0078 - -52.8146)	(1.1576 - 1.2637)	(0.8296 - 1.0583)
	0.0000	0.0000	0.2263	0.0000	0.0000	0.1501	0.0000	0.0000	0.2951
Goiania (26)	-127.1380***	1.2766***	0.4945***	-126.4547***	1.3034***	0.4748***	-117.4920***	1.2557***	0.5443***
	(-138.2853 - -115.9908)	(1.2228 - 1.3328)	(0.4441 - 0.5506	(-137.3153 - -115.5941)	(1.2506 - 1.3585)	(0.4254 - 0.5301)	(-127.9872 - -106.9967)	(1.2044 - 1.3093)	(0.4915 - 0.6027)
	0.0000	0.0000	0.0000	0.0000	0.0000	0.0000	0.0000	0.0000	0.0000
Brasilia (27)	-122.2085***	1.4782***	0.7570***	-117.4082***	1.4659***	0.7080***	-131.9891***	1.5518***	0.7814***
	(-128.2771 - -116.1399)	(1.4193 - 1.5396)	(0.6926 - 0.8274	(-123.0619 - -111.7545)	(1.4015 - 1.5332)	(0.6420 - 0.7808)	(-138.1889 - -125.7893)	(1.4835 - 1.6233)	(0.7099 - 0.8601)
	0.0000	0.0000	0.0000	0.0000	0.0000	0.0000	0.0000	0.0000	0.0000
Month									
1					omitted				
2	27.4055***	0.8376***	0.5036***	27.9530***	0.8324***	0.5023***	19.3307***	0.8641***	0.5186***
	(20.0249 - 34.7861)	(0.8030 - 0.8737)	(0.4681 - 0.5417	(20.3835 - 35.5224)	(0.7988 - 0.8674)	(0.4663 - 0.5411)	(12.2794 - 26.3821)	(0.8310 - 0.8984)	(0.4838 - 0.5559)
	0.0000	0.0000	0.0000	0.0000	0.0000	0.0000	0.0000	0.0000	0.0000
3	9.1501***	0.8732***	0.6312***	8.3784***	0.8726***	0.6507***	4.8149*	0.8578***	0.5765***
	(3.3307 - 14.9695)	(0.8409 - 0.9069)	(0.5605 - 0.7108	(2.3465 - 14.4102)	(0.8408 - 0.9055)	(0.5774 - 0.7332)	(-0.4874 - 10.1171)	(0.8300 - 0.8865)	(0.5152 - 0.6450)
	0.0033	0.0000	0.0000	0.0083	0.0000	0.0000	0.0733	0.0000	0.0000
4	-16.3351***	1.0604***	0.6878***	-8.4099***	1.0613***	0.6885***	-7.7572***	1.0907***	0.7121***
	(-22.8212 - -9.8490)	(1.0229 - 1.0993)	(0.6176 - 0.7659	(-13.7621 - -3.0577)	(1.0239 - 1.1001)	(0.6230 - 0.7609)	(-12.3199 - -3.1945)	(1.0564 - 1.1262)	(0.6450 - 0.7861)
	0.0000	0.0014	0.0000	0.0033	0.0012	0.0000	0.0017	0.0000	0.0000
5	14.4310***	0.9035***	0.5795***	14.1992***	0.9015***	0.5755***	1.6279	0.9193***	0.5610***
	(7.5653 - 21.2968)	(0.8618 - 0.9474)	(0.5294 - 0.6343	(7.1913 - 21.2070)	(0.8611 - 0.9438)	(0.5288 - 0.6263)	(-5.7308 - 8.9865)	(0.8788 - 0.9616)	(0.5171 - 0.6086)
	0.0002	0.0000	0.0000	0.0003	0.0000	0.0000	0.6531	0.0002	0.0000
6	10.2508***	1.0021	0.6391***	17.2413***	0.9995	0.6343***	9.9802**	0.9935	0.6433***
	(4.3448 - 16.1567)	(0.9676 - 1.0379)	(0.5753 - 0.7101	(8.9553 - 25.5272)	(0.9645 - 1.0358)	(0.5748 - 0.7000)	(2.3891 - 17.5713)	(0.9573 - 1.0312)	(0.5868 - 0.7052)
	0.0014	0.9054	0.0000	0.0002	0.9795	0.0000	0.0120	0.7330	0.0000
7	9.5817***	0.9401**	0.7744***	0.1672	0.9522**	0.7986***	-0.1843	0.9586*	0.8335***
	(4.1167 - 15.0468)	(0.8969 - 0.9855)	(0.7014 - 0.8549	(-5.1739 - 5.5083)	(0.9088 - 0.9977)	(0.7266 - 0.8778)	(-6.4470 - 6.0784)	(0.9155 - 1.0038)	(0.7572 - 0.9175)
	0.0013	0.0102	0.0000	0.9492	0.0397	0.0000	0.9522	0.0724	0.0002
8	-4.9927	1.0162	0.6195***	-4.4612	1.0121	0.6167***	-9.4884**	0.9628*	0.5961***

	(-13.3437 - 3.3582)	(0.9778 - 1.0561)	(0.5587 - 0.6871	(-12.7873 - 3.8648)	(0.9742 - 1.0516)	(0.5564 - 0.6835)	(-17.0860 - -1.8908)	(0.9258 - 1.0011)	(0.5366 - 0.6622)
	0.2301	0.4127	0.0000	0.2808	0.5364	0.0000	0.0164	0.0571	0.0000
9	20.7373***	0.9530	0.6992***	20.8965***	0.9456*	0.6930***	13.4962***	0.9401**	0.6335***
	(13.6510 - 27.8236)	(0.8941 - 1.0157)	(0.6018 - 0.8124	(14.0261 - 27.7668)	(0.8887 - 1.0061)	(0.5976 - 0.8035)	(6.9264 - 20.0659)	(0.8889 - 0.9942)	(0.5540 - 0.7243)
	0.0000	0.1385	0.0000	0.0000	0.0769	0.0000	0.0003	0.0306	0.0000
10	14.3884*	0.9559	0.7289***	3.5195	0.9645	0.7276***	6.4098	0.9551	0.7156***
	(-0.6301 - 29.4068)	(0.8914 - 1.0251)	(0.6193 - 0.8579	(-2.8243 - 9.8634)	(0.8994 - 1.0344)	(0.6203 - 0.8533)	(-8.1503 - 20.9699)	(0.8935 - 1.0210)	(0.6200 - 0.8259)
	0.0597	0.2062	0.0001	0.2645	0.3114	0.0001	0.3738	0.1770	0.0000
11	6.6591	1.1013***	0.6626***	-3.5907	1.0986***	0.6635***	1.7875	1.0820***	0.7000***
	(-5.9118 - 19.2300)	(1.0441 - 1.1617)	(0.5700 - 0.7703	(-16.2028 - 9.0213)	(1.0405 - 1.1601)	(0.5720 - 0.7697)	(-10.6495 - 14.2244)	(1.0238 - 1.1436)	(0.6075 - 0.8066)
	0.2862	0.0004	0.0000	0.5634	0.0007	0.0000	0.7700	0.0052	0.0000
12	23.0744***	1.1225***	0.5909***	12.7567*	1.1284***	0.5856***	20.3570***	1.0610**	0.5636***
	(14.8750 - 31.2738)	(1.0722 - 1.1751)	(0.5248 - 0.6653	(-2.1481 - 27.6614)	(1.0786 - 1.1805)	(0.5201 - 0.6594)	(12.2688 - 28.4452)	(1.0139 - 1.1103)	(0.5061 - 0.6276)
	0.0000	0.0000	0.0000	0.0903	0.0000	0.0000	0.0000	0.0105	0.0000
Year									
2012					omitted				
2013	-9.4570**	1.0024	1.0096	-20.7454**	1.0016	0.9957	-4.3867	0.9898	1.0094
	(-18.4288 - -0.4851)	(0.9528 - 1.0546)	(0.8922 - 1.1425	(-36.1809 - -5.3099)	(0.9537 - 1.0519)	(0.8791 - 1.1279)	(-13.3981 - 4.6248)	(0.9439 - 1.0379)	(0.9055 - 1.1253)
	0.0396	0.9254	0.8792	0.0104	0.9489	0.9464	0.3262	0.6717	0.8654
2014	-27.6405***	1.2914***	1.3542***	-38.5531***	1.2914***	1.3223***	-26.0616***	1.2668***	1.2798***
	(-45.1498 - -10.1311)	(1.2214 - 1.3654)	(1.2015 - 1.5264	(-51.8528 - -25.2534)	(1.2217 - 1.3651)	(1.1771 - 1.4853)	(-43.3164 - -8.8068)	(1.1985 - 1.3391)	(1.1566 - 1.4161)
	0.0032	0.0000	0.0000	0.0000	0.0000	0.0000	0.0046	0.0000	0.0000
2015	-63.2378***	1.6495***	1.6372***	-74.8886***	1.6562***	1.6254***	-51.1483***	1.5664***	1.5183***
	(-79.3647 - -47.1109)	(1.5682 - 1.7351)	(1.4756 - 1.8166	(-93.9664 - -55.8108)	(1.5774 - 1.7390)	(1.4640 - 1.8045)	(-66.8350 - -35.4616)	(1.4934 - 1.6430)	(1.3668 - 1.6866)
	0.0000	0.0000	0.0000	0.0000	0.0000	0.0000	0.0000	0.0000	0.0000
2016	-51.1320***	1.6060***	1.5086***	-63.5612***	1.6228***	1.5261***	-48.6418***	1.5889***	1.5628***
	(-68.0260 - -34.2379)	(1.5043 - 1.7146)	(1.2684 - 1.7942	(-77.4326 - -49.6897)	(1.5196 - 1.7329)	(1.2848 - 1.8128)	(-65.7980 - -31.4855)	(1.4843 - 1.7009)	(1.3167 - 1.8549)
	0.0000	0.0000	0.0000	0.0000	0.0000	0.0000	0.0000	0.0000	0.0000
Constant	3,230.5447***	0.0583***	0.0106***	3,244.1008***	0.0571***	0.0105***	3,260.2495***	0.0606***	0.0131***
	(3,203.2885 - 3,257.8008)	(0.0502 - 0.0677)	(0.0086 - 0.0129	(3,214.4871 - 3,273.7145)	(0.0492 - 0.0663)	(0.0086 - 0.0129)	(3,235.6262 - 3,284.8728)	(0.0550 - 0.0667)	(0.0113 - 0.0152)
	0.0000	0.0000	0.0000	0.0000	0.0000	0.0000	0.0000	0.0000	0.0000
Observations	2,457,972	2,457,972	2,457,972	2,474,646	2,474,646	2,474,646	2,594,223	2,594,223	2,594,223
R-squared	0.0167			0.0167			0.0162		

Note: coefficients of interaction terms are not reported. *** p<0.01, ** p<0.05, * p<0.1
