# Supplementary material for: In utero exposure to economic fluctuations and birth outcomes: An analysis of the relevance of the local unemployment rate in Brazilian state capitals
Source: PLoS One. 2019 Oct 10;14(10):e0223673. doi: 10.1371/journal.pone.0223673 (PMC6786569; doi:10.1371/journal.pone.0223673)
Supplement: S3 Table — (PDF) [file pone.0223673.s003.pdf]

S3 Table. Extended version of table 4.

	Age		Marital status		Race/ethnicity		Years of education	
	≤ 24 years	≥ 25 years	Partner	No Partner	Black or Brown	White	11 or less	12 or more
	(1)	(2)	(3)	(4)	(5)	(6)	(7)	(8)
	VLBW	VLBW	VLBW	VLBW	VLBW	VLBW	VLBW	VLBW
Unemployment rate months 7 to 9 before birth	1.0523**	0.9815	1.0195	1.0019	1.0111	0.9858	1.0224	0.9788
95% CI	(1.0115 - 1.0948)	(0.9526 - 1.0114)	(0.9941 - 1.0456)	(0.9676 - 1.0373)	(0.9858 - 1.0371)	(0.9326 - 1.0421)	(0.9918 - 1.0539)	(0.9389 - 1.0203)
p-val	0.0116	0.2228	0.1338	0.9169	0.3938	0.6145	0.1528	0.3120
Unemployment rate months 4 to 6 before birth	0.9636	0.9929	0.9843	0.9789	0.9880	0.9754	0.9740*	1.0104
95% CI	(0.9136 - 1.0164)	(0.9617 - 1.0252)	(0.9436 - 1.0268)	(0.9451 - 1.0139)	(0.9604 - 1.0164)	(0.9153 - 1.0395)	(0.9440 - 1.0049)	(0.9360 - 1.0907)
p-val	0.1732	0.6635	0.4626	0.2348	0.4036	0.4430	0.0986	0.7912
Unemployment rate months 1 to 3 before birth	1.0684***	0.9992	1.0354*	1.0080	1.0387***	0.9946	1.0477***	0.9561
95% CI	(1.0353 - 1.1024)	(0.9633 - 1.0364)	(0.9938 - 1.0787)	(0.9781 - 1.0388)	(1.0156 - 1.0624)	(0.9247 - 1.0697)	(1.0245 - 1.0714)	(0.8934 - 1.0233)
p-val	0.0000	0.9650	0.0965	0.6049	0.0009	0.8834	0.0000	0.1950
Maternal characteristics								
Age								
<= 19			1.0434*	1.0440*	0.9837	0.9935	1.0114	1.1775*
			(0.9946 - 1.0947)	(0.9946 - 1.0959)	(0.9325 - 1.0377)	(0.9125 - 1.0817)	(0.9687 - 1.0561)	(0.9703 - 1.4290)
			0.0822	0.0814	0.5470	0.8807	0.6058	0.0980
20-24			0.8848***	0.8848***	0.8798***	0.8289***	0.8765***	0.8980***
			(0.8485 - 0.9226)	(0.8479 - 0.9234)	(0.8469 - 0.9140)	(0.7805 - 0.8804)	(0.8435 - 0.9108)	(0.8415 - 0.9582)
			0.0000	0.0000	0.0000	0.0000	0.0000	0.0012
25-34					omitted			
>= 35			1.3115***	1.3118***	1.4085***	1.2829***	1.4214***	1.2902***
			(1.2500 - 1.3760)	(1.2468 - 1.3803)	(1.3418 - 1.4786)	(1.1889 - 1.3843)	(1.3482 - 1.4986)	(1.1976 - 1.3899)
			0.0000	0.0000	0.0000	0.0000	0.0000	0.0000
Previous children								
At least 1 alive	0.7095***	0.5835***	0.5824***	0.6738***	0.6400***	0.5822***	0.6349***	0.5892***
	(0.6711 - 0.7500)	(0.5594 - 0.6087)	(0.5591 - 0.6066)	(0.6393 - 0.7102)	(0.6051 - 0.6769)	(0.5599 - 0.6055)	(0.6040 - 0.6673)	(0.5669 - 0.6123)
	0.0000	0.0000	0.0000	0.0000	0.0000	0.0000	0.0000	0.0000
At least 1 dead	1.4087***	1.5499***	1.5615***	1.4027***	1.4488***	1.5484***	1.4874***	1.4685***
	(1.2870 - 1.5418)	(1.4915 - 1.6106)	(1.4846 - 1.6424)	(1.3238 - 1.4862)	(1.3610 - 1.5422)	(1.4812 - 1.6187)	(1.3890 - 1.5928)	(1.4032 - 1.5369)
	0.0000	0.0000	0.0000	0.0000	0.0000	0.0000	0.0000	0.0000

Education								
None	1.6243**	1.8953***	1.5447*	1.7928***	1.5241***	1.8859*		
	(1.0037 - 2.6286)	(1.4808 - 2.4259)	(0.9974 - 2.3923)	(1.4429 - 2.2275)	(1.2008 - 1.9344)	(0.9744 - 3.6498)		
	0.0483	0.0000	0.0514	0.0000	0.0005	0.0597		
1 - 3 years	1.3302***	1.4601***	1.4863***	1.3231***	1.2310***	1.5141***		
	(1.1847 - 1.4935)	(1.2956 - 1.6456)	(1.2851 - 1.7191)	(1.2035 - 1.4545)	(1.1279 - 1.3435)	(1.1587 - 1.9785)		
	0.0000	0.0000	0.0000	0.0000	0.0000	0.0024		
4 - 7 years	1.2009***	1.2801***	1.3932***	1.1661***	1.0848**	1.5305***		
	(1.0978 - 1.3136)	(1.1938 - 1.3726)	(1.2744 - 1.5230)	(1.0948 - 1.2422)	(1.0111 - 1.1638)	(1.4357 - 1.6316)		
	0.0001	0.0000	0.0000	0.0000	0.0234	0.0000		
8 - 11 years	1.0870**	1.2155***	1.3136***	1.1038***	1.0610**	1.3492***		
	(1.0069 - 1.1734)	(1.1517 - 1.2829)	(1.2192 - 1.4154)	(1.0542 - 1.1557)	(1.0092 - 1.1155)	(1.2905 - 1.4106)		
	0.0327	0.0000	0.0000	0.0000	0.0205	0.0000		
12 years or more			omitted					
Ignored	1.5885***	1.5039***	1.2037	1.6089***	1.4885***	1.3256		
	(1.3201 - 1.9115)	(1.1298 - 2.0019)	(0.9252 - 1.5661)	(1.2846 - 2.0151)	(1.1086 - 1.9984)	(0.9194 - 1.9111)		
	0.0000	0.0052	0.1672	0.0000	0.0081	0.1310		
Marital status								
Single	omitted					omitted		
Married	0.7971***	0.8391***			0.8466***	0.7883***	0.8492***	0.7687***
	(0.7389 - 0.8599)	(0.8116 - 0.8675)			(0.8065 - 0.8886)	(0.7405 - 0.8393)	(0.8140 - 0.8859)	(0.7224 - 0.8180)
	0.0000	0.0000			0.0000	0.0000	0.0000	0.0000
Widow	0.5757	1.1421			0.9147	1.1200	0.9615	1.0553
	(0.2188 - 1.5144)	(0.9103 - 1.4330)			(0.7373 - 1.1347)	(0.7765 - 1.6156)	(0.7942 - 1.1640)	(0.5504 - 2.0234)
	0.2631	0.2509			0.4173	0.5442	0.6871	0.8712
Divorced	1.1304	1.0656			0.9815	1.0396	0.9827	0.9901
	(0.8593 - 1.4871)	(0.9790 - 1.1599)			(0.9109 - 1.0575)	(0.8953 - 1.2072)	(0.8800 - 1.0974)	(0.8300 - 1.1811)
	0.3809	0.1419			0.6234	0.6104	0.7567	0.9121
Consensual union	0.9198	1.0009			0.9599	0.9968	0.9740	0.9803
	(0.8162 - 1.0365)	(0.9005 - 1.1125)			(0.8570 - 1.0753)	(0.8729 - 1.1383)	(0.8701 - 1.0902)	(0.8887 - 1.0814)
	0.1701	0.9866			0.4801	0.9626	0.6469	0.6910
Ignored	1.0416	1.2793***			1.3694***	1.1404*	1.0866	1.1659
	(0.8680 - 1.2500)	(1.0673 - 1.5334)			(1.1269 - 1.6641)	(0.9788 - 1.3286)	(0.9099 - 1.2976)	(0.8279 - 1.6417)

	0.6610	0.0077			0.0016	0.0919	0.3590	0.3796
Race								
Asian	omitted						omitted	
White	1.0879	1.2534***	1.2359**	1.2027*			1.2078**	1.1780**
	(0.8684 - 1.3631)	(1.1238 - 1.3980)	(1.0459 - 1.4605)	(0.9952 - 1.4534)			(1.0311 - 1.4149)	(1.0148 - 1.3675)
	0.4637	0.0000	0.0129	0.0561			0.0193	0.0313
Native	0.5322***	0.7720	0.8818	0.5246***			0.6526**	0.5630
	(0.3428 - 0.8262)	(0.5498 - 1.0839)	(0.6950 - 1.1187)	(0.3221 - 0.8544)			(0.4693 - 0.9073)	(0.1748 - 1.8137)
	0.0049	0.1350	0.3002	0.0095			0.0111	0.3358
Brown	1.0579	1.3744***	1.3784***	1.2069**			1.1926**	1.4278***
	(0.8843 - 1.2656)	(1.2344 - 1.5302)	(1.1734 - 1.6192)	(1.0377 - 1.4037)			(1.0425 - 1.3642)	(1.2244 - 1.6649)
	0.5383	0.0000	0.0001	0.0147			0.0103	0.0000
Black	1.1710	1.6013***	1.6420***	1.3550***			1.3321***	1.9500***
	(0.9084 - 1.5096)	(1.4083 - 1.8207)	(1.3572 - 1.9866)	(1.0834 - 1.6947)			(1.0983 - 1.6157)	(1.6276 - 2.3362)
	0.2230	0.0000	0.0000	0.0078			0.0036	0.0000
Ignored	1.2958	1.3693***	1.4886***	1.3164***			1.3290**	1.3055
	(0.9320 - 1.8015)	(1.2105 - 1.5489)	(1.1499 - 1.9270)	(1.1076 - 1.5646)			(1.0235 - 1.7258)	(0.9320 - 1.8287)
	0.1233	0.0000	0.0025	0.0018			0.0328	0.1211
City of residence (code)								
Porto Velho (1)	omitted							
Rio Branco (2)	0.8358***	0.9409	1.3434***	0.0000***	0.6591***	3.2759***	0.9345	0.7526**
	(0.7396 - 0.9445)	(0.8170 - 1.0837)	(1.1603 - 1.5555)	(0.0000 - 0.0000)	(0.6029 - 0.7206)	(2.5279 - 4.2453)	(0.8552 - 1.0212)	(0.6040 - 0.9379)
	0.0040	0.3984	0.0001	0.0000	0.0000	0.0000	0.1346	0.0114
Manaus (3)	0.6121***	0.4675***	0.4691***	0.5800***	0.6278***	0.2171***	0.5987***	0.2521***
	(0.5182 - 0.7229)	(0.3827 - 0.5712)	(0.3854 - 0.5709)	(0.4673 - 0.7200)	(0.5453 - 0.7227)	(0.1391 - 0.3389)	(0.5199 - 0.6894)	(0.1918 - 0.3314)
	0.0000	0.0000	0.0000	0.0000	0.0000	0.0000	0.0000	0.0000
Boa Vista (4)	0.9165*	0.3755***	0.4074***	0.4681***	0.5729***	0.6597***	0.4935***	0.4523***
	(0.8375 - 1.0031)	(0.3352 - 0.4206)	(0.3627 - 0.4577)	(0.4265 - 0.5139)	(0.5345 - 0.6140)	(0.5271 - 0.8258)	(0.4594 - 0.5302)	(0.3796 - 0.5388)
	0.0583	0.0000	0.0000	0.0000	0.0000	0.0003	0.0000	0.0000
Belem (5)	1.5919***	0.5275***	0.6542***	1.0410	0.9144	0.4449***	0.9783	0.3405***
	(1.2459 - 2.0340)	(0.4259 - 0.6534)	(0.5115 - 0.8367)	(0.7977 - 1.3586)	(0.7694 - 1.0867)	(0.2800 - 0.7068)	(0.8159 - 1.1731)	(0.2482 - 0.4673)
	0.0002	0.0000	0.0007	0.7671	0.3097	0.0006	0.8129	0.0000
Macapa (6)	2.6472***	0.3187***	0.9152	1.0640	1.0950	0.5651**	1.0583	0.5054***

	(2.0637 - 3.3957)	(0.2513 - 0.4041)	(0.7036 - 1.1903)	(0.8098 - 1.3979)	(0.9031 - 1.3275)	(0.3340 - 0.9561)	(0.8802 - 1.2723)	(0.3564 - 0.7169)
	0.0000	0.0000	0.5086	0.6562	0.3559	0.0334	0.5468	0.0001
Palmas (7)	1.1614*	0.1831***	0.2826***	0.5946***	0.4799***	0.0992***	0.5074***	0.1278***
	(0.9864 - 1.3676)	(0.1588 - 0.2111)	(0.2405 - 0.3320)	(0.5269 - 0.6711)	(0.4319 - 0.5333)	(0.0757 - 0.1301)	(0.4502 - 0.5718)	(0.1043 - 0.1565)
	0.0726	0.0000	0.0000	0.0000	0.0000	0.0000	0.0000	0.0000
São Luis (8)	1.0280	1.1769	0.9015	1.1976	1.2224*	0.6404	1.1272	0.9399
	(0.7421 - 1.4240)	(0.8645 - 1.6022)	(0.6588 - 1.2334)	(0.8119 - 1.7666)	(0.9660 - 1.5469)	(0.3427 - 1.1969)	(0.8865 - 1.4332)	(0.5738 - 1.5397)
	0.8682	0.3006	0.5166	0.3633	0.0945	0.1625	0.3286	0.8057
Teresina (9)	1.2241***	0.4743***	0.6732***	0.2707***	0.8443***	0.5109***	0.7653***	0.2908***
	(1.0843 - 1.3821)	(0.4047 - 0.5558)	(0.5801 - 0.7813)	(0.2357 - 0.3110)	(0.7652 - 0.9316)	(0.3856 - 0.6770)	(0.6972 - 0.8401)	(0.2264 - 0.3735)
	0.0011	0.0000	0.0000	0.0000	0.0008	0.0000	0.0000	0.0000
Fortaleza (10)	1.6197***	0.5880***	0.7234***	0.9370	1.0821*	0.5983***	1.1508***	0.2751***
	(1.4184 - 1.8496)	(0.5180 - 0.6674)	(0.6324 - 0.8275)	(0.8477 - 1.0356)	(0.9941 - 1.1778)	(0.4678 - 0.7651)	(1.0390 - 1.2745)	(0.2267 - 0.3338)
	0.0000	0.0000	0.0000	0.2025	0.0683	0.0000	0.0070	0.0000
Natal (11)	1.1620*	0.8900	0.4670***	2.3979***	1.1168	0.4879***	1.4607***	0.1283***
	(0.9785 - 1.3800)	(0.7367 - 1.0753)	(0.3815 - 0.5717)	(1.9564 - 2.9389)	(0.9714 - 1.2840)	(0.3326 - 0.7159)	(1.2810 - 1.6656)	(0.0968 - 0.1700)
	0.0869	0.2272	0.0000	0.0000	0.1206	0.0002	0.0000	0.0000
Joao Pessoa (12)	1.0206	0.4952***	0.5581***	0.5708***	0.8749**	0.1799***	0.7762***	0.2237***
	(0.8881 - 1.1729)	(0.4214 - 0.5818)	(0.4723 - 0.6596)	(0.4733 - 0.6883)	(0.7836 - 0.9768)	(0.1307 - 0.2474)	(0.6966 - 0.8648)	(0.1793 - 0.2791)
	0.7741	0.0000	0.0000	0.0000	0.0174	0.0000	0.0000	0.0000
Recife (13)	1.5766***	0.7408***	0.7143***	1.2635***	1.3684***	0.3237***	1.3525***	0.2166***
	(1.3799 - 1.8013)	(0.6213 - 0.8832)	(0.5995 - 0.8511)	(1.0796 - 1.4786)	(1.2244 - 1.5294)	(0.2329 - 0.4498)	(1.2121 - 1.5091)	(0.1668 - 0.2813)
	0.0000	0.0008	0.0002	0.0036	0.0000	0.0000	0.0000	0.0000
Maceio (14)	0.8116*	0.7075**	0.5725***	0.9306	0.6341***	0.9755	0.7626***	0.4736***
	(0.6463 - 1.0193)	(0.5410 - 0.9252)	(0.4396 - 0.7456)	(0.6918 - 1.2517)	(0.5241 - 0.7671)	(0.5718 - 1.6641)	(0.6343 - 0.9168)	(0.3199 - 0.7012)
	0.0726	0.0115	0.0000	0.6343	0.0000	0.9274	0.0039	0.0002
Aracaju (15)	2.1987***	0.7482***	0.9892	1.2479*	1.6577***	0.1485***	1.9142***	0.0854***
	(1.8020 - 2.6828)	(0.6056 - 0.9245)	(0.7992 - 1.2243)	(0.9713 - 1.6034)	(1.4188 - 1.9369)	(0.0969 - 0.2276)	(1.6489 - 2.2222)	(0.0630 - 0.1159)
	0.0000	0.0072	0.9203	0.0832	0.0000	0.0000	0.0000	0.0000
Salvador (16)	2.2696***	0.9242	1.0504	1.4834***	1.7277***	0.1893***	1.6088***	0.5968***
	(2.0379 - 2.5276)	(0.8381 - 1.0191)	(0.9413 - 1.1721)	(1.3599 - 1.6180)	(1.6066 - 1.8580)	(0.1560 - 0.2298)	(1.4934 - 1.7330)	(0.5182 - 0.6873)
	0.0000	0.1141	0.3794	0.0000	0.0000	0.0000	0.0000	0.0000
Belo Horizonte (17)	1.7562***	0.7094***	0.9993	0.8929**	1.2973***	0.3370***	1.3631***	0.2980***

	(1.5540 - 1.9847)	(0.6311 - 0.7975)	(0.9074 - 1.1004)	(0.8025 - 0.9935)	(1.1734 - 1.4344)	(0.2774 - 0.4095)	(1.2406 - 1.4976)	(0.2609 - 0.3402)
	0.0000	0.0000	0.9882	0.0375	0.0000	0.0000	0.0000	0.0000
Vitoria (18)	0.8171***	0.5973***	0.8980**	0.5974***	0.9413	0.3742***	0.9059***	0.1782***
	(0.7522 - 0.8875)	(0.5335 - 0.6687)	(0.8100 - 0.9957)	(0.5501 - 0.6488)	(0.8715 - 1.0167)	(0.3159 - 0.4433)	(0.8411 - 0.9757)	(0.1529 - 0.2076)
	0.0000	0.0000	0.0411	0.0000	0.1237	0.0000	0.0090	0.0000
Rio de Janeiro (19)	1.8086***	0.5757***	0.5478***	1.0721***	1.2429***	0.3420***	1.1992***	0.2309***
	(1.6794 - 1.9478)	(0.5305 - 0.6247)	(0.5095 - 0.5891)	(1.0188 - 1.1283)	(1.1743 - 1.3155)	(0.3032 - 0.3858)	(1.1284 - 1.2744)	(0.2062 - 0.2585)
	0.0000	0.0000	0.0000	0.0075	0.0000	0.0000	0.0000	0.0000
São Paulo (20)	1.8538***	0.6243***	0.8455***	0.9372	1.2740***	0.3827***	1.2617***	0.2593***
	(1.6947 - 2.0278)	(0.5722 - 0.6811)	(0.7955 - 0.8985)	(0.8625 - 1.0185)	(1.1888 - 1.3654)	(0.3374 - 0.4342)	(1.1838 - 1.3447)	(0.2336 - 0.2877)
	0.0000	0.0000	0.0000	0.1263	0.0000	0.0000	0.0000	0.0000
Curitiba (21)	1.8203***	0.5639***	0.9517	0.7415***	2.4956***	0.2780***	1.2488***	0.2210***
	(1.5230 - 2.1757)	(0.4843 - 0.6566)	(0.8458 - 1.0708)	(0.6029 - 0.9119)	(2.1822 - 2.8541)	(0.2098 - 0.3684)	(1.1025 - 1.4144)	(0.1832 - 0.2667)
	0.0000	0.0000	0.4104	0.0046	0.0000	0.0000	0.0005	0.0000
Florianopolis (22)	1.7133***	0.5720***	0.9796	0.6026***	1.1544**	0.3546***	1.4196***	0.1333***
	(1.3817 - 2.1245)	(0.4902 - 0.6674)	(0.8638 - 1.1108)	(0.4886 - 0.7432)	(1.0065 - 1.3239)	(0.2607 - 0.4823)	(1.2327 - 1.6349)	(0.1116 - 0.1591)
	0.0000	0.0000	0.7479	0.0000	0.0401	0.0000	0.0000	0.0000
Porto Alegre (23)	1.1000	0.5749***	0.5087***	0.9002	1.0198	0.2986***	1.0373	0.2079***
	(0.9271 - 1.3052)	(0.4931 - 0.6704)	(0.4548 - 0.5691)	(0.7395 - 1.0959)	(0.9037 - 1.1508)	(0.2280 - 0.3912)	(0.9101 - 1.1823)	(0.1763 - 0.2452)
	0.2748	0.0000	0.0000	0.2948	0.7506	0.0000	0.5830	0.0000
Campo Grande (24)	1.4461***	0.6528***	1.0061	0.7580***	0.9307***	0.5233***	0.9409**	0.3641***
	(1.3753 - 1.5206)	(0.6177 - 0.6899)	(0.9634 - 1.0506)	(0.7342 - 0.7825)	(0.8833 - 0.9806)	(0.4854 - 0.5641)	(0.8923 - 0.9921)	(0.3407 - 0.3891)
	0.0000	0.0000	0.7844	0.0000	0.0070	0.0000	0.0243	0.0000
Cuiaba (25)	2.6556***	0.4438***	0.7553***	1.3661***	1.1543**	0.5107***	1.0207	0.4532***
	(2.1950 - 3.2128)	(0.3841 - 0.5129)	(0.6690 - 0.8528)	(1.1549 - 1.6160)	(1.0143 - 1.3136)	(0.3835 - 0.6802)	(0.8874 - 1.1741)	(0.3770 - 0.5447)
	0.0000	0.0000	0.0000	0.0003	0.0296	0.0000	0.7739	0.0000
Goiania (26)	1.3768***	0.2902***	0.5035***	0.5963***	0.7553***	0.1271***	0.8995*	0.1078***
	(1.1776 - 1.6097)	(0.2537 - 0.3321)	(0.4467 - 0.5676)	(0.5011 - 0.7095)	(0.6683 - 0.8536)	(0.0972 - 0.1661)	(0.7957 - 1.0170)	(0.0900 - 0.1292)
	0.0001	0.0000	0.0000	0.0000	0.0000	0.0000	0.0908	0.0000
Brasilia (27)	0.8944*	0.6868***	0.6534***	0.9201	0.7953***	0.4873***	0.9526	0.3670***
	(0.7990 - 1.0012)	(0.6059 - 0.7786)	(0.5742 - 0.7435)	(0.8281 - 1.0224)	(0.7340 - 0.8617)	(0.3856 - 0.6158)	(0.8691 - 1.0441)	(0.3086 - 0.4364)
	0.0524	0.0000	0.0000	0.1215	0.0000	0.0000	0.2996	0.0000
Month								

1	omitted							
2	0.8267***	0.4015***	0.3398***	0.6712***	0.4937***	0.3698***	0.5215***	0.6425***
	(0.7287 - 0.9379)	(0.3677 - 0.4383)	(0.3022 - 0.3820)	(0.6035 - 0.7463)	(0.4568 - 0.5335)	(0.3159 - 0.4329)	(0.4851 - 0.5607)	(0.5447 - 0.7578)
	0.0031	0.0000	0.0000	0.0000	0.0000	0.0000	0.0000	0.0000
3	1.1497	0.3688***	0.3507***	0.8889	0.6316***	0.5280***	0.5761***	0.6167***
	(0.9599 - 1.3772)	(0.3176 - 0.4282)	(0.2975 - 0.4133)	(0.7684 - 1.0284)	(0.5561 - 0.7174)	(0.4468 - 0.6240)	(0.4990 - 0.6652)	(0.5365 - 0.7087)
	0.1297	0.0000	0.0000	0.1134	0.0000	0.0000	0.0000	0.0000
4	1.5874***	0.3973***	0.7742***	0.6489***	0.7012***	0.6073***	0.6901***	0.8206**
	(1.3375 - 1.8840)	(0.3621 - 0.4358)	(0.6790 - 0.8828)	(0.5563 - 0.7570)	(0.6252 - 0.7865)	(0.5247 - 0.7030)	(0.6120 - 0.7782)	(0.6925 - 0.9723)
	0.0000	0.0000	0.0001	0.0000	0.0000	0.0000	0.0000	0.0224
5	1.0962	0.3669***	0.6162***	0.6202***	0.5875***	0.3281***	0.5498***	0.7183***
	(0.9353 - 1.2848)	(0.3356 - 0.4011)	(0.5590 - 0.6792)	(0.5362 - 0.7174)	(0.5349 - 0.6453)	(0.2836 - 0.3796)	(0.4941 - 0.6119)	(0.5925 - 0.8709)
	0.2567	0.0000	0.0000	0.0000	0.0000	0.0000	0.0000	0.0008
6	1.1394**	0.4647***	0.9044	0.4874***	0.5553***	0.8297	0.6790***	0.6084***
	(1.0151 - 1.2789)	(0.4016 - 0.5377)	(0.7787 - 1.0503)	(0.4139 - 0.5741)	(0.5095 - 0.6053)	(0.6457 - 1.0662)	(0.6052 - 0.7617)	(0.4938 - 0.7495)
	0.0268	0.0000	0.1878	0.0000	0.0000	0.1445	0.0000	0.0000
7	1.5228***	0.5846***	0.6115***	1.0333	0.8242***	0.3627***	0.8289***	0.6689***
	(1.3072 - 1.7740)	(0.5298 - 0.6449)	(0.5120 - 0.7304)	(0.8816 - 1.2110)	(0.7517 - 0.9037)	(0.2894 - 0.4544)	(0.7437 - 0.9239)	(0.5469 - 0.8179)
	0.0000	0.0000	0.0000	0.6862	0.0000	0.0000	0.0007	0.0001
8	1.3346***	0.3311***	0.4461***	0.8711**	0.6581***	0.3144***	0.5690***	0.6807***
	(1.1626 - 1.5321)	(0.2872 - 0.3817)	(0.3689 - 0.5394)	(0.7622 - 0.9955)	(0.5958 - 0.7269)	(0.2380 - 0.4153)	(0.5139 - 0.6300)	(0.5482 - 0.8453)
	0.0000	0.0000	0.0000	0.0427	0.0000	0.0000	0.0000	0.0005
9	1.0035	0.4933***	0.7025***	0.6841***	0.6642***	0.6504**	0.6483***	0.6225***
	(0.8327 - 1.2094)	(0.4187 - 0.5812)	(0.5816 - 0.8484)	(0.5458 - 0.8574)	(0.6021 - 0.7328)	(0.4680 - 0.9038)	(0.5597 - 0.7508)	(0.4925 - 0.7869)
	0.9706	0.0000	0.0002	0.0010	0.0000	0.0104	0.0000	0.0001
10	1.1599	0.5381***	0.3614***	1.2355*	0.7067***	0.7714	0.7267***	0.5866***
	(0.8746 - 1.5382)	(0.4349 - 0.6659)	(0.2874 - 0.4544)	(0.9706 - 1.5727)	(0.6134 - 0.8143)	(0.5337 - 1.1150)	(0.6134 - 0.8610)	(0.3951 - 0.8709)
	0.3032	0.0000	0.0000	0.0859	0.0000	0.1673	0.0002	0.0082
11	1.0958	0.5494***	0.5696***	0.9419	0.5836***	0.9584	0.7361***	0.4395***
	(0.8189 - 1.4664)	(0.4478 - 0.6740)	(0.4637 - 0.6997)	(0.7365 - 1.2046)	(0.4949 - 0.6883)	(0.6547 - 1.4032)	(0.6229 - 0.8698)	(0.3023 - 0.6389)
	0.5382	0.0000	0.0000	0.6333	0.0000	0.8272	0.0003	0.0000
12	0.8365**	0.4519***	0.5080***	0.6293***	0.5237***	0.6279***	0.4601***	0.8399
	(0.7045 - 0.9933)	(0.3852 - 0.5302)	(0.4289 - 0.6018)	(0.5212 - 0.7599)	(0.4656 - 0.5891)	(0.4538 - 0.8688)	(0.4058 - 0.5216)	(0.6282 - 1.1228)

	0.0417	0.0000	0.0000	0.0000	0.0000	0.0050	0.0000	0.2388
Year								
2012					omitted			
2013	0.9597	1.0611	0.5689***	1.8189***	1.1364**	0.7195**	1.1994***	0.5917***
	(0.7769 - 1.1856)	(0.8988 - 1.2528)	(0.4688 - 0.6903)	(1.5782 - 2.0964)	(1.0127 - 1.2752)	(0.5503 - 0.9407)	(1.0471 - 1.3740)	(0.4322 - 0.8099)
	0.7030	0.4837	0.0000	0.0000	0.0297	0.0161	0.0087	0.0011
2014	1.7726***	1.0031	0.9543	1.7386***	1.5348***	0.8780	1.7887***	0.3984***
	(1.4461 - 2.1728)	(0.8670 - 1.1606)	(0.8051 - 1.1312)	(1.5343 - 1.9702)	(1.3484 - 1.7470)	(0.6656 - 1.1581)	(1.5741 - 2.0325)	(0.3011 - 0.5272)
	0.0000	0.9666	0.5899	0.0000	0.0000	0.3570	0.0000	0.0000
2015	1.6908***	1.3819***	0.9782	1.9742***	1.8773***	1.0832	1.9966***	0.5774***
	(1.3223 - 2.1620)	(1.1677 - 1.6353)	(0.8053 - 1.1882)	(1.6891 - 2.3073)	(1.6613 - 2.1214)	(0.8546 - 1.3731)	(1.7500 - 2.2778)	(0.4200 - 0.7939)
	0.0000	0.0002	0.8243	0.0000	0.0000	0.5087	0.0000	0.0007
2016	1.7102***	1.4031***	0.8873	2.2448***	1.8546***	0.8120	1.8798***	0.5953***
	(1.2375 - 2.3634)	(1.1461 - 1.7177)	(0.7387 - 1.0658)	(1.6927 - 2.9769)	(1.5866 - 2.1679)	(0.5562 - 1.1854)	(1.5610 - 2.2637)	(0.4280 - 0.8279)
	0.0012	0.0010	0.2012	0.0000	0.0000	0.2807	0.0000	0.0021
Constant	0.0047***	0.0194***	0.0091***	0.0115***	0.0112***	0.0312***	0.0090***	0.0471***
	(0.0033 - 0.0068)	(0.0154 - 0.0244)	(0.0069 - 0.0120)	(0.0087 - 0.0151)	(0.0090 - 0.0139)	(0.0207 - 0.0472)	(0.0074 - 0.0108)	(0.0343 - 0.0646)
	0.0000	0.0000	0.0000	0.0000	0.0000	0.0000	0.0000	0.0000
Observations	975,113	1,619,110	1,339,859	1,236,633	1,550,041	910,334	1,903,439	667,077

Note: coefficients of interaction terms are not reported. *** p<0.01, ** p<0.05, * p<0.1
