# Supplementary material for: In utero exposure to economic fluctuations and birth outcomes: An analysis of the relevance of the local unemployment rate in Brazilian state capitals
Source: PLoS One. 2019 Oct 10;14(10):e0223673. doi: 10.1371/journal.pone.0223673 (PMC6786569; doi:10.1371/journal.pone.0223673)
Supplement: S4 Table — (PDF) [file pone.0223673.s004.pdf]

## S4 Table. Extended version of table 5.

| PANEL A | Age | | Marital status | | Race/ethnicity | | Years of education | |
|---|---|---|---|---|---|---|---|---|
| | (1) | (2) | (3) | (4) | (5) | (6) | (7) | (8) |
| | ≤ 24 years | ≥ 25 years | Partner | No Partner | Black or Brown | White | 11 or less | 12 or more |
| Unemployment rate months 10 to 18 before birth | 0.9974 | 1.0026 | 0.9923 | 1.0078 | 1.0171 | 0.9869 | 0.9994 | 1.0006 |
| 95% CI | (0.9873 - 1.0077) | (0.9924 - 1.0129) | (0.9676 - 1.0175) | (0.9828 - 1.0334) | (0.9931 - 1.0418) | (0.9644 - 1.0099) | (0.9849 - 1.0141) | (0.9861 - 1.0154) |
| p-val | 0.6203 | 0.6203 | 0.5449 | 0.5449 | 0.1646 | 0.2627 | 0.9314 | 0.9314 |
| City of residence (code) | | | | | | | | |
| Porto Velho (1) | | | | | omitted | | | |
| Rio Branco (2) | 0.9771 | 1.0234 | 1.7894*** | 0.5588*** | 1.3471*** | 0.7826*** | 0.8175*** | 1.2232*** |
| | (0.9467 - 1.0085) | (0.9916 - 1.0563) | (1.6553 - 1.9344) | (0.5170 - 0.6041) | (1.2465 - 1.4559) | (0.7263 - 0.8434) | (0.7805 - 0.8563) | (1.1678 - 1.2812) |
| | 0.1506 | 0.1506 | 0.0000 | 0.0000 | 0.0000 | 0.0000 | 0.0000 | 0.0000 |
| Manaus (3) | 0.9683 | 1.0327 | 0.2627*** | 3.8071*** | 1.5045*** | 0.6996*** | 1.2901*** | 0.7751*** |
| | (0.9214 - 1.0176) | (0.9827 - 1.0854) | (0.2319 - 0.2975) | (3.3615 - 4.3119) | (1.3337 - 1.6972) | (0.6226 - 0.7861) | (1.2004 - 1.3865) | (0.7212 - 0.8331) |
| | 0.2038 | 0.2038 | 0.0000 | 0.0000 | 0.0000 | 0.0000 | 0.0000 | 0.0000 |
| Boa Vista (4) | 0.9681*** | 1.0330*** | 0.8305*** | 1.2041*** | 1.3572*** | 0.3943*** | 0.8577*** | 1.1659*** |
| | (0.9609 - 0.9753) | (1.0253 - 1.0407) | (0.8137 - 0.8476) | (1.1798 - 1.2289) | (1.3317 - 1.3831) | (0.3872 - 0.4015) | (0.8472 - 0.8683) | (1.1517 - 1.1803) |
| | 0.0000 | 0.0000 | 0.0000 | 0.0000 | 0.0000 | 0.0000 | 0.0000 | 0.0000 |
| Belem (5) | 0.9363*** | 1.0681*** | 0.7971*** | 1.2545*** | 1.2529*** | 0.8304*** | 1.0570 | 0.9461 |
| | (0.8926 - 0.9820) | (1.0183 - 1.1203) | (0.7081 - 0.8973) | (1.1144 - 1.4123) | (1.1195 - 1.4021) | (0.7450 - 0.9255) | (0.9857 - 1.1335) | (0.8822 - 1.0145) |
| | 0.0068 | 0.0068 | 0.0002 | 0.0002 | 0.0001 | 0.0008 | 0.1198 | 0.1198 |
| Macapa (6) | 1.0915*** | 0.9162*** | 2.3208*** | 0.4309*** | 1.7589*** | 0.5722*** | 1.2154*** | 0.8228*** |
| | (1.0420 - 1.1434) | (0.8746 - 0.9597) | (2.0686 - 2.6037) | (0.3841 - 0.4834) | (1.5787 - 1.9597) | (0.5157 - 0.6349) | (1.1361 - 1.3002) | (0.7691 - 0.8802) |
| | 0.0002 | 0.0002 | 0.0000 | 0.0000 | 0.0000 | 0.0000 | 0.0000 | 0.0000 |
| Palmas (7) | 0.5999*** | 1.6669*** | 2.1130*** | 0.4733*** | 0.6949*** | 1.3756*** | 0.4406*** | 2.2694*** |
| | (0.5872 - 0.6129) | (1.6317 - 1.7029) | (2.0039 - 2.2280) | (0.4488 - 0.4990) | (0.6602 - 0.7314) | (1.3102 - 1.4442) | (0.4266 - 0.4552) | (2.1969 - 2.3443) |
| | 0.0000 | 0.0000 | 0.0000 | 0.0000 | 0.0000 | 0.0000 | 0.0000 | 0.0000 |
| São Luis (8) | 0.7688*** | 1.3007*** | 1.1572* | 0.8642* | 1.0241 | 1.0269 | 0.9536 | 1.0487 |
| | (0.7169 - 0.8244) | (1.2130 - 1.3948) | (0.9752 - 1.3730) | (0.7283 - 1.0254) | (0.8688 - 1.2070) | (0.8767 - 1.2028) | (0.8620 - 1.0549) | (0.9479 - 1.1601) |
| | 0.0000 | 0.0000 | 0.0944 | 0.0944 | 0.7768 | 0.7423 | 0.3565 | 0.3565 |
| Teresina (9) | 0.5894*** | 1.6966*** | 1.8525*** | 0.5398*** | 0.7889*** | 1.2970*** | 0.7346*** | 1.3613*** |
| | (0.5766 - 0.6025) | (1.6597 - 1.7344) | (1.7532 - 1.9573) | (0.5109 - 0.5704) | (0.7481 - 0.8319) | (1.2322 - 1.3651) | (0.7105 - 0.7595) | (1.3167 - 1.4074) |
| | 0.0000 | 0.0000 | 0.0000 | 0.0000 | 0.0000 | 0.0000 | 0.0000 | 0.0000 |

| | | | | | | | | |
|---|---|---|---|---|---|---|---|---|
| Fortaleza (10) | 0.6925*** | 1.4441*** | 0.8142*** | 1.2282*** | 1.4800*** | 0.7328*** | 0.8807*** | 1.1354*** |
| | (0.6825 - 0.7026) | (1.4234 - 1.4652) | (0.7856 - 0.8439) | (1.1850 - 1.2729) | (1.4255 - 1.5365) | (0.7065 - 0.7601) | (0.8618 - 0.9001) | (1.1110 - 1.1603) |
| | 0.0000 | 0.0000 | 0.0000 | 0.0000 | 0.0000 | 0.0000 | 0.0000 | 0.0000 |
| Natal (11) | 0.6699*** | 1.4927*** | 1.3599*** | 0.7354*** | 0.3716*** | 2.9075*** | 0.7633*** | 1.3100*** |
| | (0.6443 - 0.6966) | (1.4356 - 1.5520) | (1.2335 - 1.4991) | (0.6670 - 0.8107) | (0.3386 - 0.4079) | (2.6581 - 3.1802) | (0.7208 - 0.8084) | (1.2370 - 1.3874) |
| | 0.0000 | 0.0000 | 0.0000 | 0.0000 | 0.0000 | 0.0000 | 0.0000 | 0.0000 |
| Joao Pessoa (12) | 0.6419*** | 1.5578*** | 3.7670*** | 0.2655*** | 0.5402*** | 1.9583*** | 0.6579*** | 1.5201*** |
| | (0.6217 - 0.6628) | (1.5087 - 1.6085) | (3.4768 - 4.0813) | (0.2450 - 0.2876) | (0.5005 - 0.5830) | (1.8196 - 2.1076) | (0.6275 - 0.6897) | (1.4498 - 1.5938) |
| | 0.0000 | 0.0000 | 0.0000 | 0.0000 | 0.0000 | 0.0000 | 0.0000 | 0.0000 |
| Recife (13) | 0.6323*** | 1.5815*** | 0.5641*** | 1.7727*** | 0.4501*** | 2.3881*** | 0.6335*** | 1.5785*** |
| | (0.6079 - 0.6577) | (1.5205 - 1.6450) | (0.5115 - 0.6221) | (1.6075 - 1.9549) | (0.4097 - 0.4945) | (2.1810 - 2.6148) | (0.5978 - 0.6714) | (1.4894 - 1.6728) |
| | 0.0000 | 0.0000 | 0.0000 | 0.0000 | 0.0000 | 0.0000 | 0.0000 | 0.0000 |
| Maceio (14) | 0.9611 | 1.0405 | 0.8450** | 1.1835** | 1.4075*** | 0.7588*** | 0.9959 | 1.0041 |
| | (0.9074 - 1.0179) | (0.9824 - 1.1021) | (0.7329 - 0.9742) | (1.0265 - 1.3645) | (1.2287 - 1.6124) | (0.6657 - 0.8649) | (0.9163 - 1.0825) | (0.9238 - 1.0914) |
| | 0.1758 | 0.1758 | 0.0204 | 0.0204 | 0.0000 | 0.0000 | 0.9232 | 0.9232 |
| Aracaju (15) | 0.6255*** | 1.5988*** | 1.7643*** | 0.5668*** | 0.7959*** | 1.1495** | 0.6849*** | 1.4600*** |
| | (0.5962 - 0.6562) | (1.5240 - 1.6773) | (1.5666 - 1.9869) | (0.5033 - 0.6383) | (0.7106 - 0.8914) | (1.0306 - 1.2822) | (0.6386 - 0.7346) | (1.3614 - 1.5659) |
| | 0.0000 | 0.0000 | 0.0000 | 0.0000 | 0.0001 | 0.0124 | 0.0000 | 0.0000 |
| Salvador (16) | 0.5218*** | 1.9163*** | 0.4730*** | 2.1142*** | 1.4927*** | 0.6919*** | 0.7996*** | 1.2507*** |
| | (0.5115 - 0.5324) | (1.8784 - 1.9549) | (0.4499 - 0.4973) | (2.0109 - 2.2229) | (1.4222 - 1.5666) | (0.6604 - 0.7250) | (0.7760 - 0.8239) | (1.2138 - 1.2887) |
| | 0.0000 | 0.0000 | 0.0000 | 0.0000 | 0.0000 | 0.0000 | 0.0000 | 0.0000 |
| Belo Horizonte (17) | 0.4325*** | 2.3119*** | 0.5264*** | 1.8996*** | 0.3526*** | 2.9360*** | 0.5117*** | 1.9543*** |
| | (0.4287 - 0.4364) | (2.2915 - 2.3325) | (0.5155 - 0.5376) | (1.8601 - 1.9400) | (0.3459 - 0.3595) | (2.8824 - 2.9905) | (0.5058 - 0.5176) | (1.9318 - 1.9771) |
| | 0.0000 | 0.0000 | 0.0000 | 0.0000 | 0.0000 | 0.0000 | 0.0000 | 0.0000 |
| Vitoria (18) | 0.4803*** | 2.0819*** | 0.4400*** | 2.2727*** | 0.4627*** | 2.3635*** | 0.4192*** | 2.3853*** |
| | (0.4732 - 0.4876) | (2.0508 - 2.1135) | (0.4237 - 0.4569) | (2.1885 - 2.3603) | (0.4458 - 0.4801) | (2.2800 - 2.4500) | (0.4098 - 0.4289) | (2.3314 - 2.4405) |
| | 0.0000 | 0.0000 | 0.0000 | 0.0000 | 0.0000 | 0.0000 | 0.0000 | 0.0000 |
| Rio de Janeiro (19) | 0.6312*** | 1.5843*** | 0.2805*** | 3.5650*** | 0.3102*** | 3.4674*** | 0.7244*** | 1.3805*** |
| | (0.6282 - 0.6342) | (1.5768 - 1.5918) | (0.2771 - 0.2839) | (3.5218 - 3.6087) | (0.3063 - 0.3142) | (3.4247 - 3.5106) | (0.7184 - 0.7304) | (1.3692 - 1.3919) |
| | 0.0000 | 0.0000 | 0.0000 | 0.0000 | 0.0000 | 0.0000 | 0.0000 | 0.0000 |
| São Paulo (20) | 0.5329*** | 1.8764*** | 0.6432*** | 1.5546*** | 0.1675*** | 6.1533*** | 0.6387*** | 1.5656*** |
| | (0.5287 - 0.5372) | (1.8615 - 1.8914) | (0.6314 - 0.6553) | (1.5259 - 1.5839) | (0.1646 - 0.1704) | (6.0532 - 6.2550) | (0.6323 - 0.6452) | (1.5500 - 1.5814) |
| | 0.0000 | 0.0000 | 0.0000 | 0.0000 | 0.0000 | 0.0000 | 0.0000 | 0.0000 |

| | | | | | | | | |
|---|---|---|---|---|---|---|---|---|
| Curitiba (21) | 0.5329*** | 1.8766*** | 0.4989*** | 2.0043*** | 0.0364*** | 28.6516*** | 0.4507*** | 2.2187*** |
| | (0.5195 - 0.5466) | (1.8295 - 1.9250) | (0.4689 - 0.5309) | (1.8835 - 2.1329) | (0.0343 - 0.0386) | (27.0881 - 30.3053) | (0.4351 - 0.4669) | (2.1418 - 2.2983) |
| | 0.0000 | 0.0000 | 0.0000 | 0.0000 | 0.0000 | 0.0000 | 0.0000 | 0.0000 |
| Florianopolis (22) | 0.5089*** | 1.9650*** | 1.1966*** | 0.8357*** | 0.0306*** | 35.8598*** | 0.4080*** | 2.4512*** |
| | (0.4919 - 0.5264) | (1.8996 - 2.0328) | (1.1020 - 1.2993) | (0.7696 - 0.9074) | (0.0283 - 0.0330) | (33.2981 - 38.6185) | (0.3892 - 0.4277) | (2.3382 - 2.5697) |
| | 0.0000 | 0.0000 | 0.0000 | 0.0000 | 0.0000 | 0.0000 | 0.0000 | 0.0000 |
| Porto Alegre (23) | 0.5600*** | 1.7858*** | 0.4632*** | 2.1590*** | 0.0681*** | 16.0171*** | 0.5794*** | 1.7258*** |
| | (0.5475 - 0.5727) | (1.7461 - 1.8264) | (0.4388 - 0.4889) | (2.0452 - 2.2791) | (0.0647 - 0.0716) | (15.2533 - 16.8192) | (0.5619 - 0.5975) | (1.6735 - 1.7797) |
| | 0.0000 | 0.0000 | 0.0000 | 0.0000 | 0.0000 | 0.0000 | 0.0000 | 0.0000 |
| Campo Grande (24) | 0.7663*** | 1.3049*** | 0.3847*** | 2.5991*** | 0.3399*** | 3.1003*** | 0.6305*** | 1.5861*** |
| | (0.7598 - 0.7729) | (1.2938 - 1.3161) | (0.3773 - 0.3924) | (2.5486 - 2.6506) | (0.3337 - 0.3463) | (3.0457 - 3.1559) | (0.6237 - 0.6374) | (1.5689 - 1.6034) |
| | 0.0000 | 0.0000 | 0.0000 | 0.0000 | 0.0000 | 0.0000 | 0.0000 | 0.0000 |
| Cuiaba (25) | 0.6886*** | 1.4523*** | 1.9273*** | 0.5189*** | 0.6289*** | 1.6811*** | 0.5533*** | 1.8072*** |
| | (0.6807 - 0.6966) | (1.4356 - 1.4691) | (1.8725 - 1.9836) | (0.5041 - 0.5340) | (0.6130 - 0.6453) | (1.6402 - 1.7230) | (0.5449 - 0.5619) | (1.7796 - 1.8352) |
| | 0.0000 | 0.0000 | 0.0000 | 0.0000 | 0.0000 | 0.0000 | 0.0000 | 0.0000 |
| Goiania (26) | 0.5857*** | 1.7073*** | 1.5130*** | 0.6609*** | 0.2777*** | 3.8822*** | 0.5226*** | 1.9134*** |
| | (0.5700 - 0.6019) | (1.6614 - 1.7544) | (1.4155 - 1.6173) | (0.6183 - 0.7065) | (0.2608 - 0.2957) | (3.6559 - 4.1226) | (0.5033 - 0.5427) | (1.8427 - 1.9869) |
| | 0.0000 | 0.0000 | 0.0000 | 0.0000 | 0.0000 | 0.0000 | 0.0000 | 0.0000 |
| Brasilia (27) | 0.5418*** | 1.8457*** | 0.7278*** | 1.3740*** | 0.4628*** | 2.2990*** | 0.5279*** | 1.8944*** |
| | (0.5326 - 0.5512) | (1.8142 - 1.8777) | (0.6968 - 0.7601) | (1.3156 - 1.4351) | (0.4436 - 0.4828) | (2.2071 - 2.3947) | (0.5142 - 0.5419) | (1.8455 - 1.9446) |
| | 0.0000 | 0.0000 | 0.0000 | 0.0000 | 0.0000 | 0.0000 | 0.0000 | 0.0000 |
| Month | | | | | | | | |
| 1 | | | | | omitted | | | |
| 2 | 0.9743* | 1.0263* | 0.9569*** | 1.0450*** | 1.1285*** | 0.9433*** | 1.2070*** | 0.8285*** |
| | (0.9489 - 1.0005) | (0.9995 - 1.0539) | (0.9268 - 0.9881) | (1.0121 - 1.0790) | (1.1058 - 1.1518) | (0.9252 - 0.9617) | (1.1841 - 1.2304) | (0.8127 - 0.8445) |
| | 0.0542 | 0.0542 | 0.0070 | 0.0070 | 0.0000 | 0.0000 | 0.0000 | 0.0000 |
| 3 | 0.9155*** | 1.0923*** | 1.0372* | 0.9641* | 0.9251*** | 1.1054*** | 1.0211* | 0.9794* |
| | (0.8930 - 0.9385) | (1.0655 - 1.1199) | (0.9981 - 1.0779) | (0.9277 - 1.0019) | (0.9048 - 0.9457) | (1.0814 - 1.1298) | (0.9989 - 1.0437) | (0.9581 - 1.0011) |
| | 0.0000 | 0.0000 | 0.0627 | 0.0627 | 0.0000 | 0.0000 | 0.0625 | 0.0625 |
| 4 | 0.9435*** | 1.0599*** | 0.9133*** | 1.0949*** | 0.8933*** | 1.1645*** | 0.9667*** | 1.0345*** |
| | (0.9235 - 0.9639) | (1.0374 - 1.0828) | (0.8804 - 0.9474) | (1.0555 - 1.1359) | (0.8651 - 0.9223) | (1.1289 - 1.2012) | (0.9504 - 0.9832) | (1.0171 - 1.0522) |
| | 0.0000 | 0.0000 | 0.0000 | 0.0000 | 0.0000 | 0.0000 | 0.0001 | 0.0001 |
| 5 | 0.9989 | 1.0011 | 0.9710 | 1.0299 | 1.0137 | 1.0180 | 0.9613*** | 1.0402*** |

| | | | | | | | | |
|---|---|---|---|---|---|---|---|---|
| | (0.9771 - 1.0212) | (0.9793 - 1.0234) | (0.9297 - 1.0142) | (0.9860 - 1.0757) | (0.9722 - 1.0571) | (0.9738 - 1.0643) | (0.9352 - 0.9881) | (1.0120 - 1.0693) |
| | 0.9227 | 0.9227 | 0.1849 | 0.1849 | 0.5231 | 0.4312 | 0.0049 | 0.0049 |
| 6 | 0.9270*** | 1.0788*** | 1.0344 | 0.9668 | 1.2448*** | 0.8288*** | 0.9955 | 1.0045 |
| | (0.9108 - 0.9433) | (1.0601 - 1.0979) | (0.9884 - 1.0824) | (0.9239 - 1.0117) | (1.1929 - 1.2989) | (0.7948 - 0.8642) | (0.9720 - 1.0196) | (0.9808 - 1.0288) |
| | 0.0000 | 0.0000 | 0.1449 | 0.1449 | 0.0000 | 0.0000 | 0.7108 | 0.7108 |
| 7 | 1.0338** | 0.9673** | 0.9516 | 1.0508 | 1.1162*** | 0.9408** | 1.0835*** | 0.9229*** |
| | (1.0027 - 1.0660) | (0.9381 - 0.9973) | (0.8696 - 1.0414) | (0.9602 - 1.1500) | (1.0523 - 1.1840) | (0.8910 - 0.9934) | (1.0303 - 1.1395) | (0.8776 - 0.9706) |
| | 0.0330 | 0.0330 | 0.2813 | 0.2813 | 0.0003 | 0.0280 | 0.0018 | 0.0018 |
| 8 | 1.0411*** | 0.9605*** | 0.9114* | 1.0972* | 1.0218 | 1.0209 | 1.1128*** | 0.8986*** |
| | (1.0137 - 1.0692) | (0.9353 - 0.9865) | (0.8219 - 1.0107) | (0.9894 - 1.2168) | (0.9580 - 1.0898) | (0.9601 - 1.0856) | (1.0581 - 1.1703) | (0.8545 - 0.9451) |
| | 0.0031 | 0.0031 | 0.0786 | 0.0786 | 0.5129 | 0.5086 | 0.0000 | 0.0000 |
| 9 | 1.0453** | 0.9566** | 0.9915 | 1.0086 | 1.0060 | 1.0097 | 1.0191 | 0.9812 |
| | (1.0076 - 1.0844) | (0.9221 - 0.9924) | (0.9009 - 1.0912) | (0.9164 - 1.1100) | (0.9302 - 1.0880) | (0.9321 - 1.0938) | (0.9710 - 1.0697) | (0.9349 - 1.0299) |
| | 0.0180 | 0.0180 | 0.8611 | 0.8611 | 0.8803 | 0.8129 | 0.4433 | 0.4433 |
| 10 | 0.9365*** | 1.0678*** | 0.9725 | 1.0283 | 0.9334** | 1.1082*** | 1.0158 | 0.9844 |
| | (0.9066 - 0.9675) | (1.0336 - 1.1030) | (0.8932 - 1.0588) | (0.9445 - 1.1196) | (0.8713 - 1.0000) | (1.0373 - 1.1840) | (0.9764 - 1.0568) | (0.9463 - 1.0241) |
| | 0.0001 | 0.0001 | 0.5202 | 0.5202 | 0.0500 | 0.0023 | 0.4369 | 0.4369 |
| 11 | 1.0126 | 0.9875 | 0.9204* | 1.0865* | 1.0494 | 0.9803 | 0.9920 | 1.0081 |
| | (0.9783 - 1.0481) | (0.9541 - 1.0221) | (0.8429 - 1.0050) | (0.9950 - 1.1864) | (0.9794 - 1.1244) | (0.9162 - 1.0488) | (0.9506 - 1.0351) | (0.9661 - 1.0519) |
| | 0.4753 | 0.4753 | 0.0644 | 0.0644 | 0.1711 | 0.5633 | 0.7101 | 0.7101 |
| 12 | 0.9811 | 1.0193 | 0.8723*** | 1.1464*** | 1.0312 | 0.9981 | 1.0692*** | 0.9353*** |
| | (0.9525 - 1.0105) | (0.9896 - 1.0499) | (0.8093 - 0.9402) | (1.0636 - 1.2357) | (0.9736 - 1.0923) | (0.9465 - 1.0525) | (1.0340 - 1.1056) | (0.9045 - 0.9671) |
| | 0.2052 | 0.2052 | 0.0004 | 0.0004 | 0.2942 | 0.9427 | 0.0001 | 0.0001 |
| Year | | | | | | | | |
| 2013 | | | | | omitted | | | |
| 2014 | 1.0349*** | 0.9663*** | 1.0534 | 0.9493 | 1.0435* | 0.9582* | 1.2210*** | 0.8190*** |
| | (1.0097 - 1.0607) | (0.9428 - 0.9904) | (0.9785 - 1.1340) | (0.8818 - 1.0220) | (0.9922 - 1.0974) | (0.9148 - 1.0036) | (1.1834 - 1.2598) | (0.7938 - 0.8451) |
| | 0.0064 | 0.0064 | 0.1671 | 0.1671 | 0.0980 | 0.0707 | 0.0000 | 0.0000 |
| 2015 | 1.0370* | 0.9643* | 0.8678*** | 1.1524*** | 0.8078*** | 1.2448*** | 0.9351*** | 1.0694*** |
| | (0.9963 - 1.0794) | (0.9264 - 1.0037) | (0.8038 - 0.9369) | (1.0674 - 1.2441) | (0.7709 - 0.8463) | (1.1918 - 1.3000) | (0.9042 - 0.9672) | (1.0339 - 1.1060) |
| | 0.0752 | 0.0752 | 0.0003 | 0.0003 | 0.0000 | 0.0000 | 0.0001 | 0.0001 |
| 2016 | 0.9885 | 1.0116 | 0.7609*** | 1.3142*** | 0.8969*** | 1.1182*** | 0.7687*** | 1.3009*** |
| | (0.9713 - 1.0060) | (0.9940 - 1.0296) | (0.7380 - 0.7845) | (1.2746 - 1.3551) | (0.8693 - 0.9253) | (1.0859 - 1.1515) | (0.7504 - 0.7874) | (1.2700 - 1.3327) |

| | | | | | | | | |
|---|---|---|---|---|---|---|---|---|
| | 0.1973 | 0.1973 | 0.0000 | 0.0000 | 0.0000 | 0.0000 | 0.0000 | 0.0000 |
| Constant | 0.9782 | 1.0223 | 2.1625*** | 0.4624*** | 4.8054*** | 0.1860*** | 4.3157*** | 0.2317*** |
| | (0.9216 - 1.0383) | (0.9632 - 1.0851) | (1.9292 - 2.4240) | (0.4125 - 0.5183) | (4.2735 - 5.4035) | (0.1662 - 0.2081) | (4.0082 - 4.6468) | (0.2152 - 0.2495) |
| | 0.4683 | 0.4683 | 0.0000 | 0.0000 | 0.0000 | 0.0000 | 0.0000 | 0.0000 |
| Observations | 2,427,273 | 2,427,273 | 2,408,605 | 2,408,605 | 2,289,931 | 2,289,931 | 2,404,774 | 2,404,774 |

| **PANEL B** | Age | | Marital status | | Race/ethnicity | | Years of education | |
|---|---|---|---|---|---|---|---|---|
| | (1) | (2) | (3) | (4) | (5) | (6) | (7) | (8) |
| | ≤ 24 years | ≥ 25 years | Partner | No Partner | Black or Brown | White | 11 or less | 12 or more |
| Unemployment rate months 10 to 12 before birth | 0.9963 | 1.0037 | 1.0029 | 0.9971 | 0.9975 | 1.0032 | 0.9954 | 1.0047 |
| 95% CI | (0.9915 - 1.0010) | (0.9990 - 1.0085) | (0.9879 - 1.0181) | (0.9822 - 1.0122) | (0.9834 - 1.0119) | (0.9899 - 1.0166) | (0.9882 - 1.0026) | (0.9974 - 1.0119) |
| p-val | 0.1260 | 0.1260 | 0.7052 | 0.7052 | 0.7340 | 0.6410 | 0.2064 | 0.2064 |
| City of residence (code) | | | | | | | | |
| Porto Velho (1) | | | | | omitted | | | |
| Rio Branco (2) | 0.9699*** | 1.0311*** | 1.7462*** | 0.5727*** | 1.4283*** | 0.7475*** | 0.8166*** | 1.2245*** |
| | (0.9689 - 0.9709) | (1.0300 - 1.0321) | (1.7416 - 1.7508) | (0.5712 - 0.5742) | (1.4144 - 1.4423) | (0.7397 - 0.7554) | (0.8151 - 0.8182) | (1.2222 - 1.2269) |
| | 0.0000 | 0.0000 | 0.0000 | 0.0000 | 0.0000 | 0.0000 | 0.0000 | 0.0000 |
| Manaus (3) | 0.9666*** | 1.0346*** | 0.2506*** | 3.9901*** | 1.6513*** | 0.6483*** | 1.3036*** | 0.7671*** |
| | (0.9532 - 0.9801) | (1.0203 - 1.0491) | (0.2396 - 0.2621) | (3.8151 - 4.1730) | (1.5771 - 1.7289) | (0.6205 - 0.6773) | (1.2763 - 1.3314) | (0.7511 - 0.7835) |
| | 0.0000 | 0.0000 | 0.0000 | 0.0000 | 0.0000 | 0.0000 | 0.0000 | 0.0000 |
| Boa Vista (4) | 0.9602*** | 1.0414*** | 0.8298*** | 1.2051*** | 1.3697*** | 0.3923*** | 0.8509*** | 1.1752*** |
| | (0.9527 - 0.9679) | (1.0332 - 1.0497) | (0.8095 - 0.8505) | (1.1757 - 1.2353) | (1.3368 - 1.4034) | (0.3839 - 0.4009) | (0.8413 - 0.8607) | (1.1619 - 1.1886) |
| | 0.0000 | 0.0000 | 0.0000 | 0.0000 | 0.0000 | 0.0000 | 0.0000 | 0.0000 |
| Belem (5) | 0.9373*** | 1.0669*** | 0.7605*** | 1.3149*** | 1.3689*** | 0.7718*** | 1.0715*** | 0.9333*** |
| | (0.9214 - 0.9535) | (1.0488 - 1.0853) | (0.7208 - 0.8025) | (1.2461 - 1.3874) | (1.2990 - 1.4425) | (0.7347 - 0.8108) | (1.0442 - 1.0995) | (0.9095 - 0.9577) |
| | 0.0000 | 0.0000 | 0.0000 | 0.0000 | 0.0000 | 0.0000 | 0.0000 | 0.0000 |
| Macapa (6) | 1.0984*** | 0.9104*** | 2.2080*** | 0.4529*** | 1.9216*** | 0.5311*** | 1.2395*** | 0.8068*** |
| | (1.0732 - 1.1242) | (0.8896 - 0.9318) | (2.0592 - 2.3676) | (0.4224 - 0.4856) | (1.7919 - 2.0607) | (0.4975 - 0.5669) | (1.1965 - 1.2839) | (0.7789 - 0.8358) |
| | 0.0000 | 0.0000 | 0.0000 | 0.0000 | 0.0000 | 0.0000 | 0.0000 | 0.0000 |
| Palmas (7) | 0.5914*** | 1.6908*** | 2.0928*** | 0.4778*** | 0.7164*** | 1.3478*** | 0.4354*** | 2.2966*** |
| | (0.5849 - 0.5980) | (1.6722 - 1.7097) | (2.0187 - 2.1696) | (0.4609 - 0.4954) | (0.6932 - 0.7405) | (1.3064 - 1.3905) | (0.4284 - 0.4426) | (2.2593 - 2.3345) |
| | 0.0000 | 0.0000 | 0.0000 | 0.0000 | 0.0000 | 0.0000 | 0.0000 | 0.0000 |

| | | | | | | | | |
|---|---|---|---|---|---|---|---|---|
| São Luis (8) | 0.7704*** | 1.2980*** | 1.0808* | 0.9252* | 1.1656*** | 0.9227** | 0.9732 | 1.0275 |
| | (0.7510 - 0.7902) | (1.2654 - 1.3315) | (0.9987 - 1.1696) | (0.8550 - 1.0013) | (1.0803 - 1.2577) | (0.8593 - 0.9907) | (0.9365 - 1.0114) | (0.9887 - 1.0679) |
| | 0.0000 | 0.0000 | 0.0538 | 0.0538 | 0.0001 | 0.0265 | 0.1673 | 0.1673 |
| Teresina (9) | 0.5847*** | 1.7102*** | 1.8247*** | 0.5480*** | 0.8176*** | 1.2625*** | 0.7317*** | 1.3667*** |
| | (0.5830 - 0.5865) | (1.7051 - 1.7152) | (1.8083 - 1.8413) | (0.5431 - 0.5530) | (0.8113 - 0.8240) | (1.2533 - 1.2718) | (0.7287 - 0.7347) | (1.3612 - 1.3723) |
| | 0.0000 | 0.0000 | 0.0000 | 0.0000 | 0.0000 | 0.0000 | 0.0000 | 0.0000 |
| Fortaleza (10) | 0.6895*** | 1.4503*** | 0.8055*** | 1.2415*** | 1.5194*** | 0.7179*** | 0.8794*** | 1.1372*** |
| | (0.6889 - 0.6902) | (1.4489 - 1.4516) | (0.8034 - 0.8075) | (1.2384 - 1.2447) | (1.5097 - 1.5292) | (0.7131 - 0.7227) | (0.8778 - 0.8809) | (1.1351 - 1.1393) |
| | 0.0000 | 0.0000 | 0.0000 | 0.0000 | 0.0000 | 0.0000 | 0.0000 | 0.0000 |
| Natal (11) | 0.6677*** | 1.4977*** | 1.3129*** | 0.7617*** | 0.3985*** | 2.7479*** | 0.7679*** | 1.3023*** |
| | (0.6622 - 0.6733) | (1.4853 - 1.5102) | (1.2778 - 1.3490) | (0.7413 - 0.7826) | (0.3881 - 0.4093) | (2.6799 - 2.8176) | (0.7578 - 0.7781) | (1.2852 - 1.3196) |
| | 0.0000 | 0.0000 | 0.0000 | 0.0000 | 0.0000 | 0.0000 | 0.0000 | 0.0000 |
| Joao Pessoa (12) | 0.6401*** | 1.5623*** | 3.6584*** | 0.2733*** | 0.5719*** | 1.8702*** | 0.6610*** | 1.5129*** |
| | (0.6357 - 0.6445) | (1.5516 - 1.5730) | (3.5719 - 3.7469) | (0.2669 - 0.2800) | (0.5601 - 0.5841) | (1.8336 - 1.9075) | (0.6540 - 0.6681) | (1.4968 - 1.5291) |
| | 0.0000 | 0.0000 | 0.0000 | 0.0000 | 0.0000 | 0.0000 | 0.0000 | 0.0000 |
| Recife (13) | 0.6309*** | 1.5851*** | 0.5440*** | 1.8382*** | 0.4835*** | 2.2535*** | 0.6382*** | 1.5669*** |
| | (0.6247 - 0.6371) | (1.5696 - 1.6008) | (0.5272 - 0.5614) | (1.7813 - 1.8969) | (0.4687 - 0.4987) | (2.1884 - 2.3206) | (0.6284 - 0.6481) | (1.5429 - 1.5913) |
| | 0.0000 | 0.0000 | 0.0000 | 0.0000 | 0.0000 | 0.0000 | 0.0000 | 0.0000 |
| Maceio (14) | 0.9588*** | 1.0429*** | 0.8009*** | 1.2486*** | 1.5623*** | 0.6969*** | 1.0078 | 0.9922 |
| | (0.9439 - 0.9740) | (1.0267 - 1.0595) | (0.7620 - 0.8417) | (1.1881 - 1.3123) | (1.4881 - 1.6402) | (0.6658 - 0.7294) | (0.9840 - 1.0323) | (0.9687 - 1.0163) |
| | 0.0000 | 0.0000 | 0.0000 | 0.0000 | 0.0000 | 0.0000 | 0.5237 | 0.5237 |
| Aracaju (15) | 0.6251*** | 1.5998*** | 1.6847*** | 0.5936*** | 0.8688*** | 1.0698*** | 0.6928*** | 1.4433*** |
| | (0.6159 - 0.6344) | (1.5764 - 1.6236) | (1.6059 - 1.7673) | (0.5658 - 0.6227) | (0.8302 - 0.9093) | (1.0249 - 1.1166) | (0.6774 - 0.7086) | (1.4112 - 1.4762) |
| | 0.0000 | 0.0000 | 0.0000 | 0.0000 | 0.0000 | 0.0020 | 0.0000 | 0.0000 |
| Salvador (16) | 0.5184*** | 1.9290*** | 0.4663*** | 2.1446*** | 1.5424*** | 0.6749*** | 0.7972*** | 1.2543*** |
| | (0.5174 - 0.5194) | (1.9253 - 1.9326) | (0.4631 - 0.4695) | (2.1301 - 2.1592) | (1.5344 - 1.5505) | (0.6717 - 0.6782) | (0.7948 - 0.7997) | (1.2505 - 1.2581) |
| | 0.0000 | 0.0000 | 0.0000 | 0.0000 | 0.0000 | 0.0000 | 0.0000 | 0.0000 |
| Belo Horizonte (17) | 0.4298*** | 2.3267*** | 0.5334*** | 1.8748*** | 0.3458*** | 2.9894*** | 0.5066*** | 1.9741*** |
| | (0.4251 - 0.4345) | (2.3015 - 2.3522) | (0.5153 - 0.5521) | (1.8112 - 1.9407) | (0.3351 - 0.3567) | (2.9038 - 3.0776) | (0.4983 - 0.5150) | (1.9418 - 2.0069) |
| | 0.0000 | 0.0000 | 0.0000 | 0.0000 | 0.0000 | 0.0000 | 0.0000 | 0.0000 |
| Vitoria (18) | 0.4771*** | 2.0962*** | 0.4359*** | 2.2943*** | 0.4740*** | 2.3218*** | 0.4174*** | 2.3960*** |
| | (0.4753 - 0.4789) | (2.0883 - 2.1041) | (0.4307 - 0.4411) | (2.2671 - 2.3218) | (0.4695 - 0.4785) | (2.3019 - 2.3420) | (0.4150 - 0.4197) | (2.3826 - 2.4095) |
| | 0.0000 | 0.0000 | 0.0000 | 0.0000 | 0.0000 | 0.0000 | 0.0000 | 0.0000 |

| | | | | | | | | |
|---|---|---|---|---|---|---|---|---|
| Rio de Janeiro (19) | 0.6290*** | 1.5899*** | 0.2799*** | 3.5723*** | 0.3124*** | 3.4515*** | 0.7222*** | 1.3847*** |
| | (0.6271 - 0.6308) | (1.5852 - 1.5946) | (0.2773 - 0.2826) | (3.5392 - 3.6057) | (0.3100 - 0.3148) | (3.4270 - 3.4761) | (0.7190 - 0.7254) | (1.3785 - 1.3908) |
| | 0.0000 | 0.0000 | 0.0000 | 0.0000 | 0.0000 | 0.0000 | 0.0000 | 0.0000 |
| São Paulo (20) | 0.5306*** | 1.8848*** | 0.6502*** | 1.5380*** | 0.1647*** | 6.2472*** | 0.6341*** | 1.5771*** |
| | (0.5263 - 0.5349) | (1.8695 - 1.9002) | (0.6336 - 0.6672) | (1.4989 - 1.5782) | (0.1609 - 0.1686) | (6.1132 - 6.3841) | (0.6264 - 0.6418) | (1.5580 - 1.5965) |
| | 0.0000 | 0.0000 | 0.0000 | 0.0000 | 0.0000 | 0.0000 | 0.0000 | 0.0000 |
| Curitiba (21) | 0.5290*** | 1.8902*** | 0.5140*** | 1.9456*** | 0.0346*** | 29.9378*** | 0.4439*** | 2.2529*** |
| | (0.5200 - 0.5383) | (1.8578 - 1.9232) | (0.4863 - 0.5432) | (1.8410 - 2.0562) | (0.0329 - 0.0364) | (28.5642 - 31.3775) | (0.4324 - 0.4557) | (2.1945 - 2.3129) |
| | 0.0000 | 0.0000 | 0.0000 | 0.0000 | 0.0000 | 0.0000 | 0.0000 | 0.0000 |
| Florianopolis (22) | 0.5036*** | 1.9858*** | 1.2457*** | 0.8028*** | 0.0286*** | 38.0379*** | 0.3993*** | 2.5041*** |
| | (0.4916 - 0.5158) | (1.9386 - 2.0343) | (1.1540 - 1.3446) | (0.7437 - 0.8665) | (0.0266 - 0.0307) | (35.5751 - 40.6712) | (0.3851 - 0.4141) | (2.4146 - 2.5969) |
| | 0.0000 | 0.0000 | 0.0000 | 0.0000 | 0.0000 | 0.0000 | 0.0000 | 0.0000 |
| Porto Alegre (23) | 0.5562*** | 1.7981*** | 0.4756*** | 2.1026*** | 0.0651*** | 16.6524*** | 0.5714*** | 1.7500*** |
| | (0.5474 - 0.5650) | (1.7698 - 1.8268) | (0.4525 - 0.4999) | (2.0004 - 2.2100) | (0.0621 - 0.0682) | (15.9473 - 17.3886) | (0.5581 - 0.5851) | (1.7091 - 1.7919) |
| | 0.0000 | 0.0000 | 0.0000 | 0.0000 | 0.0000 | 0.0000 | 0.0000 | 0.0000 |
| Campo Grande (24) | 0.7651*** | 1.3071*** | 0.3882*** | 2.5758*** | 0.3346*** | 3.1426*** | 0.6280*** | 1.5924*** |
| | (0.7615 - 0.7686) | (1.3010 - 1.3132) | (0.3826 - 0.3940) | (2.5382 - 2.6140) | (0.3303 - 0.3390) | (3.1051 - 3.1806) | (0.6236 - 0.6324) | (1.5813 - 1.6035) |
| | 0.0000 | 0.0000 | 0.0000 | 0.0000 | 0.0000 | 0.0000 | 0.0000 | 0.0000 |
| Cuiaba (25) | 0.6824*** | 1.4654*** | 1.9636*** | 0.5093*** | 0.6127*** | 1.7225*** | 0.5456*** | 1.8329*** |
| | (0.6720 - 0.6930) | (1.4429 - 1.4881) | (1.8712 - 2.0605) | (0.4853 - 0.5344) | (0.5862 - 0.6403) | (1.6533 - 1.7946) | (0.5332 - 0.5582) | (1.7915 - 1.8754) |
| | 0.0000 | 0.0000 | 0.0000 | 0.0000 | 0.0000 | 0.0000 | 0.0000 | 0.0000 |
| Goiania (26) | 0.5818*** | 1.7189*** | 1.5606*** | 0.6408*** | 0.2632*** | 4.0653*** | 0.5148*** | 1.9425*** |
| | (0.5718 - 0.5920) | (1.6893 - 1.7490) | (1.4773 - 1.6486) | (0.6066 - 0.6769) | (0.2503 - 0.2768) | (3.8797 - 4.2598) | (0.5016 - 0.5284) | (1.8926 - 1.9936) |
| | 0.0000 | 0.0000 | 0.0000 | 0.0000 | 0.0000 | 0.0000 | 0.0000 | 0.0000 |
| Brasilia (27) | 0.5383*** | 1.8578*** | 0.7193*** | 1.3903*** | 0.4760*** | 2.2511*** | 0.5260*** | 1.9013*** |
| | (0.5367 - 0.5398) | (1.8526 - 1.8631) | (0.7133 - 0.7253) | (1.3788 - 1.4019) | (0.4725 - 0.4796) | (2.2356 - 2.2668) | (0.5238 - 0.5281) | (1.8935 - 1.9092) |
| | 0.0000 | 0.0000 | 0.0000 | 0.0000 | 0.0000 | 0.0000 | 0.0000 | 0.0000 |
| Month | | | | | | | | |
| 1 | | | | | omitted | | | |
| 2 | 0.9741* | 1.0266* | 0.9569*** | 1.0450*** | 1.1285*** | 0.9434*** | 1.2066*** | 0.8288*** |
| | (0.9486 - 1.0003) | (0.9997 - 1.0542) | (0.9270 - 0.9879) | (1.0123 - 1.0788) | (1.1061 - 1.1514) | (0.9256 - 0.9615) | (1.1840 - 1.2296) | (0.8133 - 0.8446) |
| | 0.0526 | 0.0526 | 0.0067 | 0.0067 | 0.0000 | 0.0000 | 0.0000 | 0.0000 |
| 3 | 0.9150*** | 1.0929*** | 1.0370* | 0.9643* | 0.9252*** | 1.1054*** | 1.0204* | 0.9800* |

| | | | | | | | | |
|---|---|---|---|---|---|---|---|---|
| | (0.8923 - 0.9383) | (1.0657 - 1.1207) | (0.9990 - 1.0764) | (0.9290 - 1.0010) | (0.9038 - 0.9470) | (1.0805 - 1.1310) | (0.9988 - 1.0425) | (0.9592 - 1.0012) |
| | 0.0000 | 0.0000 | 0.0562 | 0.0562 | 0.0000 | 0.0000 | 0.0649 | 0.0649 |
| 4 | 0.9428*** | 1.0607*** | 0.9130*** | 1.0953*** | 0.8935*** | 1.1645*** | 0.9657*** | 1.0355*** |
| | (0.9229 - 0.9631) | (1.0383 - 1.0836) | (0.8814 - 0.9457) | (1.0575 - 1.1345) | (0.8645 - 0.9236) | (1.1279 - 1.2023) | (0.9501 - 0.9815) | (1.0189 - 1.0525) |
| | 0.0000 | 0.0000 | 0.0000 | 0.0000 | 0.0000 | 0.0000 | 0.0000 | 0.0000 |
| 5 | 0.9980 | 1.0020 | 0.9689 | 1.0321 | 1.0173 | 1.0154 | 0.9607*** | 1.0410*** |
| | (0.9761 - 1.0203) | (0.9801 - 1.0245) | (0.9308 - 1.0086) | (0.9915 - 1.0743) | (0.9771 - 1.0591) | (0.9727 - 1.0600) | (0.9370 - 0.9850) | (1.0153 - 1.0673) |
| | 0.8560 | 0.8560 | 0.1227 | 0.1227 | 0.4040 | 0.4856 | 0.0016 | 0.0016 |
| 6 | 0.9258*** | 1.0802*** | 1.0304 | 0.9705 | 1.2530*** | 0.8246*** | 0.9950 | 1.0050 |
| | (0.9103 - 0.9415) | (1.0621 - 1.0985) | (0.9886 - 1.0740) | (0.9311 - 1.0115) | (1.2033 - 1.3047) | (0.7920 - 0.8587) | (0.9755 - 1.0149) | (0.9853 - 1.0251) |
| | 0.0000 | 0.0000 | 0.1565 | 0.1565 | 0.0000 | 0.0000 | 0.6190 | 0.6190 |
| 7 | 1.0341** | 0.9670** | 0.9325* | 1.0724* | 1.1602*** | 0.9113*** | 1.0894*** | 0.9180*** |
| | (1.0052 - 1.0640) | (0.9399 - 0.9948) | (0.8682 - 1.0016) | (0.9984 - 1.1518) | (1.1075 - 1.2153) | (0.8733 - 0.9509) | (1.0488 - 1.1315) | (0.8838 - 0.9535) |
| | 0.0205 | 0.0205 | 0.0552 | 0.0552 | 0.0000 | 0.0000 | 0.0000 | 0.0000 |
| 8 | 1.0410*** | 0.9606*** | 0.8942*** | 1.1183*** | 1.0593** | 0.9911 | 1.1182*** | 0.8943*** |
| | (1.0163 - 1.0663) | (0.9378 - 0.9840) | (0.8231 - 0.9716) | (1.0293 - 1.2150) | (1.0090 - 1.1121) | (0.9469 - 1.0374) | (1.0715 - 1.1668) | (0.8570 - 0.9332) |
| | 0.0011 | 0.0011 | 0.0082 | 0.0082 | 0.0202 | 0.7008 | 0.0000 | 0.0000 |
| 9 | 1.0449*** | 0.9571*** | 0.9738 | 1.0269 | 1.0408 | 0.9820 | 1.0235 | 0.9770 |
| | (1.0112 - 1.0797) | (0.9262 - 0.9889) | (0.9060 - 1.0466) | (0.9555 - 1.1037) | (0.9799 - 1.1055) | (0.9237 - 1.0441) | (0.9885 - 1.0598) | (0.9436 - 1.0116) |
| | 0.0087 | 0.0087 | 0.4702 | 0.4702 | 0.1937 | 0.5621 | 0.1903 | 0.1903 |
| 10 | 0.9357*** | 1.0687*** | 0.9564 | 1.0456 | 0.9629 | 1.0806*** | 1.0195 | 0.9808 |
| | (0.9085 - 0.9638) | (1.0376 - 1.1007) | (0.8955 - 1.0215) | (0.9789 - 1.1167) | (0.9130 - 1.0155) | (1.0273 - 1.1366) | (0.9907 - 1.0492) | (0.9531 - 1.0094) |
| | 0.0000 | 0.0000 | 0.1849 | 0.1849 | 0.1638 | 0.0027 | 0.1868 | 0.1868 |
| 11 | 1.0116 | 0.9885 | 0.9059*** | 1.1038*** | 1.0809*** | 0.9571* | 0.9952 | 1.0048 |
| | (0.9791 - 1.0452) | (0.9567 - 1.0213) | (0.8430 - 0.9735) | (1.0272 - 1.1862) | (1.0273 - 1.1372) | (0.9106 - 1.0060) | (0.9650 - 1.0264) | (0.9743 - 1.0363) |
| | 0.4870 | 0.4870 | 0.0071 | 0.0071 | 0.0027 | 0.0845 | 0.7599 | 0.7599 |
| 12 | 0.9800 | 1.0204 | 0.8594*** | 1.1636*** | 1.0602*** | 0.9761 | 1.0723*** | 0.9326*** |
| | (0.9559 - 1.0046) | (0.9954 - 1.0462) | (0.8128 - 0.9086) | (1.1006 - 1.2303) | (1.0200 - 1.1020) | (0.9426 - 1.0107) | (1.0520 - 1.0929) | (0.9150 - 0.9505) |
| | 0.1108 | 0.1108 | 0.0000 | 0.0000 | 0.0030 | 0.1728 | 0.0000 | 0.0000 |
| Year | | | | | | | | |
| 2013 | | | | | omitted | | | |
| 2014 | 1.0291** | 0.9718** | 1.0544 | 0.9485 | 1.0442 | 0.9595 | 1.2133*** | 0.8242*** |
| | (1.0057 - 1.0530) | (0.9497 - 0.9943) | (0.9855 - 1.1280) | (0.8865 - 1.0147) | (0.9909 - 1.1003) | (0.9130 - 1.0083) | (1.1756 - 1.2522) | (0.7986 - 0.8506) |

| | | | | | | | | |
|---|---|---|---|---|---|---|---|---|
| | 0.0145 | 0.0145 | 0.1246 | 0.1246 | 0.1053 | 0.1027 | 0.0000 | 0.0000 |
| 2015 | 1.0322 | 0.9688 | 0.8756*** | 1.1421*** | 0.7962*** | 1.2615*** | 0.9284*** | 1.0772*** |
| | (0.9919 - 1.0742) | (0.9309 - 1.0082) | (0.8120 - 0.9441) | (1.0592 - 1.2316) | (0.7564 - 0.8382) | (1.2035 - 1.3222) | (0.8962 - 0.9617) | (1.0399 - 1.1158) |
| | 0.1191 | 0.1191 | 0.0006 | 0.0006 | 0.0000 | 0.0000 | 0.0000 | 0.0000 |
| 2016 | 0.9799** | 1.0205** | 0.7693*** | 1.2998*** | 0.8852*** | 1.1333*** | 0.7595*** | 1.3166*** |
| | (0.9647 - 0.9953) | (1.0047 - 1.0366) | (0.7382 - 0.8018) | (1.2472 - 1.3547) | (0.8517 - 0.9201) | (1.0940 - 1.1740) | (0.7361 - 0.7838) | (1.2759 - 1.3586) |
| | 0.0107 | 0.0107 | 0.0000 | 0.0000 | 0.0000 | 0.0000 | 0.0000 | 0.0000 |
| | | | | | | | | |
| Constant | 0.9908 | 1.0093 | 2.0388*** | 0.4905*** | 5.3253*** | 0.1702*** | 4.4421*** | 0.2251*** |
| | (0.9544 - 1.0286) | (0.9722 - 1.0478) | (1.8535 - 2.2427) | (0.4459 - 0.5395) | (4.8298 - 5.8717) | (0.1555 - 0.1864) | (4.1996 - 4.6985) | (0.2128 - 0.2381) |
| | 0.6281 | 0.6281 | 0.0000 | 0.0000 | 0.0000 | 0.0000 | 0.0000 | 0.0000 |
| | | | | | | | | |
| Observations | 2,427,273 | 2,427,273 | 2,408,605 | 2,408,605 | 2,289,931 | 2,289,931 | 2,404,774 | 2,404,774 |

Note: coefficients of interaction terms are not reported. *** p<0.01, ** p<0.05, * p<0.1
