# Supplementary material for: In utero exposure to economic fluctuations and birth outcomes: An analysis of the relevance of the local unemployment rate in Brazilian state capitals
Source: PLoS One. 2019 Oct 10;14(10):e0223673. doi: 10.1371/journal.pone.0223673 (PMC6786569; doi:10.1371/journal.pone.0223673)
Supplement: S5 Table — (PDF) [file pone.0223673.s005.pdf]

S5 Table. False Discovery Rate controlling procedure

Rank	Table	Column	Independent variable	Dependent variable	p-value	BH p-value	p-value ≤ BH p-value?
1	Table 4	1	Unemployment rate months 1 to 3 before birth	VLBW	0	0.0005	yes
2	Table 4	7	Unemployment rate months 1 to 3 before birth	VLBW	0	0.0010	yes
3	Table 4	5	Unemployment rate months 1 to 3 before birth	VLBW	0.0009	0.0015	yes
4	Table 4	1	Unemployment rate months 7 to 9 before birth	VLBW	0.0116	0.0020	no
5	Table 3	6	Unemployment rate months 1 to 3 before birth	VLBW	0.0278	0.0025	no
6	Table 2 - Panel B	1	Unemployment rate months 1 to 3 before birth	Birthweight (grams)	0.0314	0.0030	no
7	Table 3	3	Unemployment rate months 1 to 3 before birth	VLBW	0.0372	0.0035	no
8	Table 3	7	Unemployment rate months 1 to 3 before birth	Birthweight	0.0382	0.0040	no
9	Table 2 - Panel B	3	Unemployment rate months 1 to 3 before birth	VLBW	0.0447	0.0045	no
10	Table 3	4	Unemployment rate months 1 to 3 before birth	Birthweight	0.0484	0.0051	no
11	Table 3	9	Unemployment rate months 1 to 3 before birth	VLBW	0.0493	0.0056	no
12	Table 3	1	Unemployment rate months 1 to 3 before birth	Birthweight	0.0601	0.0061	no
13	Table 2 - Panel B	6	Unemployment rate months 1 to 3 before birth	VPTB	0.0613	0.0066	no
14	Table 2 - Panel A	4	Unemployment rate months 1 to 9 before birth	Gestational Age (weeks)	0.0712	0.0071	no
15	Table 4	3	Unemployment rate months 1 to 3 before birth	VLBW	0.0965	0.0076	no
16	Table 4	7	Unemployment rate months 4 to 6 before birth	VLBW	0.0986	0.0081	no
17	Table 2 - Panel A	6	Unemployment rate months 1 to 9 before birth	VPTB	0.1223	0.0086	no
18	Table 5	1	Unemployment rate months 10 to 12 before birth	<= 24 years	0.126	0.0091	no
19	Table 5	2	Unemployment rate months 10 to 12 before birth	>= 25 years	0.126	0.0096	no
20	Table 2 - Panel B	4	Unemployment rate months 7 to 9 before birth	Gestational Age (weeks)	0.1302	0.0101	no
21	Table 4	3	Unemployment rate months 7 to 9 before birth	VLBW	0.1338	0.0106	no
22	Table 2 - Panel B	3	Unemployment rate months 4 to 6 before birth	VLBW	0.1445	0.0111	no
23	Table 3	9	Unemployment rate months 4 to 6 before birth	VLBW	0.1451	0.0116	no
24	Table 4	7	Unemployment rate months 7 to 9 before birth	VLBW	0.1528	0.0121	no
25	Table 5	5	Unemployment rate months 10 to 18 before birth	Black or brown	0.1646	0.0126	no
26	Table 4	1	Unemployment rate months 4 to 6 before birth	VLBW	0.1732	0.0131	no
27	Table 3	3	Unemployment rate months 4 to 6 before birth	VLBW	0.1821	0.0136	no
28	Table 4	8	Unemployment rate months 1 to 3 before birth	VLBW	0.195	0.0141	no
29	Table 5	7	Unemployment rate months 10 to 12 before birth	11 or less years of education	0.2064	0.0146	no
30	Table 5	8	Unemployment rate months 10 to 12 before birth	12 or more years of education	0.2064	0.0152	no
31	Table 2 - Panel A	1	Unemployment rate months 1 to 9 before birth	Birthweight (grams)	0.2146	0.0157	no
32	Table 2 - Panel B	6	Unemployment rate months 7 to 9 before birth	VPTB	0.222	0.0162	no
33	Table 4	2	Unemployment rate months 7 to 9 before birth	VLBW	0.2228	0.0167	no
34	Table 3	6	Unemployment rate months 4 to 6 before birth	VLBW	0.2258	0.0172	no
35	Table 2 - Panel B	5	Unemployment rate months 4 to 6 before birth	PTB	0.2337	0.0177	no
36	Table 4	4	Unemployment rate months 4 to 6 before birth	VLBW	0.2348	0.0182	no
37	Table 2 - Panel B	7	Unemployment rate months 4 to 6 before birth	SGA	0.2393	0.0187	no
38	Table 2 - Panel A	7	Unemployment rate months 1 to 9 before birth	SGA	0.2482	0.0192	no
39	Table 5	6	Unemployment rate months 10 to 18 before birth	White	0.2627	0.0197	no
40	Table 2 - Panel A	2	Unemployment rate months 1 to 9 before birth	LBW	0.2749	0.0202	no
41	Table 2 - Panel A	8	Unemployment rate months 1 to 9 before birth	Girl	0.2874	0.0207	no
42	Table 4	8	Unemployment rate months 7 to 9 before birth	VLBW	0.312	0.0212	no
43	Table 2 - Panel B	5	Unemployment rate months 7 to 9 before birth	PTB	0.3215	0.0217	no
44	Table 3	5	Unemployment rate months 1 to 3 before birth	LBW	0.342	0.0222	no
45	Table 2 - Panel B	2	Unemployment rate months 7 to 9 before birth	LBW	0.3597	0.0227	no
46	Table 3	8	Unemployment rate months 7 to 9 before birth	LBW	0.3714	0.0232	no
47	Table 3	2	Unemployment rate months 1 to 3 before birth	LBW	0.3909	0.0237	no
48	Table 4	5	Unemployment rate months 7 to 9 before birth	VLBW	0.3938	0.0242	no
49	Table 3	3	Unemployment rate months 7 to 9 before birth	VLBW	0.4004	0.0247	no
50	Table 4	5	Unemployment rate months 4 to 6 before birth	VLBW	0.4036	0.0253	no
51	Table 2 - Panel B	8	Unemployment rate months 1 to 3 before birth	Girl	0.4169	0.0258	no
52	Table 4	6	Unemployment rate months 4 to 6 before birth	VLBW	0.443	0.0263	no
53	Table 3	9	Unemployment rate months 7 to 9 before birth	VLBW	0.4437	0.0268	no
54	Table 2 - Panel B	3	Unemployment rate months 7 to 9 before birth	VLBW	0.4468	0.0273	no
55	Table 4	3	Unemployment rate months 4 to 6 before birth	VLBW	0.4626	0.0278	no
56	Table 2 - Panel B	1	Unemployment rate months 7 to 9 before birth	Birthweight (grams)	0.4679	0.0283	no
57	Table 2 - Panel B	7	Unemployment rate months 1 to 3 before birth	SGA	0.4725	0.0288	no

58	Table 3	1	Unemployment rate months 4 to 6 before birth	Birthweight	0.4873	0.0293	no
59	Table 3	7	Unemployment rate months 7 to 9 before birth	Birthweight	0.5005	0.0298	no
60	Table 2 - Panel B	5	Unemployment rate months 1 to 3 before birth	PTB	0.5126	0.0303	no
61	Table 3	6	Unemployment rate months 7 to 9 before birth	VLBW	0.514	0.0308	no
62	Table 2 - Panel B	4	Unemployment rate months 4 to 6 before birth	Gestational Age (weeks)	0.5424	0.0313	no
63	Table 5	3	Unemployment rate months 10 to 18 before birth	Partner	0.5449	0.0318	no
64	Table 5	4	Unemployment rate months 10 to 18 before birth	No partner	0.5449	0.0323	no
65	Table 2 - Panel B	4	Unemployment rate months 1 to 3 before birth	Gestational Age (weeks)	0.5553	0.0328	no
66	Table 2 - Panel A	3	Unemployment rate months 1 to 9 before birth	VLBW	0.5588	0.0333	no
67	Table 2 - Panel B	2	Unemployment rate months 1 to 3 before birth	LBW	0.565	0.0338	no
68	Table 3	1	Unemployment rate months 7 to 9 before birth	Birthweight	0.5774	0.0343	no
69	Table 3	8	Unemployment rate months 1 to 3 before birth	LBW	0.5806	0.0348	no
70	Table 3	4	Unemployment rate months 4 to 6 before birth	Birthweight	0.5898	0.0354	no
71	Table 3	4	Unemployment rate months 7 to 9 before birth	Birthweight	0.5927	0.0359	no
72	Table 3	5	Unemployment rate months 7 to 9 before birth	LBW	0.5987	0.0364	no
73	Table 4	4	Unemployment rate months 1 to 3 before birth	VLBW	0.6049	0.0369	no
74	Table 4	6	Unemployment rate months 7 to 9 before birth	VLBW	0.6145	0.0374	no
75	Table 5	1	Unemployment rate months 10 to 18 before birth	<= 24 years	0.6203	0.0379	no
76	Table 5	2	Unemployment rate months 10 to 18 before birth	>= 25 years	0.6203	0.0384	no
77	Table 5	6	Unemployment rate months 10 to 12 before birth	White	0.641	0.0389	no
78	Table 2 - Panel A	5	Unemployment rate months 1 to 9 before birth	PTB	0.649	0.0394	no
79	Table 2 - Panel B	1	Unemployment rate months 4 to 6 before birth	Birthweight (grams)	0.6575	0.0399	no
80	Table 3	7	Unemployment rate months 4 to 6 before birth	Birthweight	0.6601	0.0404	no
81	Table 4	2	Unemployment rate months 4 to 6 before birth	VLBW	0.6635	0.0409	no
82	Table 3	2	Unemployment rate months 7 to 9 before birth	LBW	0.6827	0.0414	no
83	Table 5	3	Unemployment rate months 10 to 12 before birth	Partner	0.7052	0.0419	no
84	Table 5	4	Unemployment rate months 10 to 12 before birth	No partner	0.7052	0.0424	no
85	Table 2 - Panel B	8	Unemployment rate months 4 to 6 before birth	Girl	0.7059	0.0429	no
86	Table 5	5	Unemployment rate months 10 to 12 before birth	Black or brown	0.734	0.0434	no
87	Table 2 - Panel B	6	Unemployment rate months 4 to 6 before birth	VPTB	0.7416	0.0439	no
88	Table 4	8	Unemployment rate months 4 to 6 before birth	VLBW	0.7912	0.0444	no
89	Table 2 - Panel B	8	Unemployment rate months 7 to 9 before birth	Girl	0.8196	0.0449	no
90	Table 3	8	Unemployment rate months 4 to 6 before birth	LBW	0.8443	0.0455	no
91	Table 2 - Panel B	2	Unemployment rate months 4 to 6 before birth	LBW	0.8489	0.0460	no
92	Table 3	2	Unemployment rate months 4 to 6 before birth	LBW	0.8746	0.0465	no
93	Table 4	6	Unemployment rate months 1 to 3 before birth	VLBW	0.8834	0.0470	no
94	Table 2 - Panel B	7	Unemployment rate months 7 to 9 before birth	SGA	0.8915	0.0475	no
95	Table 3	5	Unemployment rate months 4 to 6 before birth	LBW	0.9084	0.0480	no
96	Table 4	4	Unemployment rate months 7 to 9 before birth	VLBW	0.9169	0.0485	no
97	Table 5	7	Unemployment rate months 10 to 18 before birth	11 or less years of educ	0.9314	0.0490	no
98	Table 5	8	Unemployment rate months 10 to 18 before birth	12 or more years of educ	0.9314	0.0495	no
99	Table 4	2	Unemployment rate months 1 to 3 before birth	VLBW	0.965	0.0500	no

Note: BH p-values considering a False Discovery Rate of 0.05
